# Structure-Guided Identification of a Family of Dual Receptor-Binding PfEMP1 that Is Associated with Cerebral Malaria

**DOI:** 10.1016/j.chom.2017.02.009

**Published:** 2017-03-08

**Authors:** Frank Lennartz, Yvonne Adams, Anja Bengtsson, Rebecca W. Olsen, Louise Turner, Nicaise T. Ndam, Gertrude Ecklu-Mensah, Azizath Moussiliou, Michael F. Ofori, Benoit Gamain, John P. Lusingu, Jens E.V. Petersen, Christian W. Wang, Sofia Nunes-Silva, Jakob S. Jespersen, Clinton K.Y. Lau, Thor G. Theander, Thomas Lavstsen, Lars Hviid, Matthew K. Higgins, Anja T.R. Jensen

**Affiliations:** 1 Department of Biochemistry, University of Oxford, South Parks Road, OX1 3QU Oxford, UK; 2 Centre for Medical Parasitology, Department of Immunology and Microbiology (ISIM), Faculty of Health and Medical Sciences, University of Copenhagen, 1165 Copenhagen, Denmark; 3 Department of Infectious Diseases, Copenhagen University Hospital (Rigshospitalet), 2100 Copenhagen, Denmark; 4 Faculté de Pharmacie, Institut de Recherche pour le Développement (IRD), COMUE Sorbonne Paris Cité, 75013 Paris, France; 5 Faculté des Sciences de la Santé (FSS), Université d’Aboméy Calavi, 01 BP 526 Cotonou, Benin; 6 Department of Immunology, Noguchi Memorial Institute for Medical Research, University of Ghana, Legon, Ghana; 7 UMR_S1134, Université Sorbonne Paris Cité, Université Paris Diderot, Inserm, INTS, Unité Biologie Intégrée du Globule Rouge, Laboratoire d’Excellence GR-Ex, 75013 Paris, France; 8 National Institute for Medical Research, Tanga Centre, 11101 Dar es Salaam, Tanzania

**Keywords:** cerebral malaria, *Plasmodium falciparum*, ICAM-1, EPCR, PfEMP1

## Abstract

Cerebral malaria is a deadly outcome of infection by *Plasmodium falciparum*, occurring when parasite-infected erythrocytes accumulate in the brain. These erythrocytes display parasite proteins of the PfEMP1 family that bind various endothelial receptors. Despite the importance of cerebral malaria, a binding phenotype linked to its symptoms has not been identified. Here, we used structural biology to determine how a group of PfEMP1 proteins interacts with intercellular adhesion molecule 1 (ICAM-1), allowing us to predict binders from a specific sequence motif alone. Analysis of multiple *Plasmodium falciparum* genomes showed that ICAM-1-binding PfEMP1s also interact with endothelial protein C receptor (EPCR), allowing infected erythrocytes to synergistically bind both receptors. Expression of these PfEMP1s, predicted to bind both ICAM-1 and EPCR, is associated with increased risk of developing cerebral malaria. This study therefore reveals an important PfEMP1-binding phenotype that could be targeted as part of a strategy to prevent cerebral malaria.

## Highlights

• Structural basis for *P. falciparum* PfEMP1 binding to endothelial receptor ICAM-1defined
• A sequence motif derived from structure predicts group A PfEMP1 binding to ICAM-1
• These ICAM-1-binding PfEMP1s also all bind to endothelial protein C receptor (EPCR)
• Expression of dual ICAM-1- and EPCR-binding PfEMP1 is associated with cerebral malaria

## Graphical Abstract

**Figure:**
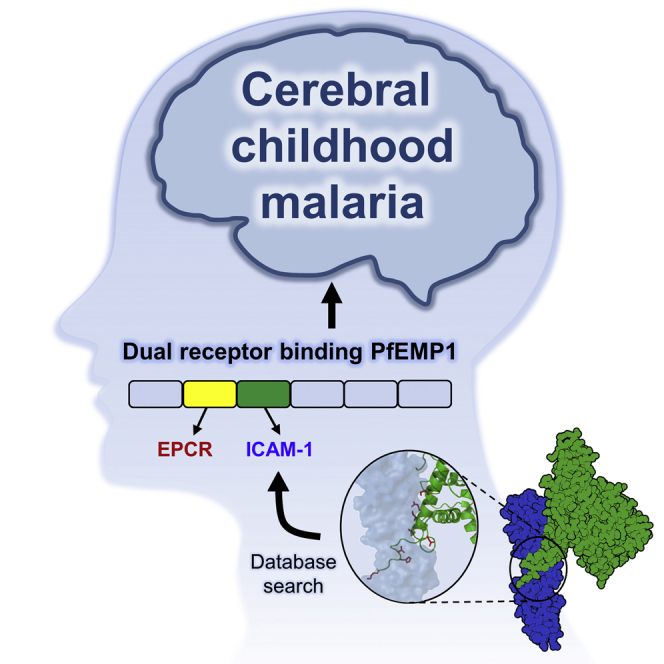

## Teaser

*Plasmodium falciparum*-infected erythrocytes display PfEMP1 proteins that bind various endothelial receptors, including ICAM-1. Lennartz et al. structurally characterize PfEMP1 binding to ICAM-1, allowing them to identify a PfEMP1 family that simultaneously binds to both ICAM-1 and EPCR. Dual-binding PfEMP1s display stronger endothelial adhesion and are associated with cerebral malaria.

## Introduction

Malaria affects hundreds of millions of people each year. While most cases are not life threatening, a significant number of infections by *Plasmodium falciparum* result in severe malaria, manifested in one or more of three major syndromes: anemia, respiratory distress, and cerebral malaria (CM). These occur as parasites infect and divide within human erythrocytes. The infected erythrocytes adhere to blood vessels and tissue surfaces, allowing the parasite to avoid clearance by the spleen. In addition, organ-specific sequestration can have major consequences for development of specific malaria symptoms, particularly in CM, the most debilitating form of the disease. Here, infected erythrocytes accumulate in the brain, occluding blood flow, inducing inflammation, and leading to major neurological complications (Hviid and Jensen, 2015). Even with antimalarial treatment, mortality rates due to CM in children range between 15% and 20% (Dondorp et al., 2010, Seydel et al., 2015), and survivors of CM often suffer from a wide variety of long-lasting neurological damage, which can result in loss of motor function, impairment in learning and language capability, or an increased risk of epilepsy (Birbeck et al., 2010, Idro et al., 2005).

During blood stage infection, the parasite expresses members of different variant surface protein families. Of these, *Plasmodium falciparum* erythrocyte membrane proteins 1 (PfEMP1) are best understood. They are displayed on infected erythrocyte surfaces and tether the cells to various receptors. Each *Plasmodium falciparum* genome contains ∼60 PfEMP1-encoding *var* genes, which can be classified according to their chromosomal context into groups A–E. They have large ectodomains containing multiple duffy binding-like (DBL) and cysteine-rich inter-domain region (CIDR) domains that can interact with specific human endothelial receptors. Sequence analysis allows the CIDR and DBL domains to be divided into general subgroups associated with specific binding phenotypes (Hviid and Jensen, 2015). In particular, subclasses of DBLβ domains found in group A, B, and C PfEMP1 bind ICAM-1 (Bengtsson et al., 2013, Howell et al., 2008, Janes et al., 2011); CIDRα1 domains from group A PfEMP1 bind endothelial protein C receptor (EPCR) (Lau et al., 2015, Turner et al., 2013); and group B and C PfEMP1 contain CIDRα2–6 domains, which bind CD36 (Hsieh et al., 2016, Robinson et al., 2003).

With a number of receptors available to bind to PfEMP1, a major goal has been to determine whether PfEMP1s with specific receptor-binding phenotypes are associated with cerebral and other forms of severe malaria. These studies mostly focused on children, as adults from malaria endemic areas have a lower risk of death from complications during infection. Early studies showed that parasites that express group A PfEMP1 or those that form rosettes are linked with severe malaria (Doumbo et al., 2009, Jensen et al., 2004). The search was further focused with the discovery that severe malaria is associated with a subset of group A and B/A PfEMP1s (Avril et al., 2012, Claessens et al., 2012, Lavstsen et al., 2012), which were later shown to bind to EPCR (Turner et al., 2013). This demonstrated a link between severe malaria and expression of EPCR-binding PfEMP1 (Bernabeu et al., 2016, Jespersen et al., 2016, Turner et al., 2013).

While these studies established associations between severe malaria and particular groups of PfEMP1, no connections have been identified to any specific individual severe malaria syndrome. In particular, attempts to link CM to expression of certain groups of PfEMP1 remain inconclusive. Some studies have correlated cerebral disease with ICAM-1 binding (Ochola et al., 2011, Silamut et al., 1999, Turner et al., 1994), but others found no such link (Newbold et al., 1997, Rogerson et al., 1999). Indeed, ICAM-1-binding DBLβ domains occur in B- and C-type PfEMP1s that are associated with uncomplicated malaria as well as A-type PfEMP1s associated with severe disease, suggesting that ICAM-1 binding alone is not a driver of CM (Bengtsson et al., 2013, Howell et al., 2008, Janes et al., 2011).

A significant obstacle to associating specific adhesion phenotypes with disease outcomes has been the inability to directly predict, using sequence information, adhesion traits of PfEMP1 expressed in patients. While EPCR- and CD36-binding CIDR domains can be predicted from their sequences (Hsieh et al., 2016, Robinson et al., 2003, Turner et al., 2013), ICAM-1-binding domains cannot. We therefore aimed to understand the molecular basis for ICAM-1 binding by A-type PfEMP1, to define a motif allowing the identification of ICAM-1-binding DBLβ domains from sequence alone, and to determine whether the expression of these ICAM-1-binding domains is associated with the development of CM.

## Results

### The Structural Basis for ICAM-1 Binding by PfEMP1

**Figure 1:**
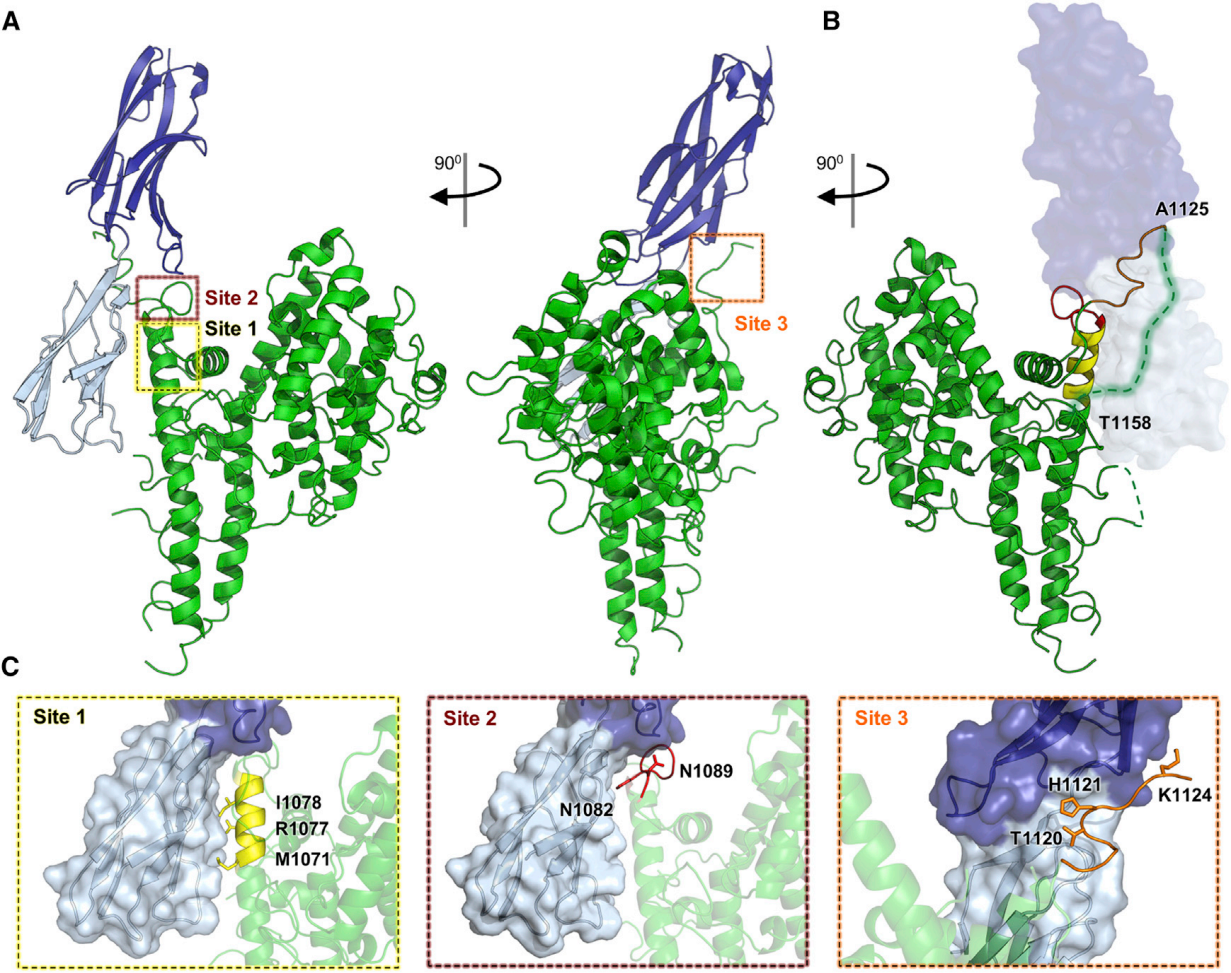
The Structural Basis for ICAM-1 Binding by Group A PfEMP1s (A) Front and side views of the PF11_0521 DBLβ_D4 domain (green) bound to ICAM-1^D1D2^ (D1, light blue; D2, dark blue). Dashed boxes highlight three sites that make direct contact with ICAM-1. (B) Back view of PF11_0521 DBLβ_D4 domain (green) bound to ICAM-1^D1D2^. Dashed lines represent residues not visible in the electron density map with the disordered regions near site 3 (orange) of the ICAM-1-binding site highlighted. (C) Three distinct sites in the PF11_0521 DBLβ_D4 domain directly interact with ICAM-1 with residues that mediate this interaction indicated. See also Figures S1 and S2 and Tables S1 and S2.

In the absence of a structure of a DBLβ domain bound to ICAM-1, we purified complexes of a diverse set of DBLβ domains from group A PfEMP1 bound to the N-terminal two domains of ICAM-1 (ICAM-1^D1D2^), increasing the likelihood of obtaining well-diffracting crystals. Of these, a complex of the PF11_0521 (PlasmoDB: PF3D7_1150400) DBLβ3_D4 domain and ICAM-1^D1D2^ formed crystals that diffracted to 2.8 Å resolution (Table S1). We used molecular replacement, with previous structures of ICAM-1 domains and a model derived from the varO DBLα domain as search models, to determine the structure (Figures 1A and 1B). We also used small-angle X-ray scattering to confirm that the arrangement of the complex was the same in solution as that observed in the crystals, and that it further matched the arrangement of a complex of the DBLβ domain bound to full-length ICAM-1^D1D5^ (Figure S1).

**Figure 2:**
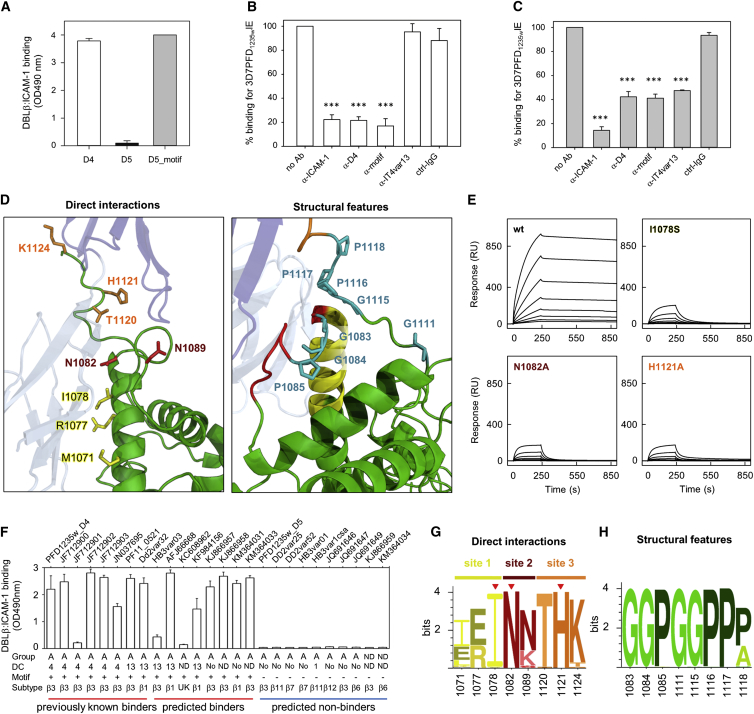
Structure-Guided Identification of a Motif that Predicts ICAM-1 Binding (A) ICAM-1 binding (ELISA OD 490 nm; ± SD) of recombinant 3D7 PFD1235w DBLβ3_D4, PFD1235w DBLβ3_D5, and a chimeric DBLβ3_D5 containing the ICAM-1-binding motif region of DBLβ3_D4 (D5_motif). (B and C) Inhibition of 3D7 PFD1235W infected erythrocyte adhering to ICAM-1 under flow in the presence of anti-ICAM-1 Abs, affinity-purified anti-PFD1235w DBLβ3_D4 (anti-D4) IgG, anti-PFD1235w_motif IgG, anti-ITVAR13, and control IgG. IgG antibodies affinity purified from (B) rat anti-serum and (C) human serum. ± SD of a minimum of three independent experiments done in triplicate. Data were analyzed using one-way ANOVA. Asterisks indicate significance (p = 0.0004). (D) The three sites within the PF11_0521 DBLβ3_D4-binding site, indicating residues that directly contact ICAM-1 (site 1, yellow; site 2, red; site 3, orange). Structural residues important for positioning of interacting residues are highlighted in teal. (E) Surface plasmon resonance curves for injection of 2-fold dilution series of DBLβ wild-type and binding site mutants over ICAM-1^D1D5^. (F) ICAM-1 binding (ELISA OD 490 nm; ± SD three replicates) of 26 recombinant group A DBLβ domains (4 DBLβ1, 14 DBLβ3, 2 DBLβ6, 2 DBLβ7, 2 DBLβ11, 1 DBLβ12, and 1 DBLβ unknown sub-class), confirming prediction of binding domains. “DC” indicates in which domain cassette the domain is found. “ND” indicates that the DC type is unknown as only the DBLβ sequence is available. “No” indicates that the domain is not part of a known DC. Presence of DC13 prior to DBLβ is indicated. (G and H) Sequence logo showing conservation of (G) residues that contact ICAM-1 and (H) residues important for the unusual architecture of the ICAM-1-binding site, based on 145 DBLβ domains predicted to bind ICAM-1. Red triangles, residues critical for direct interaction with ICAM-1. See also Figures S3–S5 and Tables S2, S3, and S4.

The PF11_0521 DBLβ3_D4 domain adopts the classical DBL domain fold, consisting of a core of α helices, decorated by numerous loops (Figures 1, S2A, and S2B). In contrast to previously characterized DBL domains, an extended α helix and a glycine and proline-rich linker form a unique feature that protrudes from the domain and generates part of the ICAM-1 interaction site (Figures 1, S2A, and S2B). Indeed, a chimeric protein containing this region of an ICAM-1-binding domain, transplanted to a related, non-binding DBLβ domain, produced an ICAM-1-binding domain (Figure 2A), and antibodies targeting this region (either purified from peptide-immunized rat or from human plasma) prevented infected erythrocytes from binding to ICAM-1 (Figures 2B and 2C).

The DBLβ domain interacts with ICAM-1 through a complex, elongated binding interface that contains three distinct subsites with a total surface area of 1,144 Å^2^ (Figure 1C). Site 1 is formed from a patch of hydrophobic residues along one side of the extended α helix of DBLβ that interacts with a corresponding hydrophobic patch on the surface of ICAM-1 D1. Site 2 is formed by the subsequent linker of the DBLβ domain and forms hydrogen bonds with both ICAM-1 D1 and D2 domains. Site 3 is formed from part of a long loop that protrudes from the DBLβ domain and also contacts both ICAM-1 D1 and D2. The parts of this loop not in contact with ICAM-1 are not observed in the electron density, indicating flexibility (Figures 1B and S2C). This loop is indeed predicted to be intrinsically disordered (Figure S2D), but becomes partially ordered when bound to ICAM-1. Such a flexible region of a protein, which forms part of, or is immediately adjacent to, its ligand-binding site, is an immune evasion strategy used by surface proteins from other pathogens (Kim et al., 2004, Kwong et al., 1998), but had not been previously observed in any PfEMP1 domain bound to its receptor.

### A Sequence Motif Allows for the Prediction of ICAM-1 Binding A-type PfEMP1

We next aimed to use the crystal structure to identify key molecular determinants used by the DBLβ domains for ICAM-1 binding, and to thereby identify a sequence motif that can be used to predict these ICAM-1 binders. For this, we mutated residues in the DBLβ domain that either directly contact ICAM-1 or are responsible for the unusual architecture of the binding site (Figure 2D; Table S2). By testing the eight residues that directly interact with ICAM-1 (Figure S3; Table S2), we found that each of the three subsites contains a critical residue that, when mutated, disrupts ICAM-1 binding (Figure 2E; Table S3). In addition, certain structural residues, in particular glycines and prolines, are essential for ICAM-1 binding as they allow sharp backbone bends that correctly present binding residues (Figures 2 and S3; Table S3). To ensure that the observed effects were not due to disruption in the overall fold of the DBL domain, all mutants that showed an effect on ICAM-1 binding were tested by circular dichroism spectroscopy and found to be indistinguishable from the wild-type domain (Figure S4).

**Figure 3:**
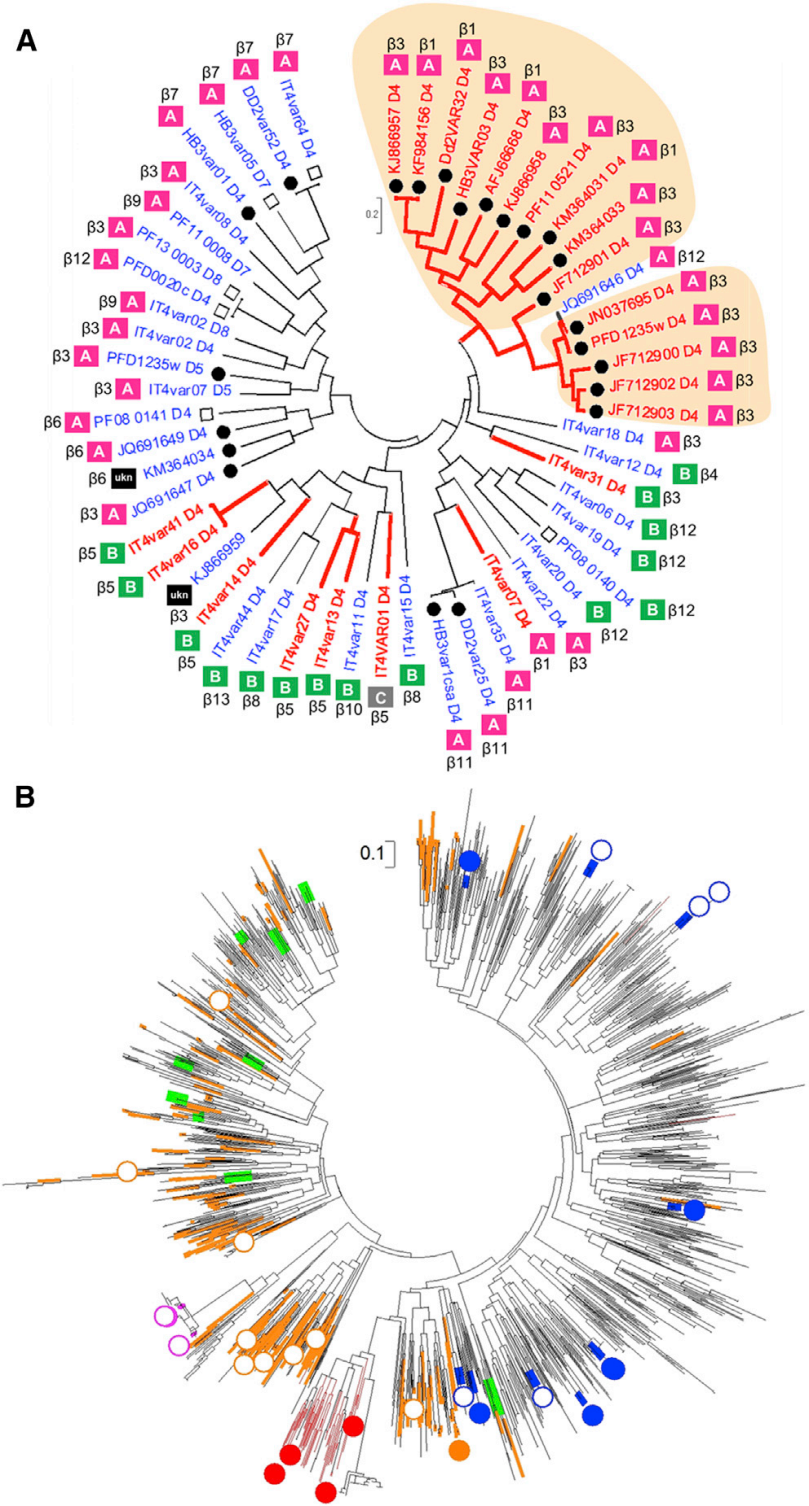
Phylogeny of ICAM-1- and Non-binding DBLβ Domains (A) Maximum likelihood tree of 55 complete DBLβ domains. The tree is drawn to scale, with branch lengths measured in substitutions per site. Red, ICAM-1 binders; blue, ICAM-1 non-binders (Avril et al., 2016, Bengtsson et al., 2013, Janes et al., 2011, Lau et al., 2015). Filled circles, DBLβ domains tested in this study; open squares, DBLβ domains tested, but data not shown. UKN indicates unknown group ID. Shaded areas indicate DBLβ with the ICAM-1 motif. (B) Maximum likelihood tree of 1,823 complete DBLβ S3 sequences from the seven published genomes and 226 annotated Sanger genomes. Filled circles, DBLβ experimentally shown as ICAM-1 binders. Open circles, DBLβ experimentally shown as ICAM-1 non-binders. Colors indicate the CIDR domain N-terminal to DBLβ: red, EPCR binders with ICAM-1-binding motif; orange, EPCR binders with no ICAM-1-binding motif; green, non-EPCR binders (group A); blue, CD36 binders; magenta, VAR1. See also Figure S5 and Table S4.

Based on this analysis, coupled with sequence analysis of previously identified A-type ICAM-1 binding domains (Bengtsson et al., 2013), we defined a sequence motif (I[V/L]x_3_N[E]GG[P/A]xYx_27_GPPx_3_H) that contains the determinants for ICAM-1 binding by these group A PfEMP1s. This motif was used to search sequence databases, identifying a total of 145 ICAM-1-binding DBL domains (mostly A-type PfEMP1s with a few B/A-type PfEMP1s) that contain the motif and are therefore predicted to bind to ICAM-1 (Table S4). To test these predictions, we used ELISA to analyze the interaction between ICAM-1 and 16 motif-containing DBLβ domains, selected to represent sequence diversity (Table S4). All 16 DBLβ domains with the motif bound ICAM-1, while 10 group A DBLβ domains without the motif did not (Figures 2F and 3A; Table S4). In sequence analysis of DBLβ domains (n = 55) with known ICAM-1-binding properties and DBLβ S3 sequences (n = 1,823) from whole-genome studies, the ICAM-1-binding group A and B/A domains with the motif clustered separately from other domains, including group B and C ICAM-1-binding DBLβ domains lacking the motif (Figures 3 and S5). Taken together, this suggests at least two groups of ICAM-1-binding PfEMP1s with different evolutionary histories and that the majority of ICAM-1-binding group A and B/A PfEMP1s can be predicted from the presence of a highly conserved binding site (Figures 2G and 2H).

### Parasites Expressing ICAM-1-Binding DBLβ Domains Elicit Inhibitory Antibodies and Are Associated with CM

**Figure 4:**
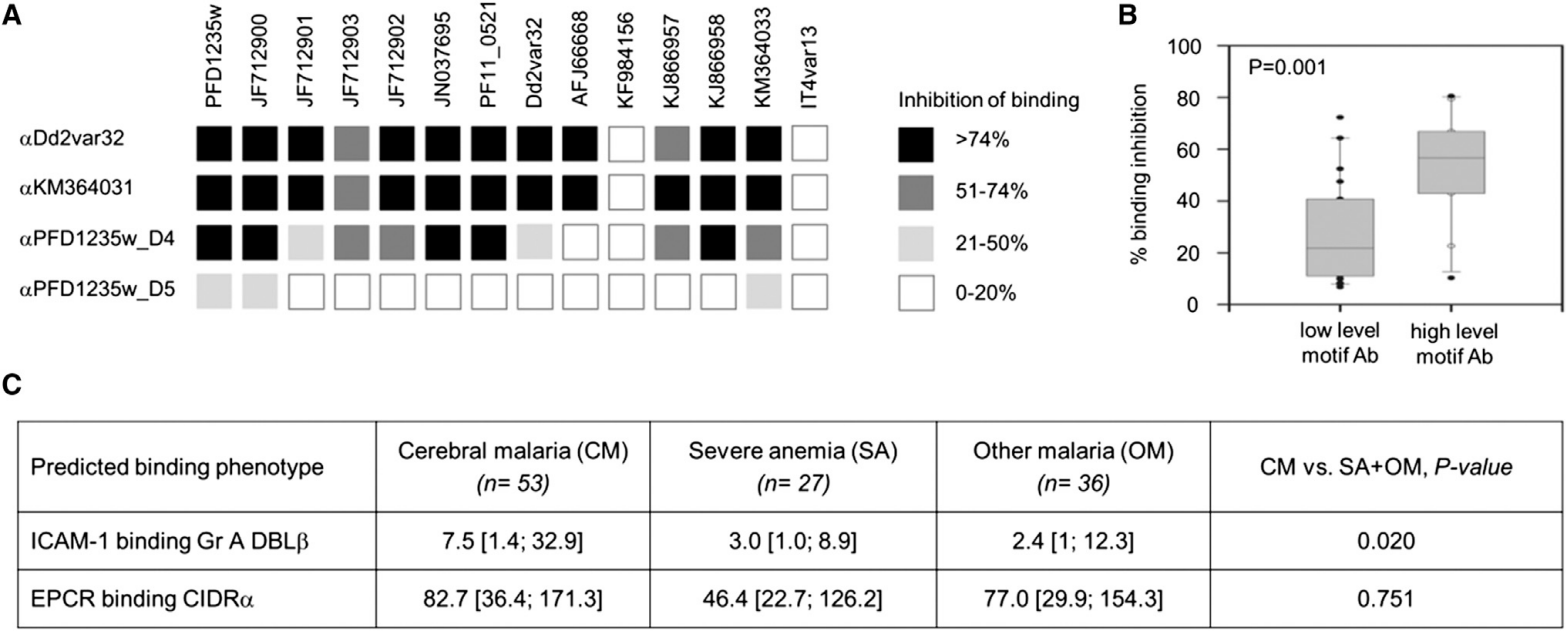
The Conserved ICAM-1 Binding Site Is Recognized by Patient Plasma and Linked to CM (A) The inhibition of DBLβ domains binding to ICAM-1 by IgG from a plasma pool from Liberian *P. falciparum*-exposed adults purified on ICAM-1-binding DBLβ_D4 domain from Dd2var32, KM364031, and PFD1235w or on a closely related, but non-ICAM-1-binding DBLβ_D5 domain from PFD1235w. ICAM-1 inhibitory capacity is >74% (black), 51%–74% (dark gray), 21%–50% (light gray), and 0%–20% (white). (B) ICAM-1-binding inhibition by plasma with low (ELISA OD < 1) and high (ELISA OD > 1) anti-motif-DBLβ IgG in *P. falciparum*-exposed Tanzanian children (1–17 years) (Lusingu et al., 2004). Boxplot with median. Whiskers, 5% and 95% percentiles. (C) Transcript levels (in arbitrary transcription units, Tu) of *var* gene subtypes in Ghanaian, Tanzanian, and Beninese children hospitalized with malaria, shown with medians and 25% and 75% percentiles. See also Table S5.

The high sequence identity of DBLβ domains containing the motif (Table S4) encouraged us to determine whether these domains might induce cross-reactive antibodies during natural infections. We therefore screened samples from Liberian adults for the presence of immune plasma that inhibited DBLβ domains from binding to ICAM-1. Plasma IgG from a pool of these inhibitory samples was then affinity purified separately on three different DBLβ domains containing the motif (Dd2var32, KM364031, and PFD1235w [PlasmoDB: PF3D7_0425800] DBLβ_D4) and tested for the ability to inhibit ICAM-1 binding of DBLβ domains (Figure 4A). The IgG showed the potential to broadly inhibit ICAM-1 binding by a panel of motif-containing DBLβ domains from group A PfEMP1, but did not prevent ICAM-1 binding by a DBLβ domain from a group B PfEMP1 (IT4VAR13). In contrast, IgG that was affinity purified on a closely related non-ICAM-1-binding DBLβ domain that lacks the motif (PFD1235w DBLβ_D5) did not significantly inhibit binding of any DBLβ domain tested (Figure 4A). Therefore, plasma from adults in a malaria endemic region contains IgG that shows the potential to broadly inhibit ICAM-1 binding of motif-containing PfEMP1.

We also tested whether children develop antibodies that specifically target the ICAM-1 binding site of the motif-containing DBLβ domains. To this end, we screened plasma samples from *Plasmodium falciparum*-exposed non-hospitalized Tanzanian children living in an area of high malaria transmission against a peptide containing the motif. A high proportion (69 of 76) of samples contained motif-reactive antibodies, and the DBLβ:ICAM-1-binding inhibition was significantly higher in plasma with high reactivity (ELISA optical density [OD] > 1) against the motif when compared with those with low motif reactivity (ELISA OD < 1) (Figure 4B).

Next, to correlate the expression of the group A ICAM-1-binding PfEMP1 with disease outcomes, we used the sequence motif to design primers to selectively amplify motif-encoding *var* gene transcripts. We used these to assess transcript levels in children with CM and severe malarial anemia (SA), and in other pediatric malaria patients (other malaria, OM; non-CM and non-SA) (Figure 4C; Table S5). The abundance of *var* gene transcripts was calculated relative to the transcript of an endogenous control gene and was translated into transcript units (Lavstsen et al., 2012). This analysis showed higher transcription of motif-encoding, ICAM-1-binding PfEMP1 in children with CM than in children with SA or in those admitted to hospital with malaria without these signs of severity (OM). Due to the challenges in acquiring a sufficiently large number of samples from single locations, patients were recruited at different sites and logistic regression modeling was used to correct for this. Comparison of the three groups showed that levels of transcripts encoding ICAM-1-binding PfEMP1 were significantly associated with risk of CM (p = 0.02) when comparing CM patients to malaria patients without CM (SA and OM). In contrast, transcript levels reported by primers targeting loci encoding EPCR-binding PfEMP1 (Mkumbaye et al., 2017) was not higher in CM patients compared to SA plus OM patients (p = 0.751). These data suggest a direct link between CM and expression of this particular subset of ICAM-1-binding PfEMP1s (Figure 4C).

### Motif-Containing ICAM-1-Binding DBLβ Domains Are Associated with EPCR-Binding Domains, Allowing Simultaneous Dual Receptor Binding

The correlation between CM and expression of group A PfEMP1 containing the conserved ICAM-1-binding site raised the question of why ICAM-1 binding was not unambiguously associated with CM in previous studies. With ICAM-1 binding alone unlikely to be the driver of cerebral disease, we therefore examined the broader context of the motif-containing ICAM-1-binding domains. We made the striking observation that all tested PfEMP1s containing these domains also contain a neighboring EPCR-binding CIDRα1 domain (Table S4; Figures 3B and S5). These PfEMP1s form a separate cluster from PfEMP1s that contain an EPCR-binding domain, but no motif containing ICAM-1-binding domain (Figures 3B and S5), and we find that 14% of all EPCR binders have such a motif-containing ICAM-1-binding domain. In contrast to this, B- and C-type ICAM-1-binding PfEMP1s are frequently associated with CIDRα domains that interact with CD36 and are distributed across the phylogenetic tree (Figure 3B; Table S4). This suggests that the group A ICAM-1-binding PfEMP1s are a subset of the ICAM-1-binding PfEMP1s, separate from group B and C PfEMP1s, and that they also have the capacity to bind to EPCR.

**Figure 5:**
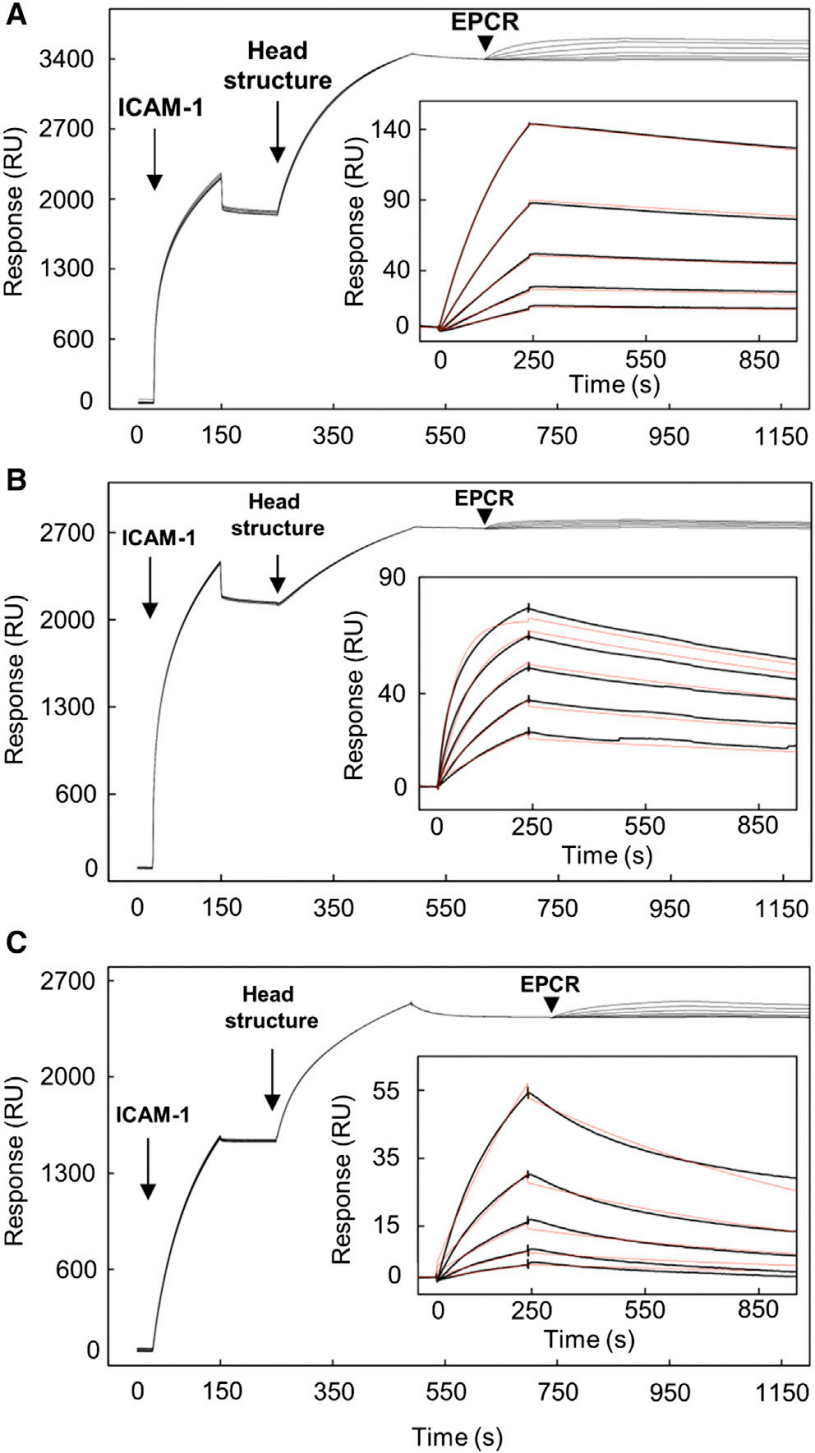
PfEMP1 Can Simultaneously Bind to Both ICAM-1 and EPCR ICAM-1^D1D5^ was immobilized at fixed concentration, followed by injection of the respective head structure, (A) PF11_0521 DBLα1.7-CIDRα1.4-DBLβ3, (B) Dd2var32 DBLα1.7-CIDRα1.4-DBLβ3, and (C) PFD1235w CIDRα1.6-DBLβ3, also at fixed concentration. A 2-fold dilution series of EPCR was injected over the resulting ICAM-1::head structure complex. Arrows show start points of protein injection. The insets show data fitted to a one-site kinetic model for binding of EPCR to the respective ICAM-1::head structure complex. Data (black lines) are modeled to a one-site model (red lines). See also Figure S6 and Table S6.

The link between the presence of both ICAM-1- and EPCR-binding domains within a single PfEMP1 raised the intriguing possibility that these PfEMP1s might simultaneously engage both receptors. A few PfEMP1s have previously been identified that contain both EPCR-binding CIDRα domains and ICAM-1-binding DBLβ domains (Avril et al., 2016, Oleinikov et al., 2009, Turner et al., 2013). However, it was unknown whether they could interact with both receptors simultaneously, and whether such dual binding would have consequences for infected erythrocyte adhesion. We therefore produced a set of three different recombinant PfEMP1s and used surface plasmon resonance to test their potential for binding to the two receptors simultaneously. Indeed, we found that protein constructs containing the ICAM-1- and EPCR-binding domains of these PfEMP1s all bound simultaneously to ICAM-1 and EPCR with nanomolar affinity (Figures 5 and S6; Table S6).

**Figure 6:**
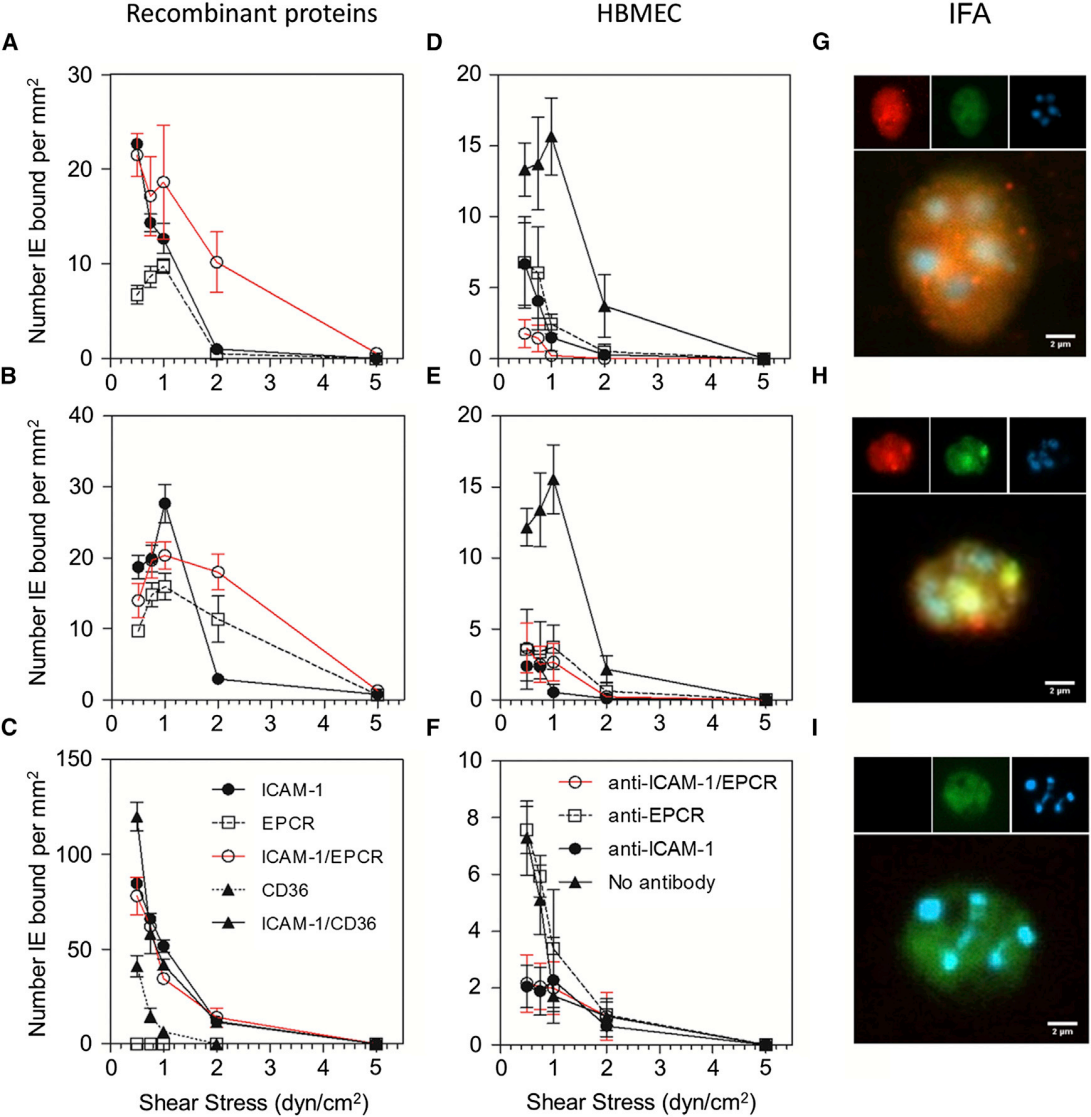
Binding to ICAM-1 and EPCR Increases Adhesion of Infected Erythrocytes (A–F) Erythrocytes infected with (A and D) HB3VAR03 (predicted ICAM-1 and EPCR binder), (B and E) 3D7PFD1235w (predicted ICAM-1 and EPCR binder), and (C and F) IT4VAR13 (ICAM-1 and CD36 binder) binding to (A–C) receptor-coated microslides under flow conditions using recombinant ICAM-1, rEPCR, rICAM-1, and rEPCR; rCD36; or rICAM-1 and rCD36, and to (D–F) resting human brain microvascular endothelial cells (HBMEC). To demonstrate specific adhesion, channels coated with HBMEC were pre-incubated with anti-ICAM-1 (40 μg/mL), anti-EPCR (10 μg/mL), or anti-ICAM1 and EPCR combined (40 and 10 μg/mL, respectively). Flow experiments were done in parallel with the same conditions for immobilizing proteins or cell-coated chips. Values are bound infected erythrocyte per mm^2^ ± SEM. Data were also analyzed by two-way ANOVA with Tukey’s multiple comparison (Tables S7A and S7B). (G–I) Immunofluorescence images of erythrocytes infected with parasite strains (G) HB3VAR03, (H) 3D7PFD1235w, and (I) IT4VAR13. Complexes of ICAM-1 or EPCR were added to measure binding by immunofluorescence. Main panels show overlays of ICAM-1 (green), EPCR (red), and nuclear (blue) staining. Inserts show single channels. Scale bar, 2 μm. See also Figure S7 and Table S7.

Next, we examined the effect of dual binding to ICAM-1 and EPCR in the context of infected erythrocytes. For this, erythrocytes infected with parasites expressing PfEMP1 (PFD1235w or HB3VAR03) predicted to bind both receptors were selected in vitro (Figure S7) and binding to both receptors was tested under flow conditions using recombinant receptors (Figures 6A–6C) and in an immunofluorescence assay (Figures 6G–6I). These studies were performed at a range of flow rates from 0.5 to 5.0 dyn/cm^2^, chosen to induce the most physiologically relevant shear stresses. Infected erythrocytes were capable of attaching between 0.5 and 1.0 dyn/cm^2^ in the presence of either recombinant receptor alone. When ICAM-1 and EPCR were simultaneously available at comparable molar concentrations, erythrocytes infected with parasites expressing dual-binding PfEMP1 demonstrated an increased ability to bind at a higher shear stress, i.e., 2 dyn/cm^2^, compared to when either receptor was available alone, though this adhesion did not exceed that measured at 1 dyn/cm^2^ (Figures 6A and 6B; Table S7A). In contrast, erythrocytes infected with parasites known to bind ICAM-1 and CD36 (IT4VAR13; Figures S7E and S7F) showed increased adhesion in the presence of both ICAM-1 and CD36 compared to CD36 binding alone at lower shear stress, but did not confer the ability to withstand higher shear stresses, and the adhesion decreased in response to increasing shear stress (Figure 6C). The final parasite, known to bind to PECAM-1 (PF11_0008 [PlasmoDB: PF3D7_1100200]; Figures S7G and S7H), failed to bind any of the receptors tested (Figure S7L).

To further test the physiological relevance of our observations, we assessed the binding of infected erythrocytes to HBMEC with and without tumor necrosis factor α (TNFα) treatment (Figures 6D–6F and S7I–S7K). To determine the contribution of individual receptors to binding, antibodies against either ICAM-1 or EPCR alone or a combination of both were used. With HB3VAR03, at 0.5 dyn/cm^2^ the greatest inhibition was observed on cells treated with both anti-receptor antibodies simultaneously (80%, p = 0.0361; Figure 6D), while HBMEC treated with either anti-ICAM-1 or anti-EPCR antibodies demonstrated a non-significant reduction in adhesion (Table S7B). A similar effect was observed at 0.75 dyn/cm^2^. With increasing shear stress, the activity of single-antibody treatment was enhanced, and at 1 dyn/cm^2^ this adhesion inhibition was significant for each individual anti-receptor antibody (Figure 6D; Table S7B), showing that the ability of HB3VAR03 to bind to both receptors is important at higher shear stresses. In comparison, 3D7PFD1235w infected erythrocytes were significantly inhibited by the presence of either antibody at all shear stresses tested (Figure 6E; Table S7B). As expected, IT4VAR13 only bound ICAM-1 and not EPCR expressed on the surface of HBMEC (Figure 6F). When the same experiments were conducted on activated cells, all three parasite lines showed increased reliance on ICAM-1 for adhesion, with HB3VAR03- and 3D7PFD1235w-infected erythrocytes switching to a single-receptor phenotype due to downregulation of EPCR expression upon cell activation by TNFα (Menschikowski et al., 2009) (Figures S7I–S7K). In summary, the ability to bind to both ICAM-1 and EPCR gives parasites significantly greater versatility, allowing for an increased capacity to adhere at physiological higher shear stresses, and enables them to continue to adhere despite changes in receptor expression upon cell activation.

### Association of ICAM-1 and EPCR Dual-Binding PfEMP1 with the Development of CM

**Figure 7:**
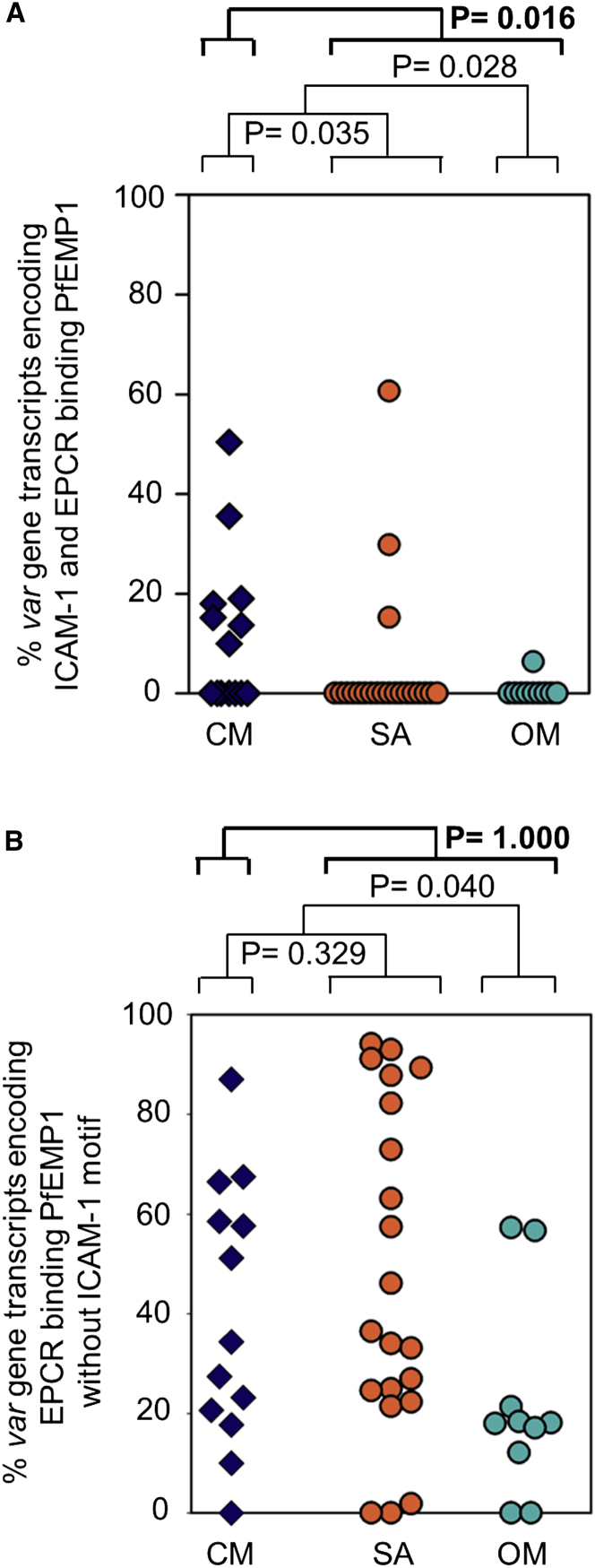
Dual-Binding PfEMP1s Correlate with CM Percentages of specific *var* gene transcripts in infected erythrocytes from 45 hospitalized Tanzanian children. (A) Percentage of *var* gene transcripts encoding PfEMP1s predicted to bind both ICAM-1 and EPCR. (B) Percentage of transcripts that encode PfEMP1 predicted to bind EPCR and not containing the ICAM-1-binding motif. CM, cerebral malaria; SA, severe anemia; OM, children without the signs of severity found in the other groups. See also Table S5.

Our ability to predict dual-binding PfEMP1 from sequence next allowed us to assess the link between the presence of parasites expressing such PfEMP1 and the development of different severe malaria syndromes in patient isolates, and to compare this with the disease outcomes associated with PfEMP1s that bind EPCR alone. For this, we analyzed a dataset containing the full sequences of the six most abundant PfEMP1 transcripts expressed in 45 children hospitalized with malaria (on average corresponding to 75% of total *var* transcripts; see Supplemental Experimental Procedures), the biggest available dataset of its kind (Jespersen et al., 2016). These children presented with CM or SA, or were admitted to hospital with malaria, but without these signs of severity, and were immediately treated with antimalarial drugs (Table S5). We identified dual-binding PfEMP1 from the sequence data by presence of a DBLβ domain containing the group A ICAM-1-binding motif adjacent to an EPCR-binding CIDRα1 domain. EPCR-binding PfEMP1s were identified by the presence of CIDRα1 domains alone. In this dataset, parasites expressing PfEMP1 predicted to bind EPCR, but not ICAM-1, were more likely to be found in severe malaria cases (CM plus SA) (p = 0.040) (Figure 7B). However, there was no association between EPCR binding and either of the specific severe malaria syndromes (with p = 1.000 for comparison of CM with all other types of malaria). In contrast, parasites expressing dual ICAM-1- and EPCR-binding PfEMP1 were specifically (p = 0.016) associated with CM, when compared with non-cerebral disease (SA plus OM) (Figure 7A). These data support our finding that the expression of ICAM-1-binding DBLβ domains containing the motif, which to date has been 100% associated with dual ICAM-1- and EPCR-binding PfEMP1 (Figures 3 and S5; Table S4), is associated with the development of cerebral symptoms (Figures 4C and 7). Therefore, while expression of PfEMP1s that bind to EPCR alone is not linked to any specific severe malaria syndrome, the presence of dual-binding motif-containing PfEMP1s is associated with the development of CM.

## Discussion

A major challenge in associating malaria disease phenotypes to the expression of PfEMP1s with specific binding properties has been the ability to predict ligand-binding capability from sequence alone. In this study, we used structural studies to define a sequence motif that allowed the identification of a large set of ICAM-1-binding group A PfEMP1s from sequence. We also show that these ICAM-1-binding PfEMP1s all contain an additional EPCR-binding CIDRα1 domain and that this allows binding to both ligands. Finally, we show that the presence of parasites expressing PfEMP1s that bind to both ICAM-1 and EPCR is associated with increased risk of developing cerebral symptoms to *P. falciparum* infections.

This link between dual binding and CM is supported by two independent studies (Figures 4C and 7). However, while significant, it is not absolute. This may partly be because the data show a snapshot of the parasites present in the peripheral blood of the children at the time of hospital admission, and the children involved were treated with antimalarial medication immediately upon diagnosis, preventing further development of the disease. For example, in the analysis of whole PfEMP1 sequences (Figure 7), we cannot exclude the possibility that the small number of patients (3/21) expressing dual-binding PfEMP1 and presenting with SA, but not CM, may have developed cerebral symptoms if left untreated, causing the association detected here to be an underestimate. There are also risks in overestimating the link between CM and expression of this group of PfEMP1s due to limitations in the sample size available for each of these studies. However, this is mitigated by the fact that we observe the same association between dual-binding PfEMP1s and CM in two independent studies with samples from different patient populations from three African countries (Figures 4C and 7), suggesting that the presence of these PfEMP1s on the infected erythrocyte surface is a risk factor for the development of CM.

There are several possible reasons why dual-binding PfEMP1s might contribute to the development of CM. One consequence of dual binding is increased endothelial adhesion under conditions of high shear stress (Figure 6). CM is associated with increased sequestered parasite biomass throughout the circulatory system, and this appears to have disproportionate effects on the regulation of the homeostasis of the brain, making increased adhesion a risk factor (Cunnington et al., 2013, Moxon et al., 2009, Storm and Craig, 2014). In addition to these general effects, specific consequences for the brain may occur due to modulation of signaling mediated by ICAM-1 and EPCR, as EPCR-mediated signaling and ICAM-1 expression are linked by a complex interplay that might also contribute to cerebral disease. Infected erythrocytes prevent EPCR from interacting with its natural ligands, inhibiting signaling pathways that induce anti-inflammatory responses and protect the integrity of the endothelium (Petersen et al., 2015, Turner et al., 2013). Indeed, inflammation and brain swelling both occur in CM and are important in disease development. In addition, blockage of EPCR signaling increases expression of several PfEMP1 adhesion receptors, including ICAM-1 (Moxon et al., 2013). Paradoxically, parasite sequestration in the brain is also linked to loss of EPCR, removing an important regulator of pro-coagulation responses (Moxon et al., 2013). The capacity to bind to both receptors may give the parasite more potential to resist endothelial changes in response to binding (i.e., downregulation of EPCR) and to remain sequestered in the brain. Together with the increased expression of ICAM-1 on endothelial cells, this could lead to a vicious cycle that further exacerbates disease.

CM is a complex disease, and in addition to dual-binding PfEMP1, various other aspects of both host and parasite biology will influence the development of cerebral symptoms. Other surface protein families, including STEVORs, RIFINs, and the PfEMP1 involved in rosetting (the clustering of infected and uninfected erythrocytes), have been implicated in severe malaria (Doumbo et al., 2009, Goel et al., 2015, Niang et al., 2014, Rowe et al., 1997) and further study is required to assess whether they too have a significant impact on the development of cerebral symptoms. In addition, host genetic factors, such as erythrocyte surface protein polymorphisms, could affect the capacity of the parasite to invade erythrocytes, altering parasitemia and therefore severity (Band et al., 2015). Nevertheless, the identification of dual-binding PfEMP1 as a risk factor for CM highlights these molecules as a promising and tractable candidate for the development of a vaccine to contribute to the prevention of this form of the disease.

A major challenge in targeting PfEMP1s through vaccination is their high sequence variability, driven by a diversifying selection pressure to evade immune detection. This is demonstrated by structural studies of CIDRα domains bound to EPCR or CD36 that revealed their interaction sites to retain their overall chemical nature, while significantly diversifying in sequence (Hsieh et al., 2016, Lau et al., 2015). This reduces the likelihood of the natural development of cross-inhibitory antibody responses that target these critical surfaces of CIDR domains. In contrast, the ICAM-1-binding site of the group A PfEMP1 is remarkably conserved, with a number of absolutely or predominantly conserved amino acid residues that make critical, direct interactions with ICAM-1 (Figure 2G). Indeed, we find that plasma from *P. falciparum*-exposed individuals contains IgG with the capacity to inhibit ICAM-1 binding by a broad range of motif-containing DBLβ domains (Figure 4). This encourages future efforts to raise broadly reactive antibody responses against these domains. This study therefore highlights stronger endothelial adhesion capacity to both ICAM-1 and EPCR as a risk factor for development of CM and identifies a group of PfEMP1s with a remarkably conserved ICAM-1-binding site as potential vaccine immunogen for use as part of a strategy to prevent death due to CM.

## Experimental Procedures

More detailed methods and references are provided in the Supplemental Experimental Procedures.

### Crystal Structure Determination

The PF11_0521 DBLβ3_D4 domain was produced as described in the Supplemental Experimental Procedures and was mixed with a 1.5 molar excess of ICAM-1^D1D2^ before size exclusion chromatography. Crystals were grown using sitting-drop vapor diffusion with a well solution of 10% (w/v) PEG 20000, 20% (v/v) PEG 500, and 0.1 M Tris-BICINE (pH 8.5) and cryo-cooled in well solution with an increased PEG 500 concentration of 32%.

Data were collected on beamline I02 (Diamond Light Source), indexed and refined using iMosflm, and scaled using SCALA (Evans and Murshudov, 2013). Molecular replacement was performed using Phaser-MR (McCoy et al., 2007) with a trimmed DBLα domain (PDB: 2XU0) and individual D1 and D2 domains from ICAM-1^D1D2^ (PDB: 1IC1) as search models. This identified one copy of the complex in the asymmetric unit. The structure was built and refined through iterative cycles of refinement using Refmac5 (Murshudov et al., 2011), Buster (Bricogne et al., 2016), and model building in Coot (Emsley et al., 2010).

### Surface Plasmon Resonance

Surface plasmon resonance experiments were carried out in a Biacore T200 instrument (GE Healthcare). ICAM-1^D1D5^-Fc was bound to a CM5 chip (GE Healthcare) pre-coupled to protein A while EPCR was biotinylated by BirA and coupled to a biotin CAPture chip (GE Healthcare). Binding partners were injected for 240 s with a dissociation time of 660 s. To analyze dual binding to ICAM-1 and EPCR, ICAM-1^D1D5^-Fc was immobilized. Two- and three-domain PfEMP1 constructs were then injected, followed by a concentration series of EPCR. Kinetic sensorgrams were fitted to a 1:1 interaction model to determine kinetic rate constants and dissociation constant.

### Small-Angle X-Ray Scattering

Small-angle X-ray scattering data were collected on beamline BM29 at the European Synchrotron Radiation Facility and processed using the ATSAS processing suite. The resulting ab initio model was converted into an envelope before manual model docking.

### Patient Samples

*Plasmodium falciparum* isolates were collected from malaria patients at hospitals in Ghana, Tanzania, and Benin. Clinical manifestations of malaria were classified according to the definitions and associated criteria of the World Health Organization. CM was identified by a positive blood smear of the asexual form of *P. falciparum* and unrousable coma (Blantyre coma score, BCS ≤ 2) with exclusion of other causes of coma and severe illness. SA was identified by hemoglobin < 5g/dL and BCS > 2. OM was indicated by BCS > 2 and hemoglobin > 5 g/dL. These studies were approved by the respective ethics committees. Additional plasma samples were collected from *P. falciparum*-exposed children during a cross-sectional village study in Tanzania and from *falciparum*-exposed adult Liberians (Lusingu et al., 2004, Theisen et al., 2000).

### Antibody Production and Purification

Rat antibodies were obtained by immunization with DBLβ domains, followed by affinity purification of IgG. Human antibodies against DBLβ domains or the ICAM-1-binding motif were affinity purified from pooled plasma from nine Liberian adults.

### ELISA

ICAM-1 binding was assessed by ELISA using DBLβ domain-coated Maxisorp plates, with detection using ICAM-1^D1D5^-Fc and rabbit anti-human IgG-HRP (Dako). A similar assay was used to assess the reactivity of human plasma samples to the DBLβ domains or to assess their efficacy at blocking ICAM-1 binding.

### Assessment of Infected Erythrocyte Adhesion to Receptors

*Plasmodium falciparum* parasites were maintained in in vitro blood culture and selected for specific PfEMP1 expression using antibodies. Microslides (Ibidi) were coated with recombinant receptors (ICAM-1^D1D5^-Fc, EPCR, or CD36) or seeded with HBMEC (Sciencell). Infected erythrocytes were exposed to the microslides at a range of physiological shear stresses and bound infected erythrocytes were quantified by microscopy.

### Immunofluorescence

Infected erythrocytes were incubated with complexes of ICAM-1^D1D5^-Fc:anti-human IgG-Dylight 488 (Abcam) and/or EPCR:anti-his IgG (QIAGEN):anti-mouse IgG Alexa 568 (Abcam) before washing and analysis in a Zeiss AxioImager Z1.

### Real-Time qPCR

Parasite RNA from clinical samples was reverse transcribed into DNA and subjected to real-time qPCR using primer pairs (Table S7C) designed to amplify ICAM-1 motif-containing *var* genes or *var* gene subclasses encoding EPCR-binding CIDRα1 domains.

### Sequence Analysis

DBLβ domain sequences (Rask et al., 2010) were used to blast extract DBLβ sequences from assemblies of Illumina whole-genome sequencing data, including MalariaGEN data (Miotto et al., 2015) and assembled amplicons from eight Ghanaian isolates. Those predicted to bind ICAM-1 were identified using search term ASNGGPGYYNTEVQK. A database containing sequences of the six most expressed *var* genes in 45 Tanzanian children with severe or uncomplicated malaria (Jespersen et al., 2016) was used to correlate relative expression of motif-containing *var* genes when compared with disease outcome. *Var* gene expression and disease severity were analyzed using the Wilcoxon-Mann-Whitney rank-sum test. To determine whether study site affected the outcome, a logistical regression model was used.

## Author Contributions

F.L., Y.A., A.B., T.L., M.K.H., and A.T.R.J. conceived and planned the study. F.L., Y.A., L.H., M.K.H., and A.T.R.J. wrote the manuscript. F.L., A.B., R.W.O., L.T., B.G., S.N.-S., and A.T.R.J. contributed with recombinant proteins and antibodies. F.L. purified and crystallized proteins and collected and analyzed small-angle X-ray scattering data. F.L. and M.K.H. prepared crystals, collected data, and solved the structure. F.L. and C.K.Y.L. performed and analyzed surface plasmon resonance experiments. Y.A. performed adhesion assays and IFA. A.B. and J.E.V.P. did parasite culturing and flow cytometry analysis. R.W.O. and G. E.-M. performed ELISA studies. R.W.O. and A.T.R.J. did real-time qPCR work. J.S.J., T.L., and A.T.R.J. did the sequencing and bioinformatics analysis. N.T.N., G.E.-M., A.M., M.F.O., J.P.L., C.W.W., and T.G.T. organized clinical work and processed clinical samples. All authors read and commented on the manuscript. F.L., Y.A., A.B., M.K.H., and A.T.R.J. contributed equally to the work.

## Accession Numbers

Sequences originating from the present study are deposited in GenBank with accession numbers GenBank: KF984156, KJ866957, KJ866958, KJ866959, KM364031, KM364032, KM364033, and KM364034. Structure files have been deposited in the PDB under ID code PDB: 5MZA.

## Supplemental Information

Document S1. Supplemental Experimental Procedures, Figures S1–S7, and Tables S1–S3, S5, and S6

Table S4. The ICAM-1-Binding Motif Is Commonly Found in *P. falciparum* Genomes, Related to Figures 2 and 3

Table S7. Statistical Analysis of the Effect of Receptor and Shear Stress on Adhesion of HB3VAR03-, 3D7*PFD1235w*-, and IT4VAR13-Infected Erythrocytes under Physiological Flow Conditions and Primers Used for Amplification of PfEMP1-Specific DNA Sequences and Recombinant Proteins Produced, Related to Figures 1, 2, 3, 4, 5, and 6

Document S2. Article plus Supplemental Information

## References

[bib1] Avril M., Tripathi A.K., Brazier A.J., Andisi C., Janes J.H., Soma V.L., Sullivan D.J., Bull P.C., Stins M.F., Smith J.D. (2012). A restricted subset of var genes mediates adherence of Plasmodium falciparum-infected erythrocytes to brain endothelial cells. Proc. Natl. Acad. Sci. USA.

[bib2] Avril M., Bernabeu M., Benjamin M., Brazier A.J., Smith J.D. (2016). Interaction between endothelial protein C receptor and intercellular adhesion molecule 1 to mediate binding of Plasmodium falciparum-infected erythrocytes to endothelial cells. MBio.

[bib3] Band G., Rockett K.A., Spencer C.C., Kwiatkowski D.P., Malaria Genomic Epidemiology Network (2015). A novel locus of resistance to severe malaria in a region of ancient balancing selection. Nature.

[bib4] Bengtsson A., Joergensen L., Rask T.S., Olsen R.W., Andersen M.A., Turner L., Theander T.G., Hviid L., Higgins M.K., Craig A. (2013). A novel domain cassette identifies Plasmodium falciparum PfEMP1 proteins binding ICAM-1 and is a target of cross-reactive, adhesion-inhibitory antibodies. J. Immunol..

[bib5] Bernabeu M., Danziger S.A., Avril M., Vaz M., Babar P.H., Brazier A.J., Herricks T., Maki J.N., Pereira L., Mascarenhas A. (2016). Severe adult malaria is associated with specific PfEMP1 adhesion types and high parasite biomass. Proc. Natl. Acad. Sci. USA.

[bib6] Birbeck G.L., Molyneux M.E., Kaplan P.W., Seydel K.B., Chimalizeni Y.F., Kawaza K., Taylor T.E. (2010). Blantyre Malaria Project Epilepsy Study (BMPES) of neurological outcomes in retinopathy-positive paediatric cerebral malaria survivors: a prospective cohort study. Lancet Neurol..

[bib7] Bricogne, G., Blanc, E., Brandl, M., Flensburg, C., Keller, P., Paciorek, W., Roversi, P., Sharff, A., Smart, O.S., Vonrhein, C., and Womack, T.O. (2016). BUSTER version 2.10.2 (Global Phasing).

[bib8] Claessens A., Adams Y., Ghumra A., Lindergard G., Buchan C.C., Andisi C., Bull P.C., Mok S., Gupta A.P., Wang C.W. (2012). A subset of group A-like var genes encodes the malaria parasite ligands for binding to human brain endothelial cells. Proc. Natl. Acad. Sci. USA.

[bib9] Cunnington A.J., Riley E.M., Walther M. (2013). Stuck in a rut? Reconsidering the role of parasite sequestration in severe malaria syndromes. Trends Parasitol..

[bib10] Dondorp A.M., Fanello C.I., Hendriksen I.C., Gomes E., Seni A., Chhaganlal K.D., Bojang K., Olaosebikan R., Anunobi N., Maitland K., AQUAMAT group (2010). Artesunate versus quinine in the treatment of severe falciparum malaria in African children (AQUAMAT): an open-label, randomised trial. Lancet.

[bib11] Doumbo O.K., Thera M.A., Koné A.K., Raza A., Tempest L.J., Lyke K.E., Plowe C.V., Rowe J.A. (2009). High levels of Plasmodium falciparum rosetting in all clinical forms of severe malaria in African children. Am. J. Trop. Med. Hyg..

[bib12] Emsley P., Lohkamp B., Scott W.G., Cowtan K. (2010). Features and development of Coot. Acta Crystallogr. D Biol. Crystallogr..

[bib13] Evans P.R., Murshudov G.N. (2013). How good are my data and what is the resolution?. Acta Crystallogr. D Biol. Crystallogr..

[bib14] Goel S., Palmkvist M., Moll K., Joannin N., Lara P., Akhouri R.R., Moradi N., Öjemalm K., Westman M., Angeletti D. (2015). RIFINs are adhesins implicated in severe Plasmodium falciparum malaria. Nat. Med..

[bib15] Howell D.P., Levin E.A., Springer A.L., Kraemer S.M., Phippard D.J., Schief W.R., Smith J.D. (2008). Mapping a common interaction site used by *Plasmodium falciparum* Duffy binding-like domains to bind diverse host receptors. Mol. Microbiol..

[bib16] Hsieh F.L., Turner L., Bolla J.R., Robinson C.V., Lavstsen T., Higgins M.K. (2016). The structural basis for CD36 binding by the malaria parasite. Nat. Commun..

[bib17] Hviid L., Jensen A.T. (2015). PfEMP1—a parasite protein family of key importance in *Plasmodium falciparum* malaria immunity and pathogenesis. Adv. Parasitol..

[bib18] Idro R., Jenkins N.E., Newton C.R. (2005). Pathogenesis, clinical features, and neurological outcome of cerebral malaria. Lancet Neurol..

[bib19] Janes J.H., Wang C.P., Levin-Edens E., Vigan-Womas I., Guillotte M., Melcher M., Mercereau-Puijalon O., Smith J.D. (2011). Investigating the host binding signature on the Plasmodium falciparum PfEMP1 protein family. PLoS Pathog..

[bib20] Jensen A.T., Magistrado P., Sharp S., Joergensen L., Lavstsen T., Chiucchiuini A., Salanti A., Vestergaard L.S., Lusingu J.P., Hermsen R. (2004). *Plasmodium falciparum* associated with severe childhood malaria preferentially expresses PfEMP1 encoded by group A *var* genes. J. Exp. Med..

[bib21] Jespersen J.S., Wang C.W., Mkumbaye S.I., Minja D.T., Petersen B., Turner L., Petersen J.E., Lusingu J.P., Theander T.G., Lavstsen T. (2016). Plasmodium falciparum var genes expressed in children with severe malaria encode CIDRα1 domains. EMBO Mol. Med..

[bib22] Kim J.H., Singvall J., Schwarz-Linek U., Johnson B.J., Potts J.R., Höök M. (2004). BBK32, a fibronectin binding MSCRAMM from Borrelia burgdorferi, contains a disordered region that undergoes a conformational change on ligand binding. J. Biol. Chem..

[bib23] Kwong P.D., Wyatt R., Robinson J., Sweet R.W., Sodroski J., Hendrickson W.A. (1998). Structure of an HIV gp120 envelope glycoprotein in complex with the CD4 receptor and a neutralizing human antibody. Nature.

[bib24] Lau C.K., Turner L., Jespersen J.S., Lowe E.D., Petersen B., Wang C.W., Petersen J.E., Lusingu J., Theander T.G., Lavstsen T., Higgins M.K. (2015). Structural conservation despite huge sequence diversity allows EPCR binding by the PfEMP1 family implicated in severe childhood malaria. Cell Host Microbe.

[bib25] Lavstsen T., Turner L., Saguti F., Magistrado P., Rask T.S., Jespersen J.S., Wang C.W., Berger S.S., Baraka V., Marquard A.M. (2012). Plasmodium falciparum erythrocyte membrane protein 1 domain cassettes 8 and 13 are associated with severe malaria in children. Proc. Natl. Acad. Sci. USA.

[bib26] Lusingu J.P., Vestergaard L.S., Mmbando B.P., Drakeley C.J., Jones C., Akida J., Savaeli Z.X., Kitua A.Y., Lemnge M.M., Theander T.G. (2004). Malaria morbidity and immunity among residents of villages with different Plasmodium falciparum transmission intensity in North-Eastern Tanzania. Malar. J..

[bib27] McCoy A.J., Grosse-Kunstleve R.W., Adams P.D., Winn M.D., Storoni L.C., Read R.J. (2007). Phaser crystallographic software. J. Appl. Cryst..

[bib28] Menschikowski M., Hagelgans A., Eisenhofer G., Siegert G. (2009). Regulation of endothelial protein C receptor shedding by cytokines is mediated through differential activation of MAP kinase signaling pathways. Exp. Cell Res..

[bib29] Miotto O., Amato R., Ashley E.A., MacInnis B., Almagro-Garcia J., Amaratunga C., Lim P., Mead D., Oyola S.O., Dhorda M. (2015). Genetic architecture of artemisinin-resistant Plasmodium falciparum. Nat. Genet..

[bib30] Mkumbaye S.I., Wang C.W., Lyimo E., Jespersen J.S., Manjurano A., Mosha J., Kavishe R.A., Mwakalinga S.B., Minja D.T., Lusingu J.P. (2017). The severity of Plasmodium falciparum infection is associated with transcript levels of var genes encoding EPCR-binding PfEMP1. Infect. Immun..

[bib31] Moxon C.A., Heyderman R.S., Wassmer S.C. (2009). Dysregulation of coagulation in cerebral malaria. Mol. Biochem. Parasitol..

[bib32] Moxon C.A., Wassmer S.C., Milner D.A., Chisala N.V., Taylor T.E., Seydel K.B., Molyneux M.E., Faragher B., Esmon C.T., Downey C. (2013). Loss of endothelial protein C receptors links coagulation and inflammation to parasite sequestration in cerebral malaria in African children. Blood.

[bib33] Murshudov G.N., Skubák P., Lebedev A.A., Pannu N.S., Steiner R.A., Nicholls R.A., Winn M.D., Long F., Vagin A.A. (2011). REFMAC5 for the refinement of macromolecular crystal structures. Acta Crystallogr. D Biol. Crystallogr..

[bib34] Newbold C., Warn P., Black G., Berendt A., Craig A., Snow B., Msobo M., Peshu N., Marsh K. (1997). Receptor-specific adhesion and clinical disease in Plasmodium falciparum. Am. J. Trop. Med. Hyg..

[bib35] Niang M., Bei A.K., Madnani K.G., Pelly S., Dankwa S., Kanjee U., Gunalan K., Amaladoss A., Yeo K.P., Bob N.S. (2014). STEVOR is a Plasmodium falciparum erythrocyte binding protein that mediates merozoite invasion and rosetting. Cell Host Microbe.

[bib36] Ochola L.B., Siddondo B.R., Ocholla H., Nkya S., Kimani E.N., Williams T.N., Makale J.O., Liljander A., Urban B.C., Bull P.C. (2011). Specific receptor usage in Plasmodium falciparum cytoadherence is associated with disease outcome. PLoS ONE.

[bib37] Oleinikov A.V., Amos E., Frye I.T., Rossnagle E., Mutabingwa T.K., Fried M., Duffy P.E. (2009). High throughput functional assays of the variant antigen PfEMP1 reveal a single domain in the 3D7 *Plasmodium falciparum* genome that binds ICAM1 with high affinity and is targeted by naturally acquired neutralizing antibodies. PLoS Pathog..

[bib38] Petersen J.E., Bouwens E.A., Tamayo I., Turner L., Wang C.W., Stins M., Theander T.G., Hermida J., Mosnier L.O., Lavstsen T. (2015). Protein C system defects inflicted by the malaria parasite protein PfEMP1 can be overcome by a soluble EPCR variant. Thromb. Haemost..

[bib39] Rask T.S., Hansen D.A., Theander T.G., Gorm Pedersen A., Lavstsen T. (2010). Plasmodium falciparum erythrocyte membrane protein 1 diversity in seven genomes—divide and conquer. PLoS Comput. Biol..

[bib40] Robinson B.A., Welch T.L., Smith J.D. (2003). Widespread functional specialization of *Plasmodium falciparum* erythrocyte membrane protein 1 family members to bind CD36 analysed across a parasite genome. Mol. Microbiol..

[bib41] Rogerson S.J., Tembenu R., Dobaño C., Plitt S., Taylor T.E., Molyneux M.E. (1999). Cytoadherence characteristics of Plasmodium falciparum-infected erythrocytes from Malawian children with severe and uncomplicated malaria. Am. J. Trop. Med. Hyg..

[bib42] Rowe J.A., Moulds J.M., Newbold C.I., Miller L.H. (1997). P. falciparum rosetting mediated by a parasite-variant erythrocyte membrane protein and complement-receptor 1. Nature.

[bib43] Seydel K.B., Kampondeni S.D., Valim C., Potchen M.J., Milner D.A., Muwalo F.W., Birbeck G.L., Bradley W.G., Fox L.L., Glover S.J. (2015). Brain swelling and death in children with cerebral malaria. N. Engl. J. Med..

[bib44] Silamut K., Phu N.H., Whitty C., Turner G.D., Louwrier K., Mai N.T., Simpson J.A., Hien T.T., White N.J. (1999). A quantitative analysis of the microvascular sequestration of malaria parasites in the human brain. Am. J. Pathol..

[bib45] Storm J., Craig A.G. (2014). Pathogenesis of cerebral malaria—inflammation and cytoadherence. Front. Cell. Infect. Microbiol..

[bib46] Theisen M., Soe S., Jessing S.G., Okkels L.M., Danielsen S., Oeuvray C., Druilhe P., Jepsen S. (2000). Identification of a major B-cell epitope of the Plasmodium falciparum glutamate-rich protein (GLURP), targeted by human antibodies mediating parasite killing. Vaccine.

[bib47] Turner G.D., Morrison H., Jones M., Davis T.M., Looareesuwan S., Buley I.D., Gatter K.C., Newbold C.I., Pukritayakamee S., Nagachinta B. (1994). An immunohistochemical study of the pathology of fatal malaria. Evidence for widespread endothelial activation and a potential role for intercellular adhesion molecule-1 in cerebral sequestration. Am. J. Pathol..

[bib48] Turner L., Lavstsen T., Berger S.S., Wang C.W., Petersen J.E., Avril M., Brazier A.J., Freeth J., Jespersen J.S., Nielsen M.A. (2013). Severe malaria is associated with parasite binding to endothelial protein C receptor. Nature.

