# Supplementary material for: Structure-Guided Identification of a Family of Dual Receptor-Binding PfEMP1 that Is Associated with Cerebral Malaria
Source: Cell Host Microbe. 2017 Mar 8;21(3):403–14. doi: 10.1016/j.chom.2017.02.009 (PMC5374107; doi:10.1016/j.chom.2017.02.009)
Supplement: Document S1. Supplemental Experimental Procedures, Figures S1–S7, and Tables S1–S3, S5, and S6 [file mmc1.pdf]

Cell Host & Microbe, Volume *21*

Supplemental Information

Structure-Guided Identification of a Family of Dual Receptor-Binding PfEMP1 that Is Associated with Cerebral Malaria

Frank Lennartz, Yvonne Adams, Anja Bengtsson, Rebecca W. Olsen, Louise Turner, Nicaise T. Ndam, Gertrude Ecklu-Mensah, Azizath Moussiliou, Michael F. Ofori, Benoit Gamain, John P. Lusingu, Jens E.V. Petersen, Christian W. Wang, Sofia Nunes-Silva, Jakob S. Jespersen, Clinton K.Y. Lau, Thor G. Theander, Thomas Lavstsen, Lars Hviid, Matthew K. Higgins, and Anja T.R. Jensen

Supplemental Figures

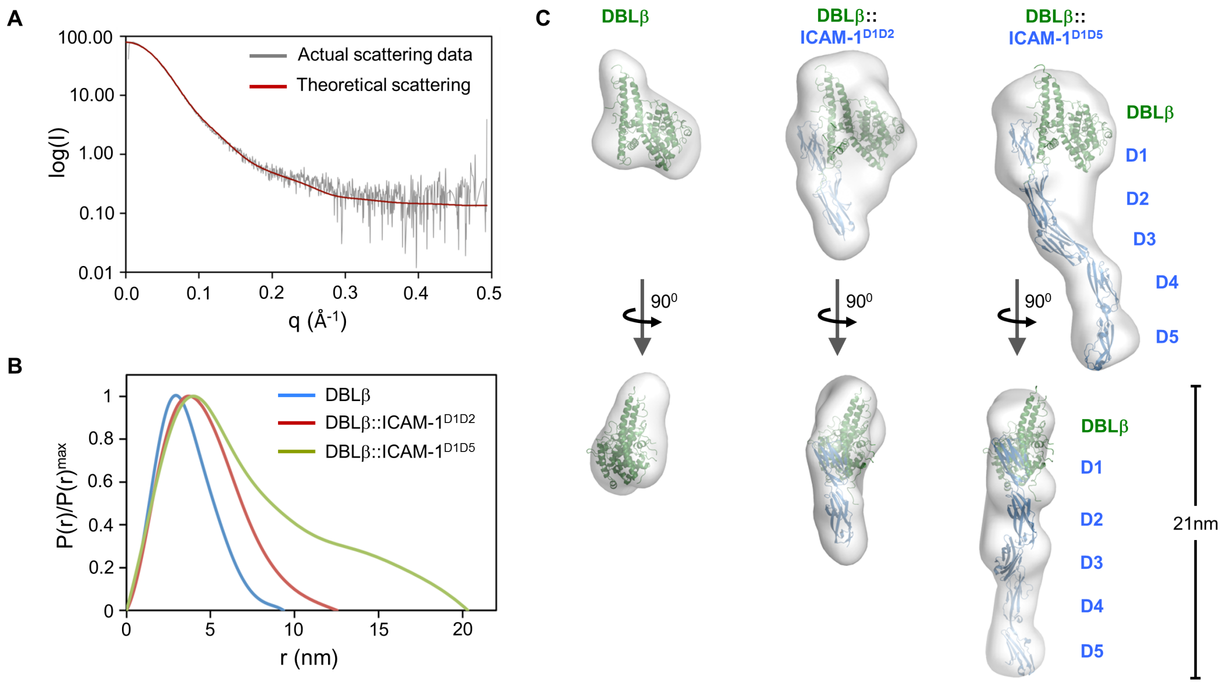

D

	$Rg_{exp.}$ (nm)	D_{max} (nm)	V_{porod} (nm^3)	Mr_{exp} (kDa)	Mr_{app} (kDa)	χ
DBLβ	2.72	9.35	88	55	51	1.006
DBLβ::ICAM-1^{D1D2}	3.58	12.53	140	77	82	1.185
DBLβ::ICAM-1^{D1D5}	5.80	20.31	237	131	139	1.209

Figure S1. SAXS analysis of the PF11_0521 DBLβ3_D4:ICAM-1 complex, ***related to Figure 1***

(A) Comparison between solution scattering data collected for the PF11_0521 DBLβ3_D4:ICAM-1^{D1D2} complex and the theoretical scattering calculated from the crystal structure of the same complex. The fit of experimental and theoretical scattering was calculated with Crysol, giving a χ^2 of 3.58.

(B) Normalized distance distribution function P(r) for PF11_0521 DBLβ3_D4 alone and bound to ICAM-1^{D1D2} or ICAM-1^{D1D5}.

(C) *Ab initio* envelopes calculated from SAXS data for the PF11_0521 DBLβ3_D4 domain alone and in complex with ICAM-1^{D1D2} or ICAM-1^{D1D5}. The structures of the PF11_0521 DBLβ3_D4 domain (from the co-crystal structure of PF11_0521 DBLβ3_D4:ICAM-1^{D1D2} complex), the PF11_0521 DBLβ3_D4: ICAM-1^{D1D2} complex and the structure of ICAM-1^{D3D5} (PDB 2OZ4) were manually docked into the envelopes.

(D) Parameters derived from solution scattering data. The radius of gyration R_g was determined using AutoRg, the maximum particle diameter D_{max} was calculated with GNOM. The Porod volume V_{porod} was calculated with PRIMUS. Mr_{exp} is the expected molecular mass, Mr_{app} is the mass calculated from the excluded volume in the final model derived from DAMMIN, divided by 2. χ is the best fit of the average from 20 low-resolution shape reconstructions to the experimental scattering data.

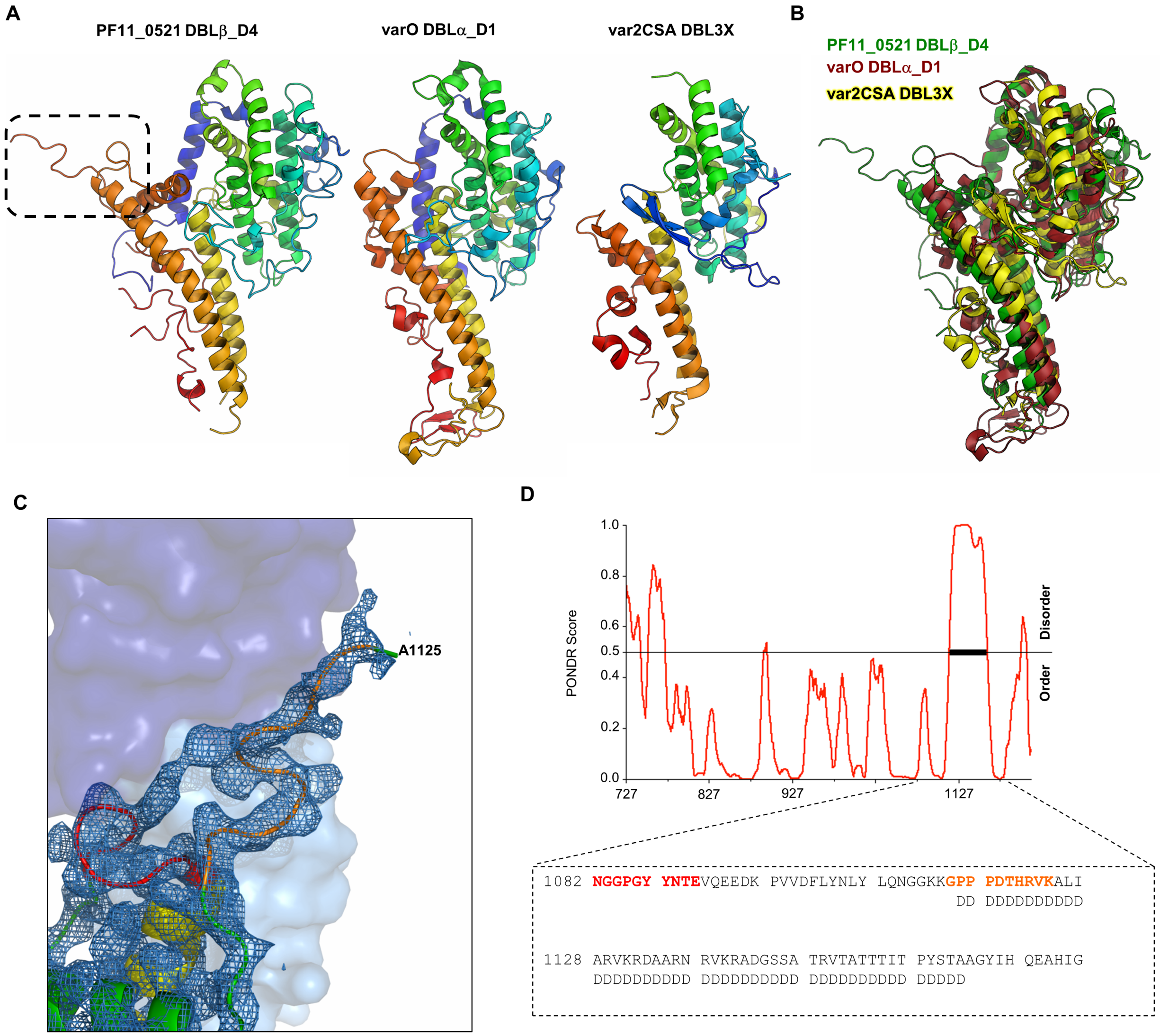

Figure S2. Unusual structural features of the ICAM-1 binding site in group A PfEMP1, *related to Figure 1*

(A) Comparison between known DBL domain structures. DBL domains from the PF11_0521, varO (PDB 2XU0) and vars2CSA (PDB 3BQI) PfEMP1 are shown in similar orientation for comparison and colored-coded from the N-terminus (blue) to C-terminus (red). The dashed box indicates the ICAM-1 binding site in the PF11_0521 DBLβ3_D4 domain.

(B) Superposition of the structures for the DBL domains of PF11_0521 (green), varO (red) and var2CSA (yellow). The superposition was prepared with PyMol (Schroedinger).

(C) Electron density map (2FoFc map) for residues in site 3 of the ICAM-1 binding site. The map is shown at a σ level of 1.0. Parts of the DBL domain and ICAM-1 are color coded according to Figure 1 of the main text. The last visible side chain of the loop that contains site 3 is indicated.

(D) Disordered regions in the PF11_0521 DBLβ3_D4 domain were predicted using PONDR (http://www.pondr.com/). A PONDR score of >0.5 indicates disorder. The inset shows the sequence of a potentially disordered region in the PF11_0521 DBLβ3_D4 domain. Residues in site 2 (red) and 3 (orange) of the ICAM-1 binding site are highlighted.

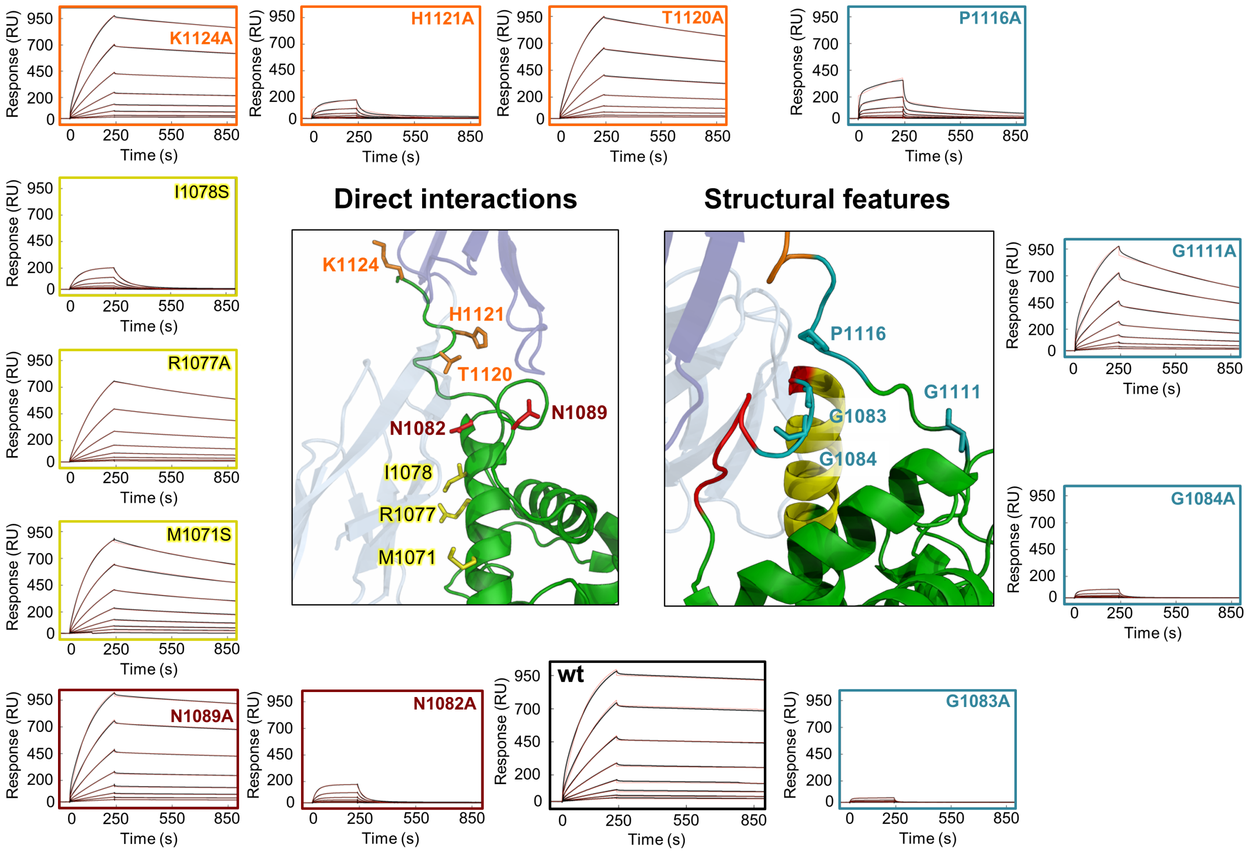

Figure S3. Detailed analysis of the ICAM-1-binding site of the PF11_0521 DBLβ3_D4 domain, *related to Figure 2*

Surface plasmon resonance analysis of the effect of binding site mutations on the interaction between PF11_0521 DBLβ3_D4 and ICAM-1^{D1D5}. The sites within the PF11_0521 DBLβ3_D4-binding site and residues within these sites that directly contact ICAM-1 are indicated (site 1, yellow; site 2, red; site 3, orange). Structural residues (teal) important for positioning of interacting residues are highlighted. For SPR measurements, ICAM-1^{D1D5}-Fc was immobilized onto the chip and wild type or mutant PF11_0521 DBLβ3_D4 were injected over the chip in two-fold dilution series from 500 nM to 4 nM. Data (black lines) are modelled to a one-site model (red lines).

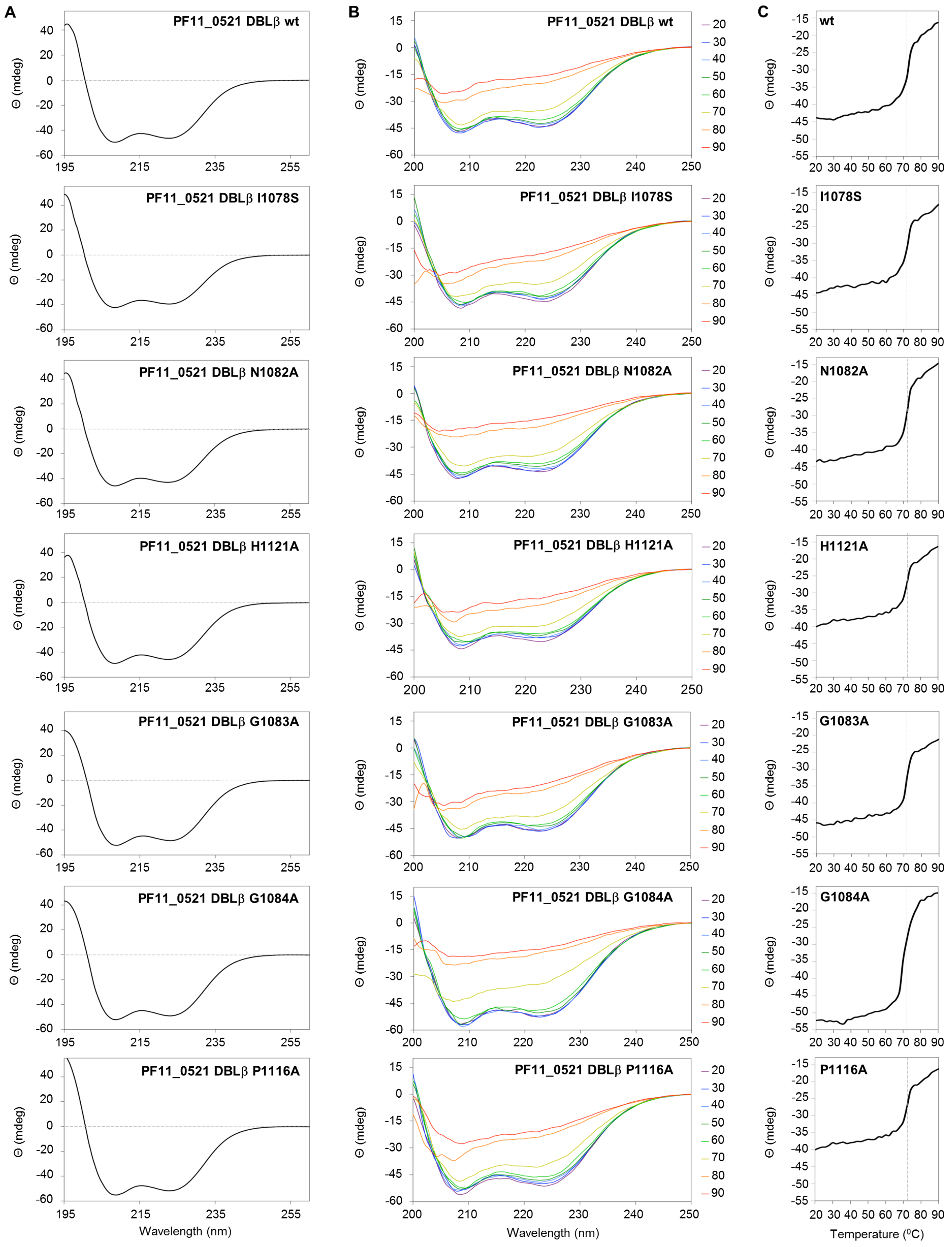

Figure S4. CD spectroscopy analysis of key PF11_0521 DBLβ3_D4 mutants, ***related to Figure 2***

(A) Secondary structure analysis of PF11_0521 DBLβ3_D4 wt, I1078S, N1082A, H1121A, G1083A, G1084A and P1116A by CD spectroscopy to confirm folding of the mutant proteins. CD spectra were recorded at 20°C between wavelengths of 195 nm and 260 nm. For each sample, four measurements were averaged and corrected for buffer absorption.

(B) Thermal denaturation analysis of PF11_0521 DBLβ3_D4 wt and mutants. CD spectra were recorded between 200 nm and 250 nm and after each measurement the temperature was raised by 0.5°C increments. Shown are spectra for 10°C intervals between 20°C and 90°C.

(C) Thermal melting curve for PF11_0521 DBLβ3_D4 wt and mutants. The ellipticity at a fixed wavelength of 222 nm was measured between 20°C and 90°C. After each measurement the temperature was increased by 0.5°C. Dashed lines indicate the respective melting temperature.

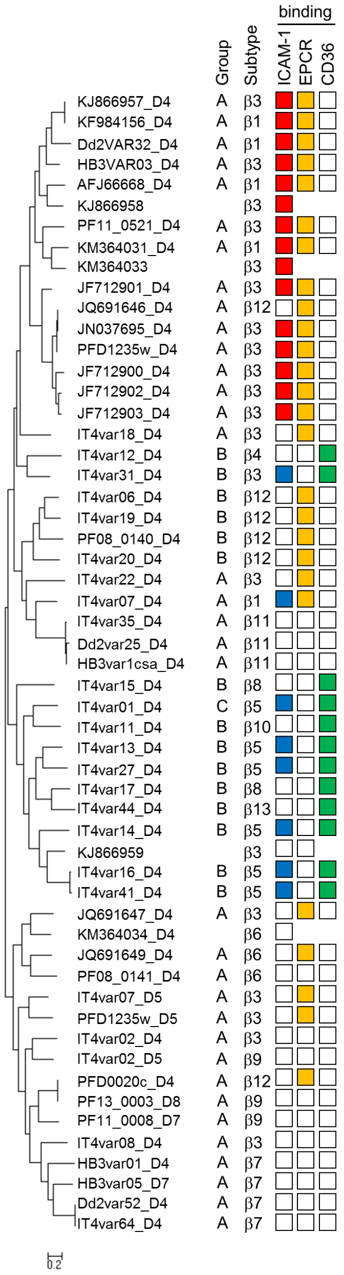

Figure S5. Phylogenetic analysis and binding phenotype of 55 DBLβ domains, *related to Figures 2 and 3*

Maximum likelihood tree of 55 complete DBLβ domains generated as described in Figure 3. Red: ICAM-1 binding DBLβ with motif; Blue: ICAM-1 binding DBLβ with no motif; Orange: EPCR binding CIDRα1; Green: CD36 binding CIDRα; White: no binding; Blank space: information not available.

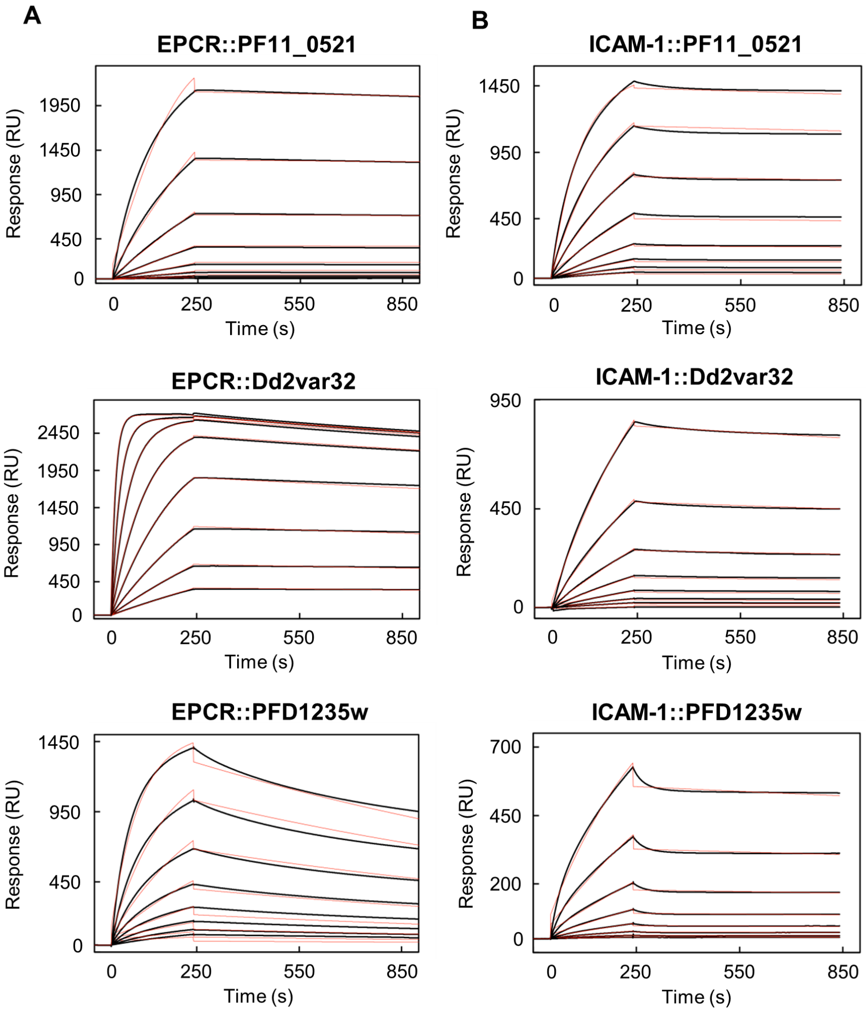

Figure S6. SPR analysis for binding of PfEMP1 head structures to ICAM-1 or EPCR, *related to Figure 5*

(A) PfEMP1 head structure binding to EPCR. EPCR was immobilized onto the chip, and PF11_0521, Dd2var32 or PFD1235w head structures were then injected over the chip surface in two-fold dilutions from 4 to 500 nM.

(B) PfEMP1 head structure binding to ICAM-1^{D1D5}. ICAM-1^{D1D5}-Fc was immobilized onto the chip and PF11_0521, Dd2var32 or PFD1235w head structures were injected over the chip surface in two-fold dilutions from 4 to 500 nM. Data (black lines) are modelled to a one-site model (red lines).

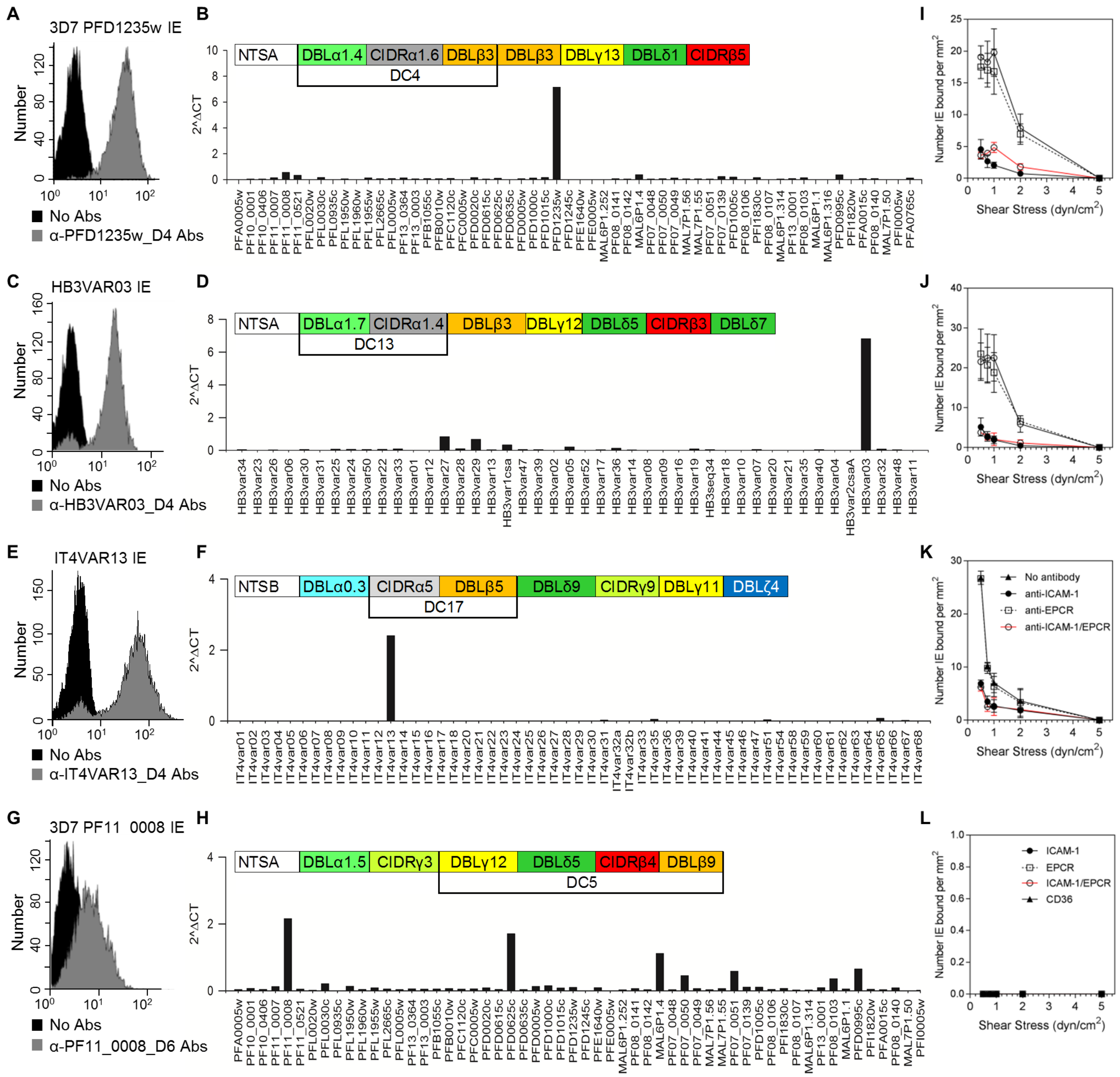

Figure S7. *var*/PfEMP1 expression profiles of HB3, IT4, and 3D7 parasite lines and adhesion to activated HBMEC and recombinant receptors, *related to Figure 6*

(A) Ethidium bromide-stained 3D7 PFD1235w IEs with (grey histogram) and without (black histogram) rat PFD1235w DBLβ3_D4 antiserum.

(B) Transcript level of each *var* gene of 3D7 PFD1235W IE relative to the control gene (*seryl-tRNA synthetase*). The domain architecture of the dominant expressed PfEMP1, PFD1235w is shown.

(C) Ethidium bromide stained HB3VAR03 IE with (grey histogram) and without (black histogram) rat VAR03_D4 antiserum.

(D) Transcript level of each *var* gene of HB3VAR03 IE relative to the control gene (*seryl-tRNA synthetase*). The domain architecture of the dominant expressed PfEMP1, VAR03 is shown.

(E) Ethidium bromide stained IT4VAR13 IE with (grey histogram) and without (black histogram) rat VAR13_D4 antiserum.

(F) Transcript level of each *var* gene of IT4VAR13 IE relative to the control gene (*seryl-tRNA synthetase*). The domain architecture of the dominant expressed PfEMP1, VAR13 is shown.

(G) Ethidium bromide stained 3D7PF11_0008 IE with (grey histogram) and without (black histogram) rat PF11_0008_D6 antiserum.

(H) Transcript level of each *var* gene of 3D7 PF11_0008 IE relative to the control gene (*seryl-tRNA synthetase*). The domain architecture of the dominant expressed PfEMP1, PF11_0008 is shown.

(I- K) Number of IE bound per mm^2 (± SEM) on HBMEC activated overnight with 10 ng/ml TNFα.

(L) Adhesion of 3D7PF11_0008 IE to recombinant CD36, EPCR and ICAM-1.

Supplemental Tables

Table S1. Data collection and refinement statistics, *related to Figure 1*

Values in parentheses are for highest-resolution shell.	PF11_0521 DBLβ3_D4-ICAM-1^{D1D2}
Data collection	
Space group	$P2_12_12_1$
Cell dimensions	
a, b, c (Å)	68.64, 109.83, 112.83
α, β, γ (°)	90.00, 90.00, 90.00
Resolution (Å)	78.70-2.78 (2.93-2.78)
R_{merge}	0.13 (0.42)
R_{pim}	0.07 (0.21)
I / σI	8.2 (3.3)
Completeness (%)	99.8 (100)
Redundancy	4.5 (4.7)
$CC_{1/2}$	0.99 (0.80)
No. unique reflections	22,210 (3,168)
Refinement	
Resolution (Å)	2.78
No. Reflections	22,036
R_{work} / R_{free}	20.48/23.56
No. Atoms	
Protein	4,970
Ligand/ion	111
Water	10
B-factors	
Protein	46.83
Ligand/ion	86.12
Water	29.67
R.m.s. deviations	
Bond lengths (Å)	0.007
Bond angles (°)	0.97

Table S2. List of contacts between PF11_0521 DBLβ3_D4 and ICAM-1^{D1D2}, *related to Figures 1 and 2*

Colors correspond to PF11_0521 DBLβ3_D4 residues as color-coded in Figures 1 and 2 of the main text.

PF11_0521 DBLβ3_D4		ICAM-1^{D1D2}		
**Residue**	**Group**	**Residue**	**Group**	**Type of Interaction**
Tyr1067	Sidechain	Pro45	Sidechain	Hydrophobic
Tyr1067	Sidechain	Leu44	Sidechain	Hydrophobic
**Met1071**	Sidechain	Val51	Sidechain	Hydrophobic
Ala1074	Sidechain	Leu42	Sidechain	Hydrophobic
**Arg1077**	Sidechain NH_2	Glu53	Sidechain O	Hydrogen bond
**Ile1078**	Sidechain	Leu18	Sidechain	Hydrophobic
**Ile1078**	Sidechain	Glu53	Sidechain	Hydrophobic
**Asn1082**	Sidechain O	Ser16	Backbone N	Hydrogen bond
**Asn1082**	Sidechain NH_2	Ser16	Backbone O	Hydrogen bond
Tyr1087	Backbone O	Tyr83	Sidechain OH	Hydrogen bond
**Asn1089**	Sidechain NH_2	Gly169	Backbone O	Hydrogen bond
Pro1117	Sidechain	Gly14	Backbone	Hydrophobic
Pro1117	Sidechain	Gly15	Backbone	Hydrophobic
Asp1119	Backbone O	Arg13	Sidechain N	Hydrogen bond
**Thr1120**	Sidechain OH	Arg13	Backbone O	Hydrogen bond
**His1121**	Sidechain N	Thr85	Sidechain OH	Hydrogen bond
**Lys1024**	Sidechain NH_3	Glu111	Sidechain O	Hydrogen bond

Table S3. Kinetic parameters derived from SPR experiments on binding between ICAM-1^{D1D5} and PF11_0521 DBLβ3_D4 wild type or mutants, *related to Figure 2*

Colors correspond to PF11_0521 DBLβ3_D4 residues as color-coded in Figure 1 of the main text.

Interaction	k_a $(x10^4\ M^{-1}\ s^{-1})$	k_d $(x10^{-5}\ s^{-1})$	K_D (nM)	R_{max} (RU)
ICAM-1^{D1D2}::PF11_0521 DBLβ **wt**	1.93	6.2	3.2	1062
ICAM-1^{D1D2}::PF11_0521 DBLβ **M1071S**	1.84	45.1	24.6	995
ICAM-1^{D1D2}::PF11_0521 DBLβ **R1077A**	1.10	38.2	34.7	1062
ICAM-1^{D1D2}::PF11_0521 DBLβ **I1078S**	1.88	2349.0	1250.0	654
ICAM-1^{D1D2}::PF11_0521 DBLβ **N1082A**	1.84	2437.0	1320.0	551
ICAM-1^{D1D2}::PF11_0521 DBLβ **N1089A**	1.79	11.4	6.3	1127
ICAM-1^{D1D2}::PF11_0521 DBLβ **T1120A**	1.41	29.8	21.1	1168
ICAM-1^{D1D2}::PF11_0521 DBLβ **H1121A**	0.61	592.2	962.0	330
ICAM-1^{D1D2}::PF11_0521 DBLβ **K1124A**	1.59	15.4	9.75	1137
ICAM-1^{D1D2}::PF11_0521 DBLβ **G1083A**	1.99	7006.0	3520.0	276
ICAM-1^{D1D2}::PF11_0521 DBLβ **G1084A**	0.97	3938.0	4060.0	583
ICAM-1^{D1D2}::PF11_0521 DBLβ **G1111A**	1.87	63.9	34.1	1048
ICAM-1^{D1D2}::PF11_0521 DBLβ **P1116A**	0.13	236.6	1690.0	1409

Table S4. The ICAM-1 binding motif is commonly found in *P. falciparum* genomes, *related to Figures 2 and 3*

Due to its size, this Table is added as a separate file.

Table S5. Clinical data related to profiling of *P. falciparum var* gene expression in children admitted to hospital with malaria, *related to Figures 4 and 7*

CM: cerebral malaria: Blantyre coma score (BCS) <3 and no other obvious clinical course for the coma; SA: severe anemia: hemoglobin <5g/dL and BCS >2; OM: other malaria, children admitted to hospital with BCS >2 and hemoglobin >5g/L

Clinical characteristics (Figure 4C)		Gr. 1 CM	Gr. 2 SA	Gr. 3 OM
Country of origin	Benin	n= 34	n= 9	n= 16
	Ghana	n= 6	n= 2	n= 9
	Tanzania	n= 13	n= 15	n= 11
Age years		3.2 (2.5; 4.8)[a]	3.7 (2.1; 4.1)	2.5 (1.6; 3.6)
		5.0 (4.7; 5.3)	2.5 (2.0; 3.0)	4.8 (4.5; 5.6)
		2.3 (1.4; 4.0)	1.0 (0.4; 2.0)	2.8 (2.2; 3.8)
Blantyre coma score		2 (2; 2)	5 (5; 5)	5 (5; 5)
		1 (0; 2)	5 (5; 5)	5 (3; 5)
		1 (0; 2)	5 (5; 5)	5 (5; 5)
Haemoglobin (g/dl)		6.7 (4.8; 7.2)	4.3 (2.9; 4.3)	7.0 (5.8; 8.9)
		7.4 (7.0; 7.6)	4.8 (4.7; 4.9)	10.5 (6.6; 11.7)
		6.6 (6.1; 8.6)	4.2 (3.3; 4.6)	11.0 (10.7; 11.8)
Parasites per µl (x1000)		36.9 (9.3; 128.0)	40.7 (9.3; 116.0)	179.4 (23.0; 496.0)
		71.4 (10.7; 96.2)	56.4 (21.5; 91.3)	77.5 (13.7; 182.9)
		31.6 (3.3; 57.4)	50.4 (7.6; 86.5)	68.8 (20.1; 78.5)

Clinical characteristics (Figure 7A and 7B)		Gr. 1 CM	Gr. 2 SA	Gr. 3 OM
Country of origin	Tanzania	n= 14	n= 21	n= 10
Age years		1.8 (1.3; 4.0)	2.2 (1.3; 2.4)	2.7 (1.5;3.2)
Blantyre coma score		2 (1; 2)	5 (5; 5)	5 (5; 5)
Haemoglobin (g/dl)		6.5 (4.6; 8.0)	3.9 (3.7; 4.4)	6.7 (5.1;8.0)
Parasites per µl (x1000)		79.1 (56.6; 144.6)	30.3 (12.6; 113.4)	35.2 (16.1; 75.7)

[a] Median (25%;75% percentiles).

Table S6. Kinetic parameters derived from SPR experiments on binding between PfEMP1 head structures and EPCR and/or ICAM-1^{D1D5}, *related to Figure 5*

Ligand	*Analyte*	*k_a ($x10^4$ M^{-1} s^{-1})*	*k_d ($x10^{-5}$ s^{-1})*	*K_D (nM)*	*R_{max} (RU)*
EPCR	**PF11_0521 HS**	3.81	4.50	1.18	2344
ICAM^{D1D5}	**PF11_0521 HS**	2.32	5.41	2.33	1533
ICAM^{D1D5}:: **PF11_0521 HS**	EPCR	1.72	19.50	11.40	228
EPCR	**DD2var32 HS**	16.20	1.29	0.79	2685
ICAM^{D1D5}	**DD2var32 HS**	0.55	1.14	20.60	1733
ICAM^{D1D5}:: **Dd2var32 HS**	EPCR	8.88	4.52	5.09	74
EPCR	**PFD1235w HS**	3.40	58.1	17.1	1164
ICAM^{D1D5}	**PFD1235w HS**	0.60	10.8	18.0	1093
ICAM^{D1D5}:: **PFD1235w HS**	EPCR	0.36	102.9	270.9	299

HS = head structure

Table S7. Statistical analysis of the effect of receptor and shear stress on adhesion of HB3VAR03, 3D7*PFD1235w*, and IT4VAR13 infected erythrocytes under physiological flow conditions and primers used for amplification of PfEMP1-specific DNA sequences and recombinant proteins produced, *related to Figures 1,2,3,4,5 and 6.*

Due to its size, this Table is added as a separate file.

Supplemental Experimental Procedures

Production of recombinant proteins and peptides

His-tagged DBLβ domains and a head structure of PFD1235w (CIDRα1.6- DBLβ3) were expressed in *E. coli* Shuffle C3030 cells (New England Biolabs) from DNA constructs generated by PCR from genomic DNA (using the primers in Table S7C) (Bengtsson et al., 2013b). A hybrid of PFD1235w DBLβ3_D4 (aa 739-1,221) and PFD1235w DBLβ3_D5 (aa 1,239-1,689) termed D5_motif was cloned by overlapping PCR amplification and expressed in *E. coli* Shuffle C3030 cells. All proteins were purified by immobilised metal ion affinity chromatography. The wild type PF11_0521 DBLβ3_D4 domain used for crystallization was further purified by ion exchange chromatography on a HiTrap S FF column (GE Healthcare) and by size exclusion chromatography using a HiLoad Superdex 75 16/60 column (GE Healthcare).

Mutants of PF11_0521 DBLβ3_D4 were generated by site-directed mutagenesis using the Quikchange protocol (Agilent Technologies). Structural mutations in the ICAM-1 binding site of PF11_0521 DBLβ3_D4 were generated using sets of 5'phosphorylated primers carrying the respective mutations. These were used to amplify a vector encoding DBLβ3_D4, followed by circularization through blunt-end ligation with T4 Ligase (Life Technologies). Mutations were verified by sequencing and the mutants were expressed and purified as described above. Triple domain head structures of PF11_0521 (NTS-DBLα1.7-CIDRα1.4-DBLβ3) and of Dd2var32 (NTS-DBLα1.7-CIDRα1.4-DBLβ1) were produced in insect cells as described (Lavstsen et al., 2012).

Recombinant ICAM-1-Fc (ICAM-1^{D1D5} and ICAM-1^{D1D2}) were expressed in HEK cells and COS-7 cells, respectively (Bengtsson et al., 2013a; Brown et al., 2013). ICAM-1^{D1D2} used for crystallization was transiently expressed as a C-terminal hexa-histidine-tagged protein in 293F cells (Life Technologies). Kifunensine (Cayman Chemical) was added directly after transfection to a final concentration of 1.5 µM. One day later, the medium was further supplemented with valproic acid (Sigma-Aldrich) at a final concentration of 2.2 mM. Seven days after transfection, the cell supernatant was harvested, sterile-filtered, concentrated, and ICAM-1^{D1D2} was purified by immobilised metal ion affinity chromatography using a NiNTA column (Qiagen), followed by size exclusion chromatography on a HiLoad Superdex 75 16/60 column (GE Healthcare). ICAM-1^{D1D5}-Fc used for SAXS experiments was cleaved with endoproteinase GluC (New England Biolabs) and the Fc tag was removed using a HiTrap Protein A HP column (GE Healthcare). Recombinant His-tagged EPCR for use in SPR experiments was expressed in S2 cells, purified and biotinylated as described (Lau et al., 2015). Recombinant EPCR used for infected erythrocyte (IE) adhesion assays was produced as described in (Nunes-Silva et al., 2015). The motif peptide (derived from PFD1235w DBLβ_D4) LYAKARIVASNGGPGYYNTEVQKKDRSVYDFLYELHLQNGGKKGPPPATHPYKSVN TRDKRDATDDTTP was produced by Schaefer-N, Denmark.

Crystallization

Purified PF11_0521 DBLβ3_D4 domain was mixed with a 1.5 molar excess of purified ICAM-1^{D1D2} and the complex was further purified and buffer exchanged by size exclusion chromatography on a HiLoad Superdex 75 16/60 column (GE Healthcare) into 10 mM HEPES, 150mM NaCl, pH 7.2. Fractions containing pure complex were pooled and concentrated to 19.1

mg/ml. Crystals were grown through vapour diffusion in sitting drops by mixing 100 nL of protein solution with 100 nL of well solution. Initial crystals grew as clusters of needles at 277 K in conditions from the Morpheus screen (Molecular Dimensions) with 10% (w/v) PEG 20000, 20% (v/v) PEG 500 and 0.1M Tris-BICINE (pH 8.5) as well solution. Crystallization conditions were further optimized by mixing 100 nL of protein solution with 50 nL of well solution and 50 nL of additive solution (Silver Bullets, Hampton Research). For cryoprotection and storage, crystals were transferred into a drop of well solution containing 16% (w/v) PEG 20000, 32% (v/v) PEG 500, 0.1 M Tris-BICINE (pH 8.5) and flash-frozen in liquid Nitrogen.

Data collection, phasing and refinement

Data were collected at beamline IO2 (Diamond Light Source, UK) using x-rays of 0.9795 Å wavelength with a Pilatus 6M detector (Dectris, Baden-Daettwil, Switzerland). The data were processed with the CCP4 program suite (Winn et al., 2011) using iMOSFLM (Battye et al., 2011) for indexing and Scala (Evans, 2006) for scaling. For molecular replacement, a search model was prepared with chainsaw using the DBLα domain of the varO PfEMP1 (PDB code 2XU0) (Juillerat et al., 2011) as template, and this model was further trimmed by removing all loops, leaving only the conserved α-helical core of the DBL domain (Higgins and Carrington, 2014). Molecular replacement with Phaser-MR (McCoy et al., 2007) using the trimmed search model as well as the individual D1 and D2 domains from ICAM-1^{D1D2} (PDB code 1IC1) (Casasnovas et al., 1998) identified one copy of the PF11_0521 DBLβ3_D4:ICAM-1^{D1D2} complex in the asymmetric unit. To minimise the effects of model bias, the initial molecular replacement solution was subjected to a round of simulated annealing using the PHENIX program suite (Adams et al., 2010). The remaining parts of the model were built through iterative

cycles of refinement using Refmac5 (Murshudov et al., 2011), Buster (Bricogne G. and Roversi P, 2016) and model building in Coot (Emsley et al., 2010). All structure figures were prepared using Pymol (Schroedinger).

Small angle x-ray scattering (SAXS)

SAXS experiments were conducted at the BM29 beamline at the European Synchrotron Radiation Facility (Grenoble, France). Data were collected at a wavelength of 0.9919 Å using a Pilatus 1M detector (Dectris, Baden-Daettwil, Switzerland) with a sample-detector distance of 2.867 m. All samples for SAXS were purified and buffer-exchanged by size-exclusion chromatography into 20 mM HEPES, 150 mM NaCl, pH 7.4. The sample purity was checked by SDS-PAGE and only samples with a purity of at least 95% were used for SAXS experiments. Prior to measurements, all samples were filtered through a 0.1 μm centrifugal spin-filter (Millipore). Data for each sample were collected at 20°C, and before and after each sample buffer was measured for background subtraction.

For data analysis, scattering curves were manually inspected in PRIMUS (Konarev et al., 2003). Frames showing signs of radiation damage were omitted from further analysis. Remaining scattering curves were averaged, and averaged buffer curves were subtracted. The radius of gyration (R_g) was determined by Guinier analysis using AutoRg in PRIMUS. GNOM (Svergun, 1992) was used to determine the pair-distance distribution function P(r) and the maximum particle diameter (D_{max}).

To generate volumetric representations based on the SAXS data, 20 independent *ab initio* bead models were calculated using DAMMIF (Franke and Svergun, 2009) with default parameters. These models were then averaged using DAMAVER (Volkov and Svergun, 2003), and the averaged model was further refined against the original data using DAMMIN. Envelopes were calculated from the refined bead models using Situs. The crystal structures of PF11_0521 DBLβ3_D4, the PF11_0521 DBLβ3_D4:ICAM-1^{D1D2} complex as well as ICAM-1^{D3D5} (PDB code 2OZ4) (Chen et al., 2007) were docked into these envelopes using Sculptor (Birmanns et al., 2011). CRYSOL (Svergun et al., 1995) was used for fitting the experimental solution scattering data of the PF11_0521 DBLβ3_D4:ICAM-1^{D1D2} complex to the theoretical scattering based on the crystal structure.

Surface plasmon resonance (SPR)

SPR experiments were carried out on a Biacore T-200 instrument (GE Healthcare). For analysis of wildtype and mutant PF11_0521 DBLβ3_D4 binding to ICAM-1, ICAM-1^{D1D5} Fc was immobilized to 2100 response units (RU) on a CM5 chip (GE Healthcare) pre-coupled to Protein A. Wild type and mutant PF11_0521 DBLβ3 constructs were buffer-exchanged into 10 mM HEPES pH 8.0, 300 mM NaCl, 0.05% Tween-20. Concentration series (two-fold dilution from 4 nM to 500 nM) were flowed at 40 μL/min over the chip with 240 s association and 660 s dissociation times. After each run, the chip was regenerated by injecting 10 mM Glycine, pH 2.0 for 120 s at 10 μL/min.

For analysis of binding of PfEMP1 head structures to EPCR, biotinylated EPCR was immobilized to 650 RU on a CAP chip using the Biotin Capture Kit (GE Healthcare). All head

structures were buffer-exchanged into 10 mM HEPES (pH 7.4), 300 mM NaCl, 0.05% Tween-20, 2 mg/ml ssDNA (Panreac AppliChem). Concentration series (two-fold dilution from 500 nM to 4 nM) were flowed over the chip at 30 μL/min with 240 s association and 720 s dissociation times. The chip was regenerated between runs by injecting CAPture Kit regeneration solution (GE Healthcare) for 120 s at 10 μL/min.

To measure binding of PfEMP1 head structures to ICAM-1, ICAM-1^{D1D5} Fc was immobilized to 1,400 RU on a CM5 chip (GE Healthcare) pre-coupled to Protein A. All head structures were buffer-exchanged into 10 mM HEPES (pH 8.0), 300 mM NaCl, 0.05% Tween-20. Concentration series (two-fold dilution from 4 nM to 500 nM) were flowed at 40 μL/min over the chip with 240 s association and 600 s dissociation times. The chip was regenerated between runs by injecting 10 mM Glycine (pH 2.0) for 120 s at 10 μL/min.

For analysis of dual adhesion of PfEMP1 head structures to ICAM-1 and EPCR, ICAM-1^{D1D5} Fc was immobilized to 1,800 RU (for experiments with PF11_0521 head structure), 2,100 RU (for experiments with Dd2var32 head structure) or 1,500 RU (for experiments with PFD1235w head structure) on a CM5 chip (GE Healthcare) pre-coupled to Protein A. All head structures were buffer-exchanged into 10 mM HEPES (pH 8.0), 300 mM NaCl, 0.05% Tween-20, and were then injected over the ICAM-1 immobilized on the chip at a fixed concentration of 250 nM for 240 s at 20 μL/min. Then, a concentration series (two-fold dilution from 250 nM to 16 nM) of EPCR (buffer-exchanged into 10 mM HEPES (pH 8.0), 300 mM NaCl, 0.05% Tween-20) was flowed over the chip at 30 μL/min with 240 s association and 720 s dissociation times. After each run, the chip was regenerated by injecting 10 mM Glycine (pH 2.0) for 120 s at 10 μL/min.

SPR data were analysed using the BIAevaluation software 2.0.3 (GE Healthcare). Values for k_a, k_d and K_D were determined by globally fitting curves corresponding to at least 5 concentrations into a one-site kinetic model. All SPR experiments were performed in duplicates, and curves shown are representatives of these measurements.

Circular Dichroism Spectroscopy

All circular dichroism (CD) spectroscopy experiments were conducted on a J-815 Spectropolarimeter, connected to a Peltier temperature control unit. The samples were dialyzed against 100mM sodium phosphate buffer, 200mM NaF, pH 7.2 and diluted to 0.3 mg/ml. CD spectra and spectra for thermal denaturation assays were recorded as described previously (Lennartz et al., 2015).

Patient samples

P. falciparum isolates were collected from malaria patients (Table S5) for transcription analysis. This was performed at Hohoe Municipal Hospital in the Volta Region of Ghana (Stevenson et al., 2015), at Korogwe District Hospital in Korogwe District in Northeastern Tanzania (Lavstsen et al., 2012), and at Centre National Hospitalier Universitaire Hubert Koutoucou Mega in Cotonou, Benin (Moussiliou et al., 2015). The studies were approved by the Ethical Review Committee of the Ghana Health Services (file GHS-ERC 08/05/14), the Ethical Review Board of the National Institute for Medical Research, Tanzania (National Institute of Medical Research/HQ/R.8a/Vol.IX/559), and by the Ethics Committee of the Research Institute of Applied Biomedical Sciences, Cotonou, Benin (N°21/CER/ISBA/13), respectively.

After informed consent had been obtained from a parent or a guardian, enrolled children were subjected to clinical investigation, collection of venous blood samples, and treatment according to national guidelines. Pelleted erythrocytes were dissolved in 9 volumes of Trizol reagent (Thermo Fisher Scientific) for RNA preservation at minus 80°C.

Clinical manifestations of malaria were classified according to the definitions and associated criteria of the World Health Organization. Patients were categorized as having cerebral malaria (CM) if they had a positive blood smear of the asexual form of *P. falciparum*, unrousable coma (Blantyre coma score, BCS ≤2) with exclusion of other causes of coma and severe illness. Patients were classified as having severe malarial anemia (SA) if hemoglobin < 5g/dL and BCS >2. Patients were classified as having other malaria (OM) if BCS >2 and hemoglobin >5 g/dL.

Additional samples used were, plasma samples from 76 *P. falciparum*-exposed Tanzanian children (1-17 years) collected during a cross-sectional study monitoring risk factors for anaemia and febrile malaria and nine plasma samples from *P. falciparum*- exposed adult Liberians. These samples were from previous studies described in (Lusingu et al., 2004; Theisen et al., 2000).

Antibodies

Rat immunizations and IgG preparations were done as described (Bengtsson et al., 2013b). All experimental animal procedures were approved by The Danish Animal Procedures Committee ("Dyreforsøgstilsynet") as described in permit no. 2008/561-1498 and according to the guidelines described in Danish act LBK 1306 (23/11/2007) and BEK 1273 (12/12/2005). DBLβ

and motif-specific rat IgG (anti-PFD1235w_D4; anti-PFD1235w_motif; anti-IT4VAR13) were affinity purified from rat antisera as described (Joergensen et al., 2010). Pre-immunization serum or rat IgG (Sigma Aldrich) were used as negative controls in binding assays. Pooled plasma from nine *P. falciparum*-exposed adult Liberians (Theisen et al., 2000) were used for affinity purification of DBLβ- and motif-specific human IgG (anti-PFD1235w_D4; anti-PFD1235w_motif; anti-IT4VAR13). Human IgG (Sigma Aldrich) was used as negative control in binding assays.

ELISA

ICAM-1 binding was assessed by ELISA using Maxisorp plates coated with 26 different recombinant DBLβ proteins (2-10 μg/ml) (Bengtsson et al., 2013b). Of these, eight DBLβ proteins had known ability to bind ICAM-1 (Bengtsson et al., 2013b), but were identified here as having the motif prior to doing the ELISA. Additionally eight DBLβ proteins were predicted as ICAM-1 binders and ten as non-ICAM-1 binders based on sequence analysis done in this study prior to doing ELISA. An additional eight DBLβ proteins without the motif were previously tested for ICAM-1 binding in the laboratory (Figure 3A). Binding was detected using rabbit anti-human IgG-HRP (Dako; 1:3,000) targeting the Fc-portion of the ICAM-1-Fc.

ELISA was similarly used to analyze motif-specific IgG reactivity and the ICAM-1 anti-adhesive effect of individual human immune plasma (1:100) from *P. falciparum*-exposed individuals (1 - 17 years) living in an area of high parasite transmission in northeastern Tanzania (Lusingu et al., 2004). Bound human IgG was detected using rabbit anti-human IgG as described above. For adhesion inhibition assays using affinity purified rat or human IgG (0.25-32 μg/ml) dilutions

were added to ICAM-1-Fc-coated plates simultaneously with DBLβ protein. A HRP-conjugated penta-His antibody (Qiagen; 1:3,000) was used for detection of bound DBLβ.

Plasmodium falciparum parasite culture

The *P. falciparum* clones 3D7, HB3, and IT4 were maintained *in vitro* and selected with antibodies to express specific PfEMP1 proteins as described (Bengtsson et al., 2013b; Joergensen et al., 2010). *P. falciparum* 3D7 infected erythrocytes (IEs) were selected with the human monoclonal antibody AB01, which targets PFD1235w DBLγ13_D6 (Barfod et al., 2011) or with a polyclonal rat anti-serum against PF11_0008 CIDRβ4_D6. *P. falciparum* IT4 IEs were similarly selected using a rat anti-serum against IT4VAR13 DBLβ5_D4, whereas *P. falciparum* HB3 IEs were antibody-selected using a rat anti-serum against HB3VAR03 DBLβ3_D4. In all cases, PfEMP1 expression was regularly monitored by flow cytometry using PfEMP1-specific antisera, and only cultures with more than 60% antibody-labeled IEs were used in the study. The identity and clonality of all parasites used were routinely verified by genotyping as described (Snounou et al., 1999). Mycoplasma infection was excluded regularly using the MycoAlert Mycoplasma Detection Kit (Lonza) according to the manufacturer's instructions.

Flow Cytometry

P. falciparum IEs were DNA-labeled with ethidium bromide and surface-labeled with rat antibodies as described (Joergensen et al., 2010). Rat antisera (anti-PFD1235w_D4; anti-HB3VAR03_D4; anti-PF11_0008_D6; anti-IT4VAR13) were used at a 1:40 dilution. IE-bound antibodies were labeled using FITC-conjugated secondary rabbit anti-rat IgG (1:150, Vector Labs). FITC fluorescence data from ethidium bromide-positive cells were collected on a

Cytometic FC500 MPL flow cytometer (Beckman Coulter) and data analysed using WinList version 6.0 (Verity Software House).

IE adhesion to recombinant receptors

Microslides ($VI^{0.1}$; Ibidi) were coated overnight with recombinant ICAM-1-Fc (50 µg/ml) (Bengtsson et al., 2013a), EPCR-his (10 µg/ml) (Nunes-Silva et al., 2015) or with recombinant CD36 (20 µg/ml, R&D Systems). For endothelial adhesion assays, human brain microvascular endothelial cells (HBMEC at passage 2-5; Sciencell) were seeded onto fibronectin (2 $\mu g/cm^{2;}$ Millipore) pre-coated venaEC ($5x10^5$ cells/ml) or microslides ($VI^{0.4}$; ibidi. $3x10^5$ cells/ml) for 72 – 96 hours. Activation of HBMEC was done by adding TNFα (10 ng/ml; Sinobiological) for 18 hours. Mature IEs were adjusted to 3% parasitaemia in RPMI-1640 supplemented with 2% normal human serum. The IEs were then subjected to physiological shear stress ranging from 0.5-5 $dyne/cm^2$ as described (Lennartz et al., 2015). After 5 minutes of flow, the number of bound IE/mm^2 in five separate fields was counted at 20× magnification using a Leica inverted phase contrast microscope. Bound IE were dislodged after each run, and the shear stress increased to measure the ability of IE to initiate adhesion at each shear stress. A minimum of three independent experiments in triplicate were performed. To assess the capacity of antibodies to inhibit IE adhesion, the IEs were pre-incubated with affinity-purified IgG or plasma as described above. Affinity-purified non-immune rat or human IgG were included as negative controls. The specificity of IE adhesion to ICAM-1-Fc, EPCR-his, rCD36 and HBMEC was verified by pre-incubating flow channels with 40 µg/ml anti-ICAM-1 antibody (clone 15.2, AbD Serotec), 10 µg/ml anti-EPCR (polyclonal, R&D Systems) or with 20 µg/ml anti-CD36 (monoclonal, Beckman Coulter). All assays were blinded.

Immunofluorescence assay

Dual binding of IE to ICAM-1 and EPCR was assessed by incubating the IEs expressing PFD1235w, HB3VAR03, or IT4VAR13 with preformed antibody:protein complexes. Briefly, equal molar amounts of ICAM-1-Fc and goat-F(ab)$_2$-anti-human IgG-Fc Dylight 488 (Abcam) or EPCR-his with mouse-anti-PENTA-His (Qiagen) and anti-mouse IgG-Alexa 568 (Abcam) were incubated at 4°C for 4 hours. After incubation, complexes were aliquoted and stored at -20°C until use. IEs were washed in PBS, incubated with 2% NHS for 10 minutes at 37°C, and washed twice with 1× PBS. Aliquots of the antibody:protein complex were thawed and diluted 10x in 2% Ig-free BSA/PBS before adding to IEs and incubating at 37°C for 10 minutes with gentle shaking. IEs were then washed 3× with 2% Ig-free BSA/PBS. Thin smears were air-dried and mounted with ProLong Gold Antifade DAPI mounting medium (Life Technologies) and examined using a Zeiss AxioImager Z1 and a 63× oil inversion lens. Composite images were compiled with Zen Blue (Zeiss) and Fiji-ImageJ open source biological imaging software (Schindelin et al., 2012).

Bioinformatic analysis

A total of 151 DBLβ domains from seven previously annotated whole parasite genomes (Rask et al., 2010) were used to blastp extract DBLβ sequences from assemblies of Illumina whole-genome sequencing data, as described previously (Lau et al., 2015). This analysis included sequence data from 226 *P. falciparum* genomes from Africa and Asia (study number ERP000190), made available through the MalariaGen community (Miotto et al., 2015), and additional sequence data available through PlasmoDB, NCBI, and the Broad Institute. DBLβ

domains predicted to bind ICAM-1 were identified using the search term I[V/L]x_3N[E]GG[P/A]xYx_{27}GPPx_3H. A WebLogo 3 sequence logo (Crooks et al., 2004) (http://weblogo.three plusone.com/) was generated based on alignment of 145 protein sequences with the ICAM-1 binding motif (Table S4). The average amino acid identity of DBLβ domains was calculated using the Praline multiple sequence alignment tool (http://www.ibi.vu.nl/programs/pralinewww/) using default settings.

Frequencies and distribution of motif-containing DBLβ domains in relation to PfEMP1 group and adjacent CIDR domain types was estimated from counting domain co-occurences in the *var* sequence contigs assembled from the 233 *P. falciparum* genomes. Multiple alignments were made using MUSCLE v. 3.7 software (Edgar, 2004). Relatedness of DBLβ domains and subdomain 3 sequence was inferred by using the Maximum Likelihood method based on the Whelan And Goldman plus Freq. model (Whelan and Goldman, 2001) using MEGA6 (Tamura et al., 2007).The tree with the highest log likelihood (Figure 3A (-35460.7853) and Figure 3B (-179728.3832)) is shown. Initial tree(s) for the heuristic search were obtained automatically by applying Neighbor-Join and BioNJ algorithms to a matrix of pairwise distances estimated using a JTT model, and then selecting the topology with superior log likelihood value. The trees were drawn to scale, with branch lengths measured in the number of substitutions per site. The analysis in Figure 3A involved 55 amino acid sequences with a total of 664 positions in the final dataset. In Figure 3B, a discrete Gamma distribution was used to model evolutionary rate differences among sites (3 categories (+G, parameter = 0.8099)). The rate variation model allowed for some sites to be evolutionarily invariable ([+I], 5.9495% sites). All positions with less than 95% site coverage were eliminated. That is, fewer than 5% alignment gaps, missing

data, and ambiguous bases were allowed at any position. There were a total of 145 positions in the final dataset.

Amplicons representing *var* genes from eight different Ghanaian *P. falciparum* isolates (Nielsen et al., 2002) were amplified by PCR using primer combination M3637 (5′-TGCTNKTAATGGTGGTCC-3′)/Exon2 LNA (Lavstsen et al., 2012) or varF_dg2/Exon2 LNA (both (Lavstsen et al., 2012)), fragmented by sonication, built into sequencing libraries and sequenced with a 250 bp paired-end protocol on an Illumina® MiSeq. The domains of new PfEMP1 sequences were classified using the Hidden Markov Model approach, and named as described previously (Rask et al., 2010).

Additional *var* gene sequence data from 45 Tanzanian children with severe or uncomplicated *P. falciparum* malaria (Jespersen et al., 2016) were analyzed using the ICAM-1 binding motif presented here. Briefly, profiles of expressed *var* genes were obtained using the DBLα-tag PCR method described elsewhere (Lavstsen et al., 2012). The top six most abundant *var* tags (accounting for an average of 74% [range 38% to 100%] of all expressed *var* genes) in each patient were targeted for further analysis. Specific primers were designed for the relevant DBLα-tag sequences and used for long-range PCR in combination with a universal primer annealing in Exon 2 (Lavstsen et al., 2012). The resulting PCR products were Illumina-sequenced, assembled, and annotated. RT-qPCR measurements using specific primers were subsequently done to control for any potential amplification bias. For each patient, the relative abundance of each of the top-six *var* genes was expressed as a percentage of all the top-six *var* genes in that patient. Following this, normalized relative abundances were adjusted according to the relative

abundance measured by RT-qPCR. This method may in a few patients miss highly expressed transcript variants due to primer amplification bias, and is less suited for quantification of the total level of a given transcript trait within a patient, or patient group, than qPCR. However, in contrast to qPCR, using primers targeting pre-defined loci, the method allows subsequent sequence analysis of expressed genes. From this identification and based on the relative transcript abundance of these highly transcribed *var* genes, the percentage of transcripts coding for motif-containing ICAM-1-binding DBLβ and EPCR-binding CIDRα1 was determined in each of the 45 children.

Real-time quantitative PCR (RT-qPCR)

Parasite RNA from clinical *P. falciparum* isolates was reverse-transcribed into cDNA and subjected to RT-qPCR using primer pairs (Table S7C) targeting *var* gene sub-classes encoding group A DBLβ domains predicted to bind to ICAM-1 (motif: primers Q183/Q186) and/or *var* gene sub-classes encoding EPCR-binding CIDRα1 domains (EPCR: primers "DBLa2/1.1/2/4/7", Makumbaye et al. submitted). Quanti-Tect SYBR Green PCR Master Mix (Qiagen) and a Rotorgene thermal cycle system (Corbett Research) were used as described (Jensen et al., 2004). The abundance of *var* gene transcripts was calculated relative to the transcript level of the endogenous control gene Seryl-tRNA synthetase ($\Delta Ct_{var\_gene} = Ct_{var\_gene} - Ct_{endogenous\ control\ gene}$). Delta Ct (*var* gene) values were translated into transcription units (Tu) as described (Lavstsen et al., 2012). Values of $\Delta Ct_{var\_gene} \geq 5$ were assigned a value of 5 and Tu calculated as $Tu = 2^{(5-\Delta Ct var\_gene)}$. Thus, ΔCt_{var_gene} values ≥5 were assigned a Tu-value of 1 with ΔCt_{var_gene} values of 4 corresponding to Tu = 2; $\Delta Ct_{var\_gene} = 3$:⇔ Tu =4; $\Delta Ct_{var\_gene} = 2$:⇔ Tu =8; $\Delta Ct_{var\_gene} = 1$:⇔

Tu = 16; $\Delta Ct_{var\_gene} = 0 :\Leftrightarrow$ Tu = 32 corresponding to the mean level of the transcription of the endogenous control gene.

Statistical analysis

Patients were assigned to three clinical groups: i) children with a Blantyre score of <3 (CM); ii) children with a Blantyre score >2 and hemoglobin <5 g/dL (SA); iii) *P. falciparum* positive children with a Blantyre score >2 and hemoglobin > 5 g/dL (other malaria, OM). *Var* gene transcript levels (Figure 4C) and percentages of transcripts (Figure 7) were compared using the Wilcoxon-Mann-Whitney rank sum test. ICAM-1 transcript levels (Figure 4C) showed no statistical significant difference between SA and OM children.

Transcription data from these two groups were therefore pooled and compared to the data from group i. To analyze if study site (Figure 4C) influenced the association between ICAM-1 transcript levels and clinical outcome of infection a comparison was done using a logistic regression model with cerebral malaria as outcome variable and study site (Benin, Ghana, and Tanzania) as well as ICAM-1 transcript level as explanatory variables. Inhibition of ICAM-1 binding by antibodies (Figure 4B) was compared using the Wilcoxon-Mann-Whitney rank sum test using SigmaPlot 13.0 (Systat Software Inc, United Kingdom), all other analysis presented were done using STATA14 software (StataCorp, USA).

Supplemental References

Adams, P.D., Afonine, P.V., Bunkoczi, G., Chen, V.B., Davis, I.W., Echols, N., Headd, J.J., Hung, L.W., Kapral, G.J., Grosse-Kunstleve, R.W., *et al.* (2010). PHENIX: a comprehensive Python-based system for macromolecular structure solution. Acta Crystallogr D Biol Crystallogr *66*, 213-221.

Avril, M., Bernabeu, M., Benjamin, M., Brazier, A.J., and Smith, J.D. (2016). Interaction between Endothelial Protein C Receptor and Intercellular Adhesion Molecule 1 to Mediate Binding of Plasmodium falciparum-Infected Erythrocytes to Endothelial Cells. MBio *7*.

Avril, M., Tripathi, A.K., Brazier, A.J., Andisi, C., Janes, J.H., Soma, V.L., Sullivan, D.J., Jr., Bull, P.C., Stins, M.F., and Smith, J.D. (2012). A restricted subset of var genes mediates adherence of Plasmodium falciparum-infected erythrocytes to brain endothelial cells. ProcNatlAcadSciUSA *109*, E1782-E1790.

Barfod, L., Dalgaard, M.B., Pleman, S.T., Ofori, M.F., Pleass, R.J., and Hviid, L. (2011). Evasion of immunity to Plasmodium falciparum malaria by IgM masking of protective IgG epitopes in infected erythrocyte surface-exposed PfEMP1. ProcNatlAcadSciUSA *108*, 12485-12490.

Battye, T.G., Kontogiannis, L., Johnson, O., Powell, H.R., and Leslie, A.G. (2011). iMOSFLM: a new graphical interface for diffraction-image processing with MOSFLM. Acta Crystallogr D Biol Crystallogr *67*, 271-281.

Bengtsson, A., Joergensen, L., Barbati, Z.R., Craig, A., Hviid, L., and Jensen, A.T. (2013a). Transfected HEK293 cells expressing functional recombinant intercellular adhesion molecule 1 (ICAM-1)--a receptor associated with severe Plasmodium falciparum malaria. PLoSOne *8*, e69999.

Bengtsson, A., Joergensen, L., Rask, T.S., Olsen, R.W., Andersen, M.A., Turner, L., Theander, T.G., Hviid, L., Higgins, M.K., Craig, A., *et al.* (2013b). A novel domain cassette identifies Plasmodium falciparum PfEMP1 proteins binding ICAM-1 and is a target of cross-reactive, adhesion-inhibitory antibodies. JImmunol *190*, 240-249.

Bernabeu, M., Danziger, S.A., Avril, M., Vaz, M., Babar, P.H., Brazier, A.J., Herricks, T., Maki, J.N., Pereira, L., Mascarenhas, A., *et al.* (2016). Severe adult malaria is associated with specific PfEMP1 adhesion types and high parasite biomass. Proc Natl Acad Sci U S A *113*, E3270-3279.

Birbeck, G.L., Molyneux, M.E., Kaplan, P.W., Seydel, K.B., Chimalizeni, Y.F., Kawaza, K., and Taylor, T.E. (2010). Blantyre Malaria Project Epilepsy Study (BMPES) of neurological outcomes in retinopathy-positive paediatric cerebral malaria survivors: a prospective cohort study. Lancet Neurol *9*, 1173-1181.

Birmanns, S., Rusu, M., and Wriggers, W. (2011). Using Sculptor and Situs for simultaneous assembly of atomic components into low-resolution shapes. J Struct Biol *173*, 428-435.

Bricogne G., B.E., Brandl M., Flensburg C., Keller P., Paciorek W.,, and Roversi P, S.A., Smart O.S., Vonrhein C., Womack T.O. (2016). BUSTER version 2.10.2. Global Phasing Ltd Cambridge, UK.

Brown, A., Turner, L., Christoffersen, S., Andrews, K.A., Szestak, T., Zhao, Y., Larsen, S., Craig, A.G., and Higgins, M.K. (2013). Molecular architecture of a complex between an adhesion protein from the malaria parasite and intracellular adhesion molecule 1. JBiolChem *288*, 5992-6003.

Casasnovas, J.M., Stehle, T., Liu, J.H., Wang, J.H., and Springer, T.A. (1998). A dimeric crystal structure for the N-terminal two domains of intercellular adhesion molecule-1. Proc Natl Acad Sci U S A *95*, 4134-4139.

Chen, X., Kim, T.D., Carman, C.V., Mi, L.Z., Song, G., and Springer, T.A. (2007). Structural plasticity in Ig superfamily domain 4 of ICAM-1 mediates cell surface dimerization. Proc Natl Acad Sci U S A *104*, 15358-15363.

Claessens, A., Adams, Y., Ghumra, A., Lindergard, G., Buchan, C.C., Andisi, C., Bull, P.C., Mok, S., Gupta, A.P., Wang, C.W., *et al.* (2012). A subset of group A-like var genes encodes the malaria parasite ligands for binding to human brain endothelial cells. Proc Natl Acad Sci U S A *109*, E1772-1781.

Crooks, G.E., Hon, G., Chandonia, J.M., and Brenner, S.E. (2004). WebLogo: a sequence logo generator. Genome Res *14*, 1188-1190.

Cunnington, A.J., Riley, E.M., and Walther, M. (2013). Stuck in a rut? Reconsidering the role of parasite sequestration in severe malaria syndromes. Trends Parasitol *29*, 585-592.

Dondorp, A.M., Fanello, C.I., Hendriksen, I.C., Gomes, E., Seni, A., Chhaganlal, K.D., Bojang, K., Olaosebikan, R., Anunobi, N., Maitland, K.*, et al.* (2010). Artesunate versus quinine in the treatment of severe falciparum malaria in African children (AQUAMAT): an open-label, randomised trial. Lancet *376*, 1647-1657.

Doumbo, O.K., Thera, M.A., Kone, A.K., Raza, A., Tempest, L.J., Lyke, K.E., Plowe, C.V., and Rowe, J.A. (2009). High levels of Plasmodium falciparum rosetting in all clinical forms of severe malaria in African children. Am J Trop Med Hyg *81*, 987-993.

Edgar, R.C. (2004). MUSCLE: multiple sequence alignment with high accuracy and high throughput. Nucleic Acids Res *32*, 1792-1797.

Emsley, P., Lohkamp, B., Scott, W.G., and Cowtan, K. (2010). Features and development of Coot. Acta Crystallogr D Biol Crystallogr *66*, 486-501.

Evans, P. (2006). Scaling and assessment of data quality. Acta Crystallogr D Biol Crystallogr *62*, 72-82.

Franke, D., and Svergun, D.I. (2009). DAMMIF, a program for rapid ab-initio shape determination in small-angle scattering. Journal of Applied Crystallography *42*, 342-346.

Goel, S., Palmkvist, M., Moll, K., Joannin, N., Lara, P., Akhouri, R.R., Moradi, N., Ojemalm, K., Westman, M., Angeletti, D.*, et al.* (2015). RIFINs are adhesins implicated in severe Plasmodium falciparum malaria. Nat Med *21*, 314-317.

Higgins, M.K., and Carrington, M. (2014). Sequence variation and structural conservation allows development of novel function and immune evasion in parasite surface protein families. Protein Sci *23*, 354-365.

Howell, D.P., Levin, E.A., Springer, A.L., Kraemer, S.M., Phippard, D.J., Schief, W.R., and Smith, J.D. (2008). Mapping a common interaction site used by *Plasmodium falciparum* Duffy binding-like domains to bind diverse host receptors. MolMicrobiol *67*, 78-87.

Hsieh, F.L., Turner, L., Bolla, J.R., Robinson, C.V., Lavstsen, T., and Higgins, M.K. (2016). The structural basis for CD36 binding by the malaria parasite. Nat Commun *7*, 12837.

Hviid, L., and Jensen, A.T. (2015). PfEMP1 - a parasite protein family of key importance in *Plasmodium falciparum* malaria immunity and pathogenesis. AdvParasitol *88*, 51-84.

Idro, R., Jenkins, N.E., and Newton, C.R. (2005). Pathogenesis, clinical features, and neurological outcome of cerebral malaria. Lancet Neurol *4*, 827-840.

Janes, J.H., Wang, C.P., Levin-Edens, E., Vigan-Womas, I., Guillotte, M., Melcher, M., Mercereau-Puijalon, O., and Smith, J.D. (2011). Investigating the host binding signature on the Plasmodium falciparum PfEMP1 protein family. PLoSPathog *7*, e1002032.

Jensen, A.T., Magistrado, P., Sharp, S., Joergensen, L., Lavstsen, T., Chiucchiuini, A., Salanti, A., Vestergaard, L.S., Lusingu, J.P., Hermsen, R.*, et al.* (2004). *Plasmodium falciparum* associated with severe childhood malaria preferentially expresses PfEMP1 encoded by group A *var* genes. JExpMed *199*, 1179-1190.

Jespersen, J.S., Wang, C.W., Mkumbaye, S.I., Minja, D.T., Petersen, B., Turner, L., Petersen, J.E., Lusingu, J.P., Theander, T.G., and Lavstsen, T. (2016). Plasmodium falciparum var genes expressed in children with severe malaria encode CIDRalpha1 domains. EMBO Mol Med.

Joergensen, L., Bengtsson, D.C., Bengtsson, A., Ronander, E., Berger, S.S., Turner, L., Dalgaard, M.B., Cham, G.K., Victor, M.E., Lavstsen, T.*, et al.* (2010). Surface co-expression of two different PfEMP1 antigens on single Plasmodium falciparum-infected erythrocytes facilitates binding to ICAM1 and PECAM1. PLoSPathog *6*, e1001083.

Juillerat, A., Lewit-Bentley, A., Guillotte, M., Gangnard, S., Hessel, A., Baron, B., Vigan-Womas, I., England, P., Mercereau-Puijalon, O., and Bentley, G.A. (2011). Structure of a Plasmodium falciparum PfEMP1 rosetting domain reveals a role for the N-terminal segment in heparin-mediated rosette inhibition. Proc Natl Acad Sci U S A *108*, 5243-5248.

Kim, J.H., Singvall, J., Schwarz-Linek, U., Johnson, B.J., Potts, J.R., and Hook, M. (2004). BBK32, a fibronectin binding MSCRAMM from Borrelia burgdorferi, contains a disordered region that undergoes a conformational change on ligand binding. J Biol Chem *279*, 41706-41714.

Konarev, P.V., Volkov, V.V., Sokolova, A.V., Koch, M.H.J., and Svergun, D.I. (2003). PRIMUS: a Windows PC-based system for small-angle scattering data analysis. Journal of Applied Crystallography *36*, 1277-1282.

Kwong, P.D., Wyatt, R., Robinson, J., Sweet, R.W., Sodroski, J., and Hendrickson, W.A. (1998). Structure of an HIV gp120 envelope glycoprotein in complex with the CD4 receptor and a neutralizing human antibody. Nature *393*, 648-659.

Lau, C.K., Turner, L., Jespersen, J.S., Lowe, E.D., Petersen, B., Wang, C.W., Petersen, J.E., Lusingu, J., Theander, T.G., Lavstsen, T.*, et al.* (2015). Structural conservation despite huge sequence diversity allows EPCR binding by the PfEMP1 family implicated in severe childhood malaria. Cell HostMicrobe *17*, 118-129.

Lavstsen, T., Turner, L., Saguti, F., Magistrado, P., Rask, T.S., Jespersen, J.S., Wang, C.W., Berger, S.S., Baraka, V., Marquard, A.M.*, et al.* (2012). Plasmodium falciparum erythrocyte membrane protein 1 domain cassettes 8 and 13 are associated with severe malaria in children. ProcNatlAcadSciUSA *109*, E1791-E1800.

Lennartz, F., Bengtsson, A., Olsen, R.W., Joergensen, L., Brown, A., Remy, L., Man, P., Forest, E., Barfod, L.K., Adams, Y.*, et al.* (2015). Mapping the Binding Site of a Cross-Reactive Plasmodium falciparum PfEMP1 Monoclonal Antibody Inhibitory of ICAM-1 Binding. JImmunol *195*, 3273-3283.

Lusingu, J.P., Vestergaard, L.S., Mmbando, B.P., Drakeley, C.J., Jones, C., Akida, J., Savaeli, Z.X., Kitua, A.Y., Lemnge, M.M., and Theander, T.G. (2004). Malaria morbidity and immunity among residents of villages with different Plasmodium falciparum transmission intensity in North-Eastern Tanzania. MalarJ *3*, 26.

Malaria Genomic Epidemiology, N., Band, G., Rockett, K.A., Spencer, C.C., and Kwiatkowski, D.P. (2015). A novel locus of resistance to severe malaria in a region of ancient balancing selection. Nature *526*, 253-257.

McCoy, A.J., Grosse-Kunstleve, R.W., Adams, P.D., Winn, M.D., Storoni, L.C., and Read, R.J. (2007). Phaser crystallographic software. J Appl Crystallogr *40*, 658-674.

Menschikowski, M., Hagelgans, A., Eisenhofer, G., and Siegert, G. (2009). Regulation of endothelial protein C receptor shedding by cytokines is mediated through differential activation of MAP kinase signaling pathways. Exp Cell Res *315*, 2673-2682.

Miotto, O., Amato, R., Ashley, E.A., MacInnis, B., Almagro-Garcia, J., Amaratunga, C., Lim, P., Mead, D., Oyola, S.O., Dhorda, M.*, et al.* (2015). Genetic architecture of artemisinin-resistant Plasmodium falciparum. Nat Genet *47*, 226-234.

Moussiliou, A., Alao, M.J., Denoeud-Ndam, L., Tahar, R., Ezimegnon, S., Sagbo, G., Amoussou, A., Luty, A.J.F., Deloron, P., and Ndam, N.T. (2015). High plasma levels of soluble endothelial protein C receptor are associated with increased mortality among children with cerebral malaria in Benin. JInfectDis *211*, 1484-1488.

Moxon, C.A., Heyderman, R.S., and Wassmer, S.C. (2009). Dysregulation of coagulation in cerebral malaria. Mol Biochem Parasitol *166*, 99-108.

Moxon, C.A., Wassmer, S.C., Milner, D.A., Jr., Chisala, N.V., Taylor, T.E., Seydel, K.B., Molyneux, M.E., Faragher, B., Esmon, C.T., Downey, C.*, et al.* (2013). Loss of endothelial protein C receptors links coagulation and inflammation to parasite sequestration in cerebral malaria in African children. Blood *122*, 842-851.

Murshudov, G.N., Skubak, P., Lebedev, A.A., Pannu, N.S., Steiner, R.A., Nicholls, R.A., Winn, M.D., Long, F., and Vagin, A.A. (2011). REFMAC5 for the refinement of macromolecular crystal structures. Acta Crystallogr D Biol Crystallogr *67*, 355-367.

Newbold, C., Warn, P., Black, G., Berendt, A., Craig, A., Snow, B., Msobo, M., Peshu, N., and Marsh, K. (1997). Receptor-specific adhesion and clinical disease in Plasmodium falciparum. AmJTropMedHyg *57*, 389-398.

Niang, M., Bei, A.K., Madnani, K.G., Pelly, S., Dankwa, S., Kanjee, U., Gunalan, K., Amaladoss, A., Yeo, K.P., Bob, N.S.*, et al.* (2014). STEVOR is a Plasmodium falciparum erythrocyte binding protein that mediates merozoite invasion and rosetting. Cell Host Microbe *16*, 81-93.

Nielsen, M.A., Staalsoe, T., Kurtzhals, J.A., Goka, B.Q., Dodoo, D., Alifrangis, M., Theander, T.G., Akanmori, B.D., and Hviid, L. (2002). *Plasmodium falciparum* variant surface antigen expression varies between isolates causing severe and nonsevere malaria and is modified by acquired immunity. JImmunol *168*, 3444-3450.

Nunes-Silva, S., Dechavanne, S., Moussiliou, A., Pstrag, N., Semblat, J.P., Gangnard, S., Tuikue-Ndam, N., Deleron, P., Chene, A., and Gamain, B. (2015). Beninese children with cerebral malaria do not develop humoral immunity against the IT4-VAR19-DC8 PfEMP1 variant linked to EPCR and brain endothelial binding. MalarJ *14*, 493.

Ochola, L.B., Siddondo, B.R., Ocholla, H., Nkya, S., Kimani, E.N., Williams, T.N., Makale, J.O., Liljander, A., Urban, B.C., Bull, P.C.*, et al.* (2011). Specific receptor usage in Plasmodium falciparum cytoadherence is associated with disease outcome. PLoSOne *6*, e14741.

Oleinikov, A.V., Amos, E., Frye, I.T., Rossnagle, E., Mutabingwa, T.K., Fried, M., and Duffy, P.E. (2009). High throughput functional assays of the variant antigen PfEMP1 reveal a single domain in the 3D7 *Plasmodium falciparum* genome that binds ICAM1 with high affinity and is targeted by naturally acquired neutralizing antibodies. PLoSPathog *5*, e1000386.

Pan, Y., Ma, B., and Nussinov, R. (2005). CD4 binding partially locks the bridging sheet in gp120 but leaves the beta2/3 strands flexible. J Mol Biol *350*, 514-527.

Petersen, J.E., Bouwens, E.A., Tamayo, I., Turner, L., Wang, C.W., Stins, M., Theander, T.G., Hermida, J., Mosnier, L.O., and Lavstsen, T. (2015). Protein C system defects inflicted by the malaria parasite protein PfEMP1 can be overcome by a soluble EPCR variant. Thromb Haemost *114*, 1038-1048.

Rask, T.S., Hansen, D.A., Theander, T.G., Gorm, P.A., and Lavstsen, T. (2010). Plasmodium falciparum erythrocyte membrane protein 1 diversity in seven genomes--divide and conquer. PLoSComputBiol *6*.

Robinson, B.A., Welch, T.L., and Smith, J.D. (2003). Widespread functional specialization of *Plasmodium falciparum* erythrocyte membrane protein 1 family members to bind CD36 analysed across a parasite genome. MolMicrobiol *47*, 1265-1278.

Rogerson, S.J., Tembenu, R., Dobano, C., Plitt, S., Taylor, T.E., and Molyneux, M.E. (1999). Cytoadherence characteristics of Plasmodium falciparum-infected erythrocytes from Malawian children with severe and uncomplicated malaria. AmJTropMedHyg *61*, 467-472.

Rowe, J.A., Moulds, J.M., Newbold, C.I., and Miller, L.H. (1997). P. falciparum rosetting mediated by a parasite-variant erythrocyte membrane protein and complement-receptor 1. Nature *388*, 292-295.

Schindelin, J., Arganda-Carreras, I., Frise, E., Kaynig, V., Longair, M., Pietzsch, T., Preibisch, S., Rueden, C., Saalfeld, S., Schmid, B.*, et al.* (2012). Fiji: an open-source platform for biological-image analysis. NatMethods *9*, 676-682.

Seydel, K.B., Kampondeni, S.D., Valim, C., Potchen, M.J., Milner, D.A., Muwalo, F.W., Birbeck, G.L., Bradley, W.G., Fox, L.L., Glover, S.J.*, et al.* (2015). Brain swelling and death in children with cerebral malaria. N Engl J Med *372*, 1126-1137.

Silamut, K., Phu, N.H., Whitty, C., Turner, G.D., Louwrier, K., Mai, N.T., Simpson, J.A., Hien, T.T., and White, N.J. (1999). A quantitative analysis of the microvascular sequestration of malaria parasites in the human brain. AmJPathol *155*, 395-410.

Snounou, G., Zhu, X., Siripoon, N., Jarra, W., Thaithong, S., Brown, K.N., and Viriyakosol, S. (1999). Biased distribution of *msp1* and *msp2* allelic variants in *Plasmodium falciparum* populations in Thailand. TransRSocTropMedHyg *93*, 369-374.

Stevenson, L., Laursen, E., Cowan, G.J., Bandoh, B., Barfod, L., Cavanagh, D.R., Andersen, G.R., and Hviid, L. (2015). alpha2-Macroglobulin Can Crosslink Multiple Plasmodium falciparum Erythrocyte Membrane Protein 1 (PfEMP1) Molecules and May Facilitate Adhesion of Parasitized Erythrocytes. PLoS Pathog *11*, e1005022.

Storm, J., and Craig, A.G. (2014). Pathogenesis of cerebral malaria--inflammation and cytoadherence. Front Cell Infect Microbiol *4*, 100.

Svergun, D. (1992). Determination of the regularization parameter in indirect-transform methods using perceptual criteria. Journal of Applied Crystallography *25*, 495-503.

Svergun, D., Barberato, C., and Koch, M.H.J. (1995). CRYSOL - a Program to Evaluate X-ray Solution Scattering of Biological Macromolecules from Atomic Coordinates. Journal of Applied Crystallography *28*, 768-773.

Tamura, K., Dudley, J., Nei, M., and Kumar, S. (2007). MEGA4: Molecular Evolutionary Genetics Analysis (MEGA) software version 4.0. Mol Biol Evol *24*, 1596-1599.

Theisen, M., Soe, S., Jessing, S.G., Okkels, L.M., Danielsen, S., Oeuvray, C., Druilhe, P., and Jepsen, S. (2000). Identification of a major B-cell epitope of the Plasmodium falciparum glutamate-rich protein (GLURP), targeted by human antibodies mediating parasite killing. Vaccine *19*, 204-212.

Turner, G.D., Morrison, H., Jones, M., Davis, T.M., Looareesuwan, S., Buley, I.D., Gatter, K.C., Newbold, C.I., Pukritayakamee, S., Nagachinta, B.*, et al.* (1994). An immunohistochemical study of the pathology of fatal malaria. Evidence for widespread endothelial activation and a potential role for intercellular adhesion molecule-1 in cerebral sequestration. AmJPathol *145*, 1057-1069.

Turner, L., Lavstsen, T., Berger, S.S., Wang, C.W., Petersen, J.E., Avril, M., Brazier, A.J., Freeth, J., Jespersen, J.S., Nielsen, M.A.*, et al.* (2013). Severe malaria is associated with parasite binding to endothelial protein C receptor. Nature *498*, 502-505.

Volkov, V.V., and Svergun, D.I. (2003). Uniqueness of ab initio shape determination in small-angle scattering. Journal of Applied Crystallography *36*, 860-864.

Whelan, S., and Goldman, N. (2001). A general empirical model of protein evolution derived from multiple protein families using a maximum-likelihood approach. Mol Biol Evol *18*, 691-699.

Winn, M.D., Ballard, C.C., Cowtan, K.D., Dodson, E.J., Emsley, P., Evans, P.R., Keegan, R.M., Krissinel, E.B., Leslie, A.G., McCoy, A.*, et al.* (2011). Overview of the CCP4 suite and current developments. Acta Crystallogr D Biol Crystallogr *67*, 235-242.
