# Supplementary material for: Structure-Guided Identification of a Family of Dual Receptor-Binding PfEMP1 that Is Associated with Cerebral Malaria
Source: Cell Host Microbe. 2017 Mar 8;21(3):403–14. doi: 10.1016/j.chom.2017.02.009 (PMC5374107; doi:10.1016/j.chom.2017.02.009)
Supplement: Document S2. Article plus Supplemental Information [file mmc4.pdf]

Article

Cell Host & Microbe

Structure-Guided Identification of a Family of Dual Receptor-Binding PfEMP1 that Is Associated with Cerebral Malaria

Graphical Abstract

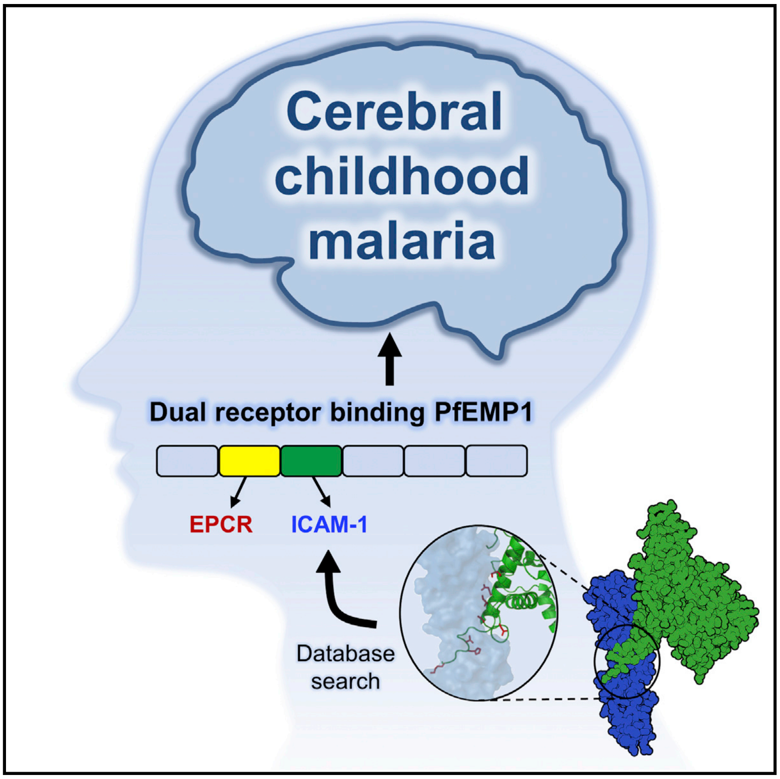

Highlights

- Structural basis for *P. falciparum* PfEMP1 binding to endothelial receptor ICAM-1defined
- A sequence motif derived from structure predicts group A PfEMP1 binding to ICAM-1
- These ICAM-1-binding PfEMP1s also all bind to endothelial protein C receptor (EPCR)
- Expression of dual ICAM-1- and EPCR-binding PfEMP1 is associated with cerebral malaria

Authors

Frank Lennartz, Yvonne Adams, Anja Bengtsson, ..., Lars Hviid, Matthew K. Higgins, Anja T.R. Jensen

Correspondence

matthew.higgins@bioch.ox.ac.uk (M.K.H.),
atrj@sund.ku.dk (A.T.R.J.)

In Brief

Plasmodium falciparum-infected erythrocytes display PfEMP1 proteins that bind various endothelial receptors, including ICAM-1. Lennartz et al. structurally characterize PfEMP1 binding to ICAM-1, allowing them to identify a PfEMP1 family that simultaneously binds to both ICAM-1 and EPCR. Dual-binding PfEMP1s display stronger endothelial adhesion and are associated with cerebral malaria.

Accession Numbers

KF984156
KJ866957
KJ866958
KJ866959
KM364031
KM364032
KM364033
KM364034
5MZA

Lennartz et al., 2017, Cell Host & Microbe *21*, 403–414
March 8, 2017 © 2017 The Author(s). Published by Elsevier Inc.
http://dx.doi.org/10.1016/j.chom.2017.02.009

CellPress

Structure-Guided Identification of a Family of Dual Receptor-Binding PfEMP1 that Is Associated with Cerebral Malaria

Frank Lennartz,[1,9] Yvonne Adams,[2,3,9] Anja Bengtsson,[2,3,9] Rebecca W. Olsen,[2,3] Louise Turner,[2,3] Nicaise T. Ndam,[4,5] Gertrude Ecklu-Mensah,[2,3,6] Azizath Moussiliou,[5] Michael F. Ofori,[6] Benoit Gamain,[7] John P. Lusingu,[8] Jens E.V. Petersen,[2,3] Christian W. Wang,[2,3] Sofia Nunes-Silva,[7] Jakob S. Jespersen,[2,3] Clinton K.Y. Lau,[1] Thor G. Theander,[2,3] Thomas Lavstsen,[2,3] Lars Hviid,[2,3] Matthew K. Higgins,[1,10,*] and Anja T.R. Jensen[2,3,*]
[1]Department of Biochemistry, University of Oxford, South Parks Road, OX1 3QU Oxford, UK
[2]Centre for Medical Parasitology, Department of Immunology and Microbiology (ISIM), Faculty of Health and Medical Sciences, University of Copenhagen, 1165 Copenhagen, Denmark
[3]Department of Infectious Diseases, Copenhagen University Hospital (Rigshospitalet), 2100 Copenhagen, Denmark
[4]Faculté de Pharmacie, Institut de Recherche pour le Développement (IRD), COMUE Sorbonne Paris Cité, 75013 Paris, France
[5]Faculté des Sciences de la Santé (FSS), Université d'Abomèy Calavi, 01 BP 526 Cotonou, Benin
[6]Department of Immunology, Noguchi Memorial Institute for Medical Research, University of Ghana, Legon, Ghana
[7]UMR_S1134, Université Sorbonne Paris Cité, Université Paris Diderot, Inserm, INTS, Unité Biologie Intégrée du Globule Rouge, Laboratoire d'Excellence GR-Ex, 75013 Paris, France
[8]National Institute for Medical Research, Tanga Centre, 11101 Dar es Salaam, Tanzania
[9]Co-first author
[10]Lead Contact
*Correspondence: matthew.higgins@bioch.ox.ac.uk (M.K.H.), atrj@sund.ku.dk (A.T.R.J.)
http://dx.doi.org/10.1016/j.chom.2017.02.009

SUMMARY

Cerebral malaria is a deadly outcome of infection by *Plasmodium falciparum*, occurring when parasite-infected erythrocytes accumulate in the brain. These erythrocytes display parasite proteins of the PfEMP1 family that bind various endothelial receptors. Despite the importance of cerebral malaria, a binding phenotype linked to its symptoms has not been identified. Here, we used structural biology to determine how a group of PfEMP1 proteins interacts with intercellular adhesion molecule 1 (ICAM-1), allowing us to predict binders from a specific sequence motif alone. Analysis of multiple *Plasmodium falciparum* genomes showed that ICAM-1-binding PfEMP1s also interact with endothelial protein C receptor (EPCR), allowing infected erythrocytes to synergistically bind both receptors. Expression of these PfEMP1s, predicted to bind both ICAM-1 and EPCR, is associated with increased risk of developing cerebral malaria. This study therefore reveals an important PfEMP1-binding phenotype that could be targeted as part of a strategy to prevent cerebral malaria.

INTRODUCTION

Malaria affects hundreds of millions of people each year. While most cases are not life threatening, a significant number of infections by *Plasmodium falciparum* result in severe malaria, manifested in one or more of three major syndromes: anemia, respiratory distress, and cerebral malaria (CM). These occur as parasites infect and divide within human erythrocytes. The infected erythrocytes adhere to blood vessels and tissue surfaces, allowing the parasite to avoid clearance by the spleen. In addition, organ-specific sequestration can have major consequences for development of specific malaria symptoms, particularly in CM, the most debilitating form of the disease. Here, infected erythrocytes accumulate in the brain, occluding blood flow, inducing inflammation, and leading to major neurological complications (Hviid and Jensen, 2015). Even with antimalarial treatment, mortality rates due to CM in children range between 15% and 20% (Dondorp et al., 2010; Seydel et al., 2015), and survivors of CM often suffer from a wide variety of long-lasting neurological damage, which can result in loss of motor function, impairment in learning and language capability, or an increased risk of epilepsy (Birbeck et al., 2010; Idro et al., 2005).

During blood stage infection, the parasite expresses members of different variant surface protein families. Of these, *Plasmodium falciparum* erythrocyte membrane proteins 1 (PfEMP1) are best understood. They are displayed on infected erythrocyte surfaces and tether the cells to various receptors. Each *Plasmodium falciparum* genome contains ~60 PfEMP1-encoding *var* genes, which can be classified according to their chromosomal context into groups A–E. They have large ectodomains containing multiple duffy binding-like (DBL) and cysteine-rich inter-domain region (CIDR) domains that can interact with specific human endothelial receptors. Sequence analysis allows the CIDR and DBL domains to be divided into general subgroups associated with specific binding phenotypes (Hviid and Jensen, 2015). In particular, subclasses of DBLβ domains found in group A, B, and C PfEMP1 bind ICAM-1 (Bengtsson et al., 2013; Howell et al., 2008; Janes et al., 2011); CIDRα1 domains from group A PfEMP1 bind endothelial protein C receptor (EPCR) (Lau et al., 2015; Turner et al., 2013);

This is an open access article under the CC BY license (http://creativecommons.org/licenses/by/4.0/).

and group B and C PfEMP1 contain CIDRα2–6 domains, which bind CD36 (Hsieh et al., 2016; Robinson et al., 2003).

With a number of receptors available to bind to PfEMP1, a major goal has been to determine whether PfEMP1s with specific receptor-binding phenotypes are associated with cerebral and other forms of severe malaria. These studies mostly focused on children, as adults from malaria endemic areas have a lower risk of death from complications during infection. Early studies showed that parasites that express group A PfEMP1 or those that form rosettes are linked with severe malaria (Doumbo et al., 2009; Jensen et al., 2004). The search was further focused with the discovery that severe malaria is associated with a subset of group A and B/A PfEMP1s (Avril et al., 2012; Claessens et al., 2012; Lavstsen et al., 2012), which were later shown to bind to EPCR (Turner et al., 2013). This demonstrated a link between severe malaria and expression of EPCR-binding PfEMP1 (Bernabeu et al., 2016; Jespersen et al., 2016; Turner et al., 2013).

While these studies established associations between severe malaria and particular groups of PfEMP1, no connections have been identified to any specific individual severe malaria syndrome. In particular, attempts to link CM to expression of certain groups of PfEMP1 remain inconclusive. Some studies have correlated cerebral disease with ICAM-1 binding (Ochola et al., 2011; Silamut et al., 1999; Turner et al., 1994), but others found no such link (Newbold et al., 1997; Rogerson et al., 1999). Indeed, ICAM-1-binding DBLβ domains occur in B- and C-type PfEMP1s that are associated with uncomplicated malaria as well as A-type PfEMP1s associated with severe disease, suggesting that ICAM-1 binding alone is not a driver of CM (Bengtsson et al., 2013; Howell et al., 2008; Janes et al., 2011).

A significant obstacle to associating specific adhesion phenotypes with disease outcomes has been the inability to directly predict, using sequence information, adhesion traits of PfEMP1 expressed in patients. While EPCR- and CD36-binding CIDR domains can be predicted from their sequences (Hsieh et al., 2016; Robinson et al., 2003; Turner et al., 2013), ICAM-1-binding domains cannot. We therefore aimed to understand the molecular basis for ICAM-1 binding by A-type PfEMP1, to define a motif allowing the identification of ICAM-1-binding DBLβ domains from sequence alone, and to determine whether the expression of these ICAM-1-binding domains is associated with the development of CM.

RESULTS

The Structural Basis for ICAM-1 Binding by PfEMP1

In the absence of a structure of a DBLβ domain bound to ICAM-1, we purified complexes of a diverse set of DBLβ domains from group A PfEMP1 bound to the N-terminal two domains of ICAM-1 (ICAM-1^{D1D2}), increasing the likelihood of obtaining well-diffracting crystals. Of these, a complex of the PF11_0521 (PlasmoDB: PF3D7_1150400) DBLβ3_D4 domain and ICAM-1^{D1D2} formed crystals that diffracted to 2.8 Å resolution (Table S1). We used molecular replacement, with previous structures of ICAM-1 domains and a model derived from the varO DBLα domain as search models, to determine the structure (Figures 1A and 1B). We also used small-angle X-ray scattering to confirm that the arrangement of the complex was the same in solution as that observed in the crystals, and that it further matched the arrangement of a complex of the DBLβ domain bound to full-length ICAM-1^{D1D5} (Figure S1).

The PF11_0521 DBLβ3_D4 domain adopts the classical DBL domain fold, consisting of a core of α helices, decorated by numerous loops (Figures 1, S2A, and S2B). In contrast to previously characterized DBL domains, an extended α helix and a glycine and proline-rich linker form a unique feature that protrudes from the domain and generates part of the ICAM-1 interaction site (Figures 1, S2A, and S2B). Indeed, a chimeric protein containing this region of an ICAM-1-binding domain, transplanted to a related, non-binding DBLβ domain, produced an ICAM-1-binding domain (Figure 2A), and antibodies targeting this region (either purified from peptide-immunized rat or from human plasma) prevented infected erythrocytes from binding to ICAM-1 (Figures 2B and 2C).

The DBLβ domain interacts with ICAM-1 through a complex, elongated binding interface that contains three distinct subsites with a total surface area of 1,144 Å^2 (Figure 1C). Site 1 is formed from a patch of hydrophobic residues along one side of the extended α helix of DBLβ that interacts with a corresponding hydrophobic patch on the surface of ICAM-1 D1. Site 2 is formed by the subsequent linker of the DBLβ domain and forms hydrogen bonds with both ICAM-1 D1 and D2 domains. Site 3 is formed from part of a long loop that protrudes from the DBLβ domain and also contacts both ICAM-1 D1 and D2. The parts of this loop not in contact with ICAM-1 are not observed in the electron density, indicating flexibility (Figures 1B and S2C). This loop is indeed predicted to be intrinsically disordered (Figure S2D), but becomes partially ordered when bound to ICAM-1. Such a flexible region of a protein, which forms part of, or is immediately adjacent to, its ligand-binding site, is an immune evasion strategy used by surface proteins from other pathogens (Kim et al., 2004; Kwong et al., 1998), but had not been previously observed in any PfEMP1 domain bound to its receptor.

A Sequence Motif Allows for the Prediction of ICAM-1 Binding A-type PfEMP1

We next aimed to use the crystal structure to identify key molecular determinants used by the DBLβ domains for ICAM-1 binding, and to thereby identify a sequence motif that can be used to predict these ICAM-1 binders. For this, we mutated residues in the DBLβ domain that either directly contact ICAM-1 or are responsible for the unusual architecture of the binding site (Figure 2D; Table S2). By testing the eight residues that directly interact with ICAM-1 (Figure S3; Table S2), we found that each of the three subsites contains a critical residue that, when mutated, disrupts ICAM-1 binding (Figure 2E; Table S3). In addition, certain structural residues, in particular glycines and prolines, are essential for ICAM-1 binding as they allow sharp backbone bends that correctly present binding residues (Figures 2 and S3; Table S3). To ensure that the observed effects were not due to disruption in the overall fold of the DBL domain, all mutants that showed an effect on ICAM-1 binding were tested by circular dichroism spectroscopy and found to be indistinguishable from the wild-type domain (Figure S4).

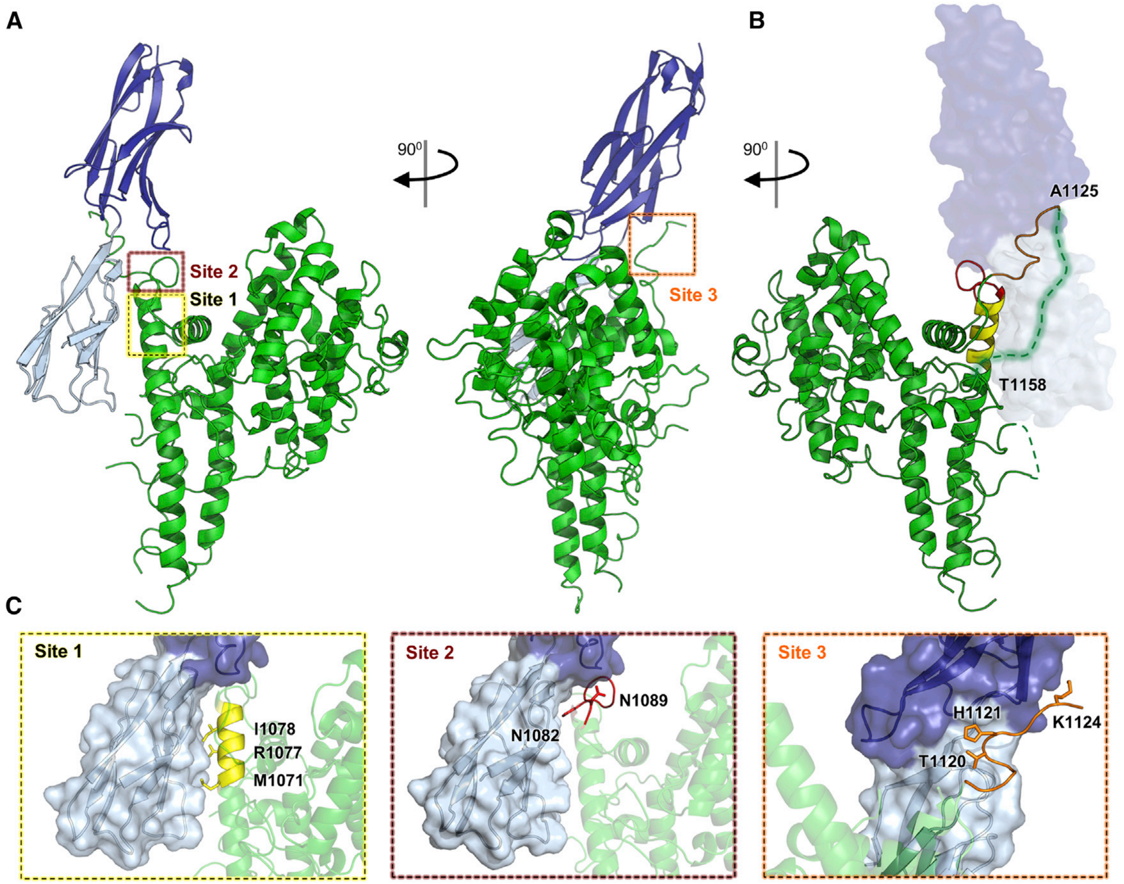

Figure 1. The Structural Basis for ICAM-1 Binding by Group A PfEMP1s

(A) Front and side views of the PF11_0521 DBLβ_D4 domain (green) bound to ICAM-1^{D1D2} (D1, light blue; D2, dark blue). Dashed boxes highlight three sites that make direct contact with ICAM-1.

(B) Back view of PF11_0521 DBLβ_D4 domain (green) bound to ICAM-1^{D1D2}. Dashed lines represent residues not visible in the electron density map with the disordered regions near site 3 (orange) of the ICAM-1-binding site highlighted.

(C) Three distinct sites in the PF11_0521 DBLβ_D4 domain directly interact with ICAM-1 with residues that mediate this interaction indicated.

See also Figures S1 and S2 and Tables S1 and S2.

Based on this analysis, coupled with sequence analysis of previously identified A-type ICAM-1 binding domains (Bengtsson et al., 2013), we defined a sequence motif (I[V/L]x_3N[E]GG[P/A]xYx_{27}GPPx_3H) that contains the determinants for ICAM-1 binding by these group A PfEMP1s. This motif was used to search sequence databases, identifying a total of 145 ICAM-1-binding DBL domains (mostly A-type PfEMP1s with a few B/A-type PfEMP1s) that contain the motif and are therefore predicted to bind to ICAM-1 (Table S4). To test these predictions, we used ELISA to analyze the interaction between ICAM-1 and 16 motif-containing DBLβ domains, selected to represent sequence diversity (Table S4). All 16 DBLβ domains with the motif bound ICAM-1, while 10 group A DBLβ domains without the motif did not (Figures 2F and 3A; Table S4). In sequence analysis of DBLβ domains (n = 55) with known ICAM-1-binding properties and DBLβ S3 sequences (n = 1,823) from whole-genome studies, the ICAM-1-binding group A and B/A domains with the motif clustered separately from other domains, including group B and C ICAM-1-binding DBLβ domains lacking the motif (Figures 3 and S5). Taken together, this suggests at least two groups of ICAM-1-binding PfEMP1s with different evolutionary histories and that the majority of ICAM-1-binding group A and B/A PfEMP1s can be predicted from the presence of a highly conserved binding site (Figures 2G and 2H).

Parasites Expressing ICAM-1-Binding DBLβ Domains Elicit Inhibitory Antibodies and Are Associated with CM

The high sequence identity of DBLβ domains containing the motif (Table S4) encouraged us to determine whether these domains might induce cross-reactive antibodies during natural infections. We therefore screened samples from Liberian adults for the presence of immune plasma that inhibited DBLβ domains from binding to ICAM-1. Plasma IgG from a pool of these inhibitory samples was then affinity purified separately on three different DBLβ domains containing the motif (Dd2var32, KM364031, and PFD1235w [PlasmoDB: PF3D7_0425800] DBLβ_D4) and tested for the ability to inhibit ICAM-1 binding of DBLβ domains (Figure 4A). The IgG showed the potential to broadly inhibit ICAM-1 binding by a panel of motif-containing DBLβ domains from group A PfEMP1, but did not prevent ICAM-1 binding by

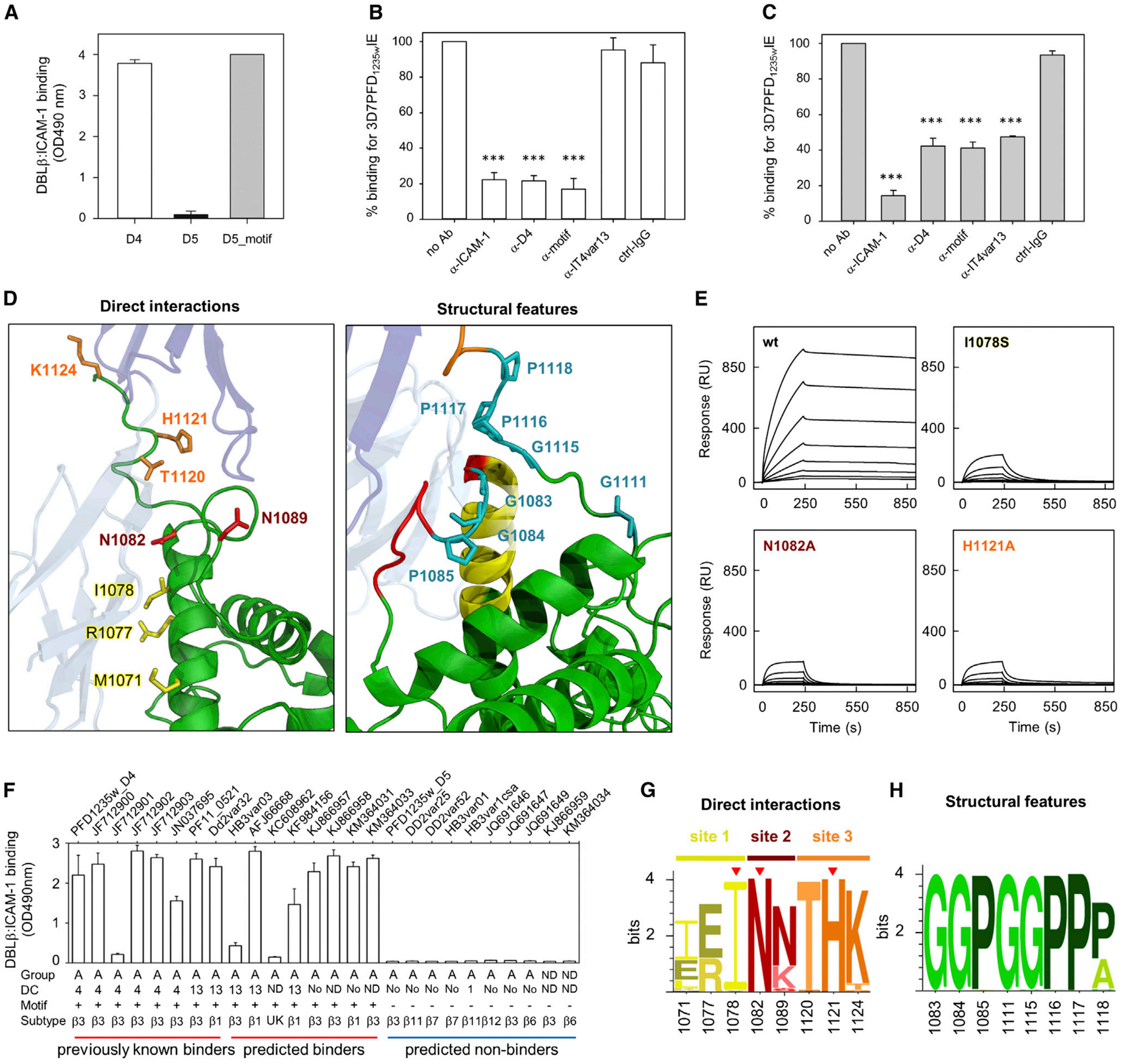

Figure 2. Structure-Guided Identification of a Motif that Predicts ICAM-1 Binding

(A) ICAM-1 binding (ELISA OD 490 nm; ± SD) of recombinant 3D7 PFD1235w DBLβ3_D4, PFD1235w DBLβ3_D5, and a chimeric DBLβ3_D5 containing the ICAM-1-binding motif region of DBLβ3_D4 (D5_motif).

(B and C) Inhibition of 3D7 PFD1235W infected erythrocyte adhering to ICAM-1 under flow in the presence of anti-ICAM-1 Abs, affinity-purified anti-PFD1235w DBLβ3_D4 (anti-D4) IgG, anti-PFD1235w_motif IgG, anti-ITVAR13, and control IgG. IgG antibodies affinity purified from (B) rat anti-serum and (C) human serum. ± SD of a minimum of three independent experiments done in triplicate. Data were analyzed using one-way ANOVA. Asterisks indicate significance ($p = 0.0004$).

(D) The three sites within the PF11_0521 DBLβ3_D4-binding site, indicating residues that directly contact ICAM-1 (site 1, yellow; site 2, red; site 3, orange). Structural residues important for positioning of interacting residues are highlighted in teal.

(E) Surface plasmon resonance curves for injection of 2-fold dilution series of DBLβ wild-type and binding site mutants over ICAM-1^{D1D5}.

(F) ICAM-1 binding (ELISA OD 490 nm; ± SD three replicates) of 26 recombinant group A DBLβ domains (4 DBLβ1, 14 DBLβ3, 2 DBLβ6, 2 DBLβ7, 2 DBLβ11, 1 DBLβ12, and 1 DBLβ unknown sub-class), confirming prediction of binding domains. "DC" indicates in which domain cassette the domain is found. "ND" indicates that the DC type is unknown as only the DBLβ sequence is available. "No" indicates that the domain is not part of a known DC. Presence of DC13 prior to DBLβ is indicated.

(G and H) Sequence logo showing conservation of (G) residues that contact ICAM-1 and (H) residues important for the unusual architecture of the ICAM-1-binding site, based on 145 DBLβ domains predicted to bind ICAM-1. Red triangles, residues critical for direct interaction with ICAM-1.

See also Figures S3–S5 and Tables S2, S3, and S4.

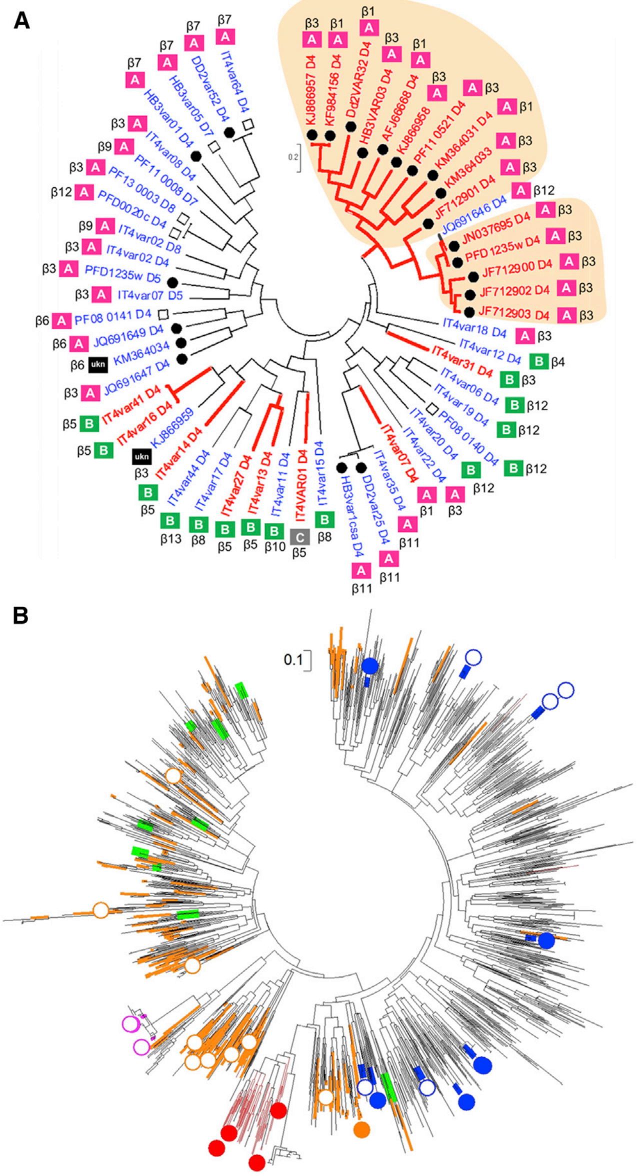

Figure 3. Phylogeny of ICAM-1- and Non-binding DBLβ Domains

(A) Maximum likelihood tree of 55 complete DBLβ domains. The tree is drawn to scale, with branch lengths measured in substitutions per site. Red, ICAM-1 binders; blue, ICAM-1 non-binders (Avril et al., 2016; Bengtsson et al., 2013; Janes et al., 2011; Lau et al., 2015). Filled circles, DBLβ domains tested in this study; open squares, DBLβ domains tested, but data not shown. UKN indicates unknown group ID. Shaded areas indicate DBLβ with the ICAM-1 motif.

(B) Maximum likelihood tree of 1,823 complete DBLβ S3 sequences from the seven published genomes and 226 annotated Sanger genomes. Filled circles, DBLβ experimentally shown as ICAM-1 binders. Open circles, DBLβ experimentally shown as ICAM-1 non-binders. Colors indicate the CIDR domain N-terminal to DBLβ: red, EPCR binders with ICAM-1-binding motif; orange, EPCR binders with no ICAM-1-binding motif; green, non-EPCR binders (group A); blue, CD36 binders; magenta, VAR1.

See also Figure S5 and Table S4.

a DBLβ domain from a group B PfEMP1 (IT4VAR13). In contrast, IgG that was affinity purified on a closely related non-ICAM-1-binding DBLβ domain that lacks the motif (PFD1235w DBLβ_D5) did not significantly inhibit binding of any DBLβ domain tested (Figure 4A). Therefore, plasma from adults in a malaria endemic region contains IgG that shows the potential to broadly inhibit ICAM-1 binding of motif-containing PfEMP1.

We also tested whether children develop antibodies that specifically target the ICAM-1 binding site of the motif-containing DBLβ domains. To this end, we screened plasma samples from *Plasmodium falciparum*-exposed non-hospitalized Tanzanian children living in an area of high malaria transmission against a peptide containing the motif. A high proportion (69 of 76) of samples contained motif-reactive antibodies, and the DBLβ:ICAM-1-binding inhibition was significantly higher in plasma with high reactivity (ELISA optical density [OD] > 1) against the motif when compared with those with low motif reactivity (ELISA OD < 1) (Figure 4B).

Next, to correlate the expression of the group A ICAM-1-binding PfEMP1 with disease outcomes, we used the sequence motif to design primers to selectively amplify motif-encoding *var* gene transcripts. We used these to assess transcript levels in children with CM and severe malarial anemia (SA), and in other pediatric malaria patients (other malaria, OM; non-CM and non-SA) (Figure 4C; Table S5). The abundance of *var* gene transcripts was calculated relative to the transcript of an endogenous control gene and was translated into transcript units (Lavstsen et al., 2012). This analysis showed higher transcription of motif-encoding, ICAM-1-binding PfEMP1 in children with CM than in children with SA or in those admitted to hospital with malaria without these signs of severity (OM). Due to the challenges in acquiring a sufficiently large number of samples from single locations, patients were recruited at different sites and logistic regression modeling was used to correct for this. Comparison of the three groups showed that levels of transcripts encoding ICAM-1-binding PfEMP1 were significantly associated with risk of CM ($p = 0.02$) when comparing CM patients to malaria patients without CM (SA and OM). In contrast, transcript levels reported by primers targeting loci encoding EPCR-binding PfEMP1 (Mkumbaye et al., 2017) was not higher in CM patients compared to SA plus OM patients ($p = 0.751$). These data suggest a direct link between CM and expression of this particular subset of ICAM-1-binding PfEMP1s (Figure 4C).

Motif-Containing ICAM-1-Binding DBLβ Domains Are Associated with EPCR-Binding Domains, Allowing Simultaneous Dual Receptor Binding

The correlation between CM and expression of group A PfEMP1 containing the conserved ICAM-1-binding site raised the question of why ICAM-1 binding was not unambiguously associated with CM in previous studies. With ICAM-1 binding alone unlikely to be the driver of cerebral disease, we therefore examined the broader context of the motif-containing ICAM-1-binding domains. We made the striking observation that all tested PfEMP1s containing these domains also contain a neighboring EPCR-binding CIDRα1 domain (Table S4; Figures 3B and S5). These PfEMP1s form a separate cluster from PfEMP1s that contain an EPCR-binding domain, but no motif containing ICAM-1-binding domain (Figures 3B and S5), and we find that 14% of all EPCR binders have such a motif-containing ICAM-1-binding domain. In contrast to this, B- and C-type ICAM-1-binding PfEMP1s are frequently associated with CIDRα domains that

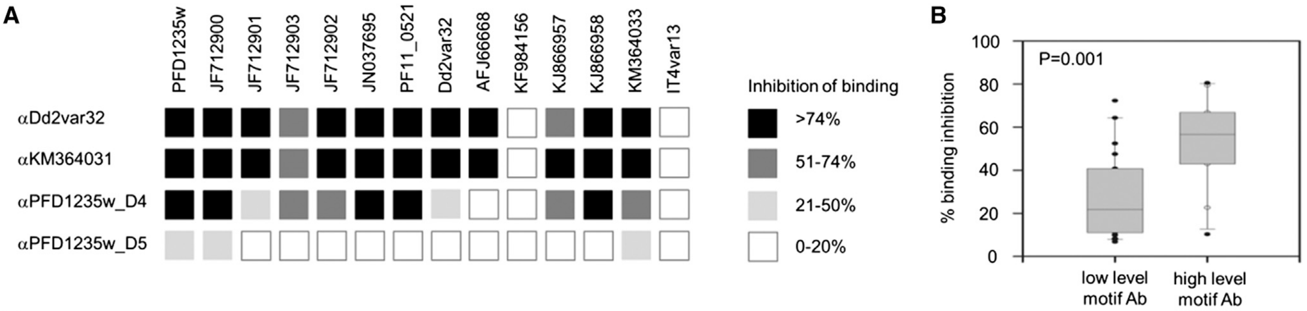

C

Predicted binding phenotype	Cerebral malaria (CM) *(n= 53)*	Severe anemia (SA) *(n= 27)*	Other malaria (OM) *(n= 36)*	CM vs. SA+OM, *P-value*
ICAM-1 binding Gr A DBLβ	7.5 [1.4; 32.9]	3.0 [1.0; 8.9]	2.4 [1; 12.3]	0.020
EPCR binding CIDRα	82.7 [36.4; 171.3]	46.4 [22.7; 126.2]	77.0 [29.9; 154.3]	0.751

Figure 4. The Conserved ICAM-1 Binding Site Is Recognized by Patient Plasma and Linked to CM

(A) The inhibition of DBLβ domains binding to ICAM-1 by IgG from a plasma pool from Liberian *P. falciparum*-exposed adults purified on ICAM-1-binding DBLβ_D4 domain from Dd2var32, KM364031, and PFD1235w or on a closely related, but non-ICAM-1-binding DBLβ_D5 domain from PFD1235w. ICAM-1 inhibitory capacity is >74% (black), 51%–74% (dark gray), 21%–50% (light gray), and 0%–20% (white).

(B) ICAM-1-binding inhibition by plasma with low (ELISA OD < 1) and high (ELISA OD > 1) anti-motif-DBLβ IgG in *P. falciparum*-exposed Tanzanian children (1–17 years) (Lusingu et al., 2004). Boxplot with median. Whiskers, 5% and 95% percentiles.

(C) Transcript levels (in arbitrary transcription units, Tu) of *var* gene subtypes in Ghanaian, Tanzanian, and Beninese children hospitalized with malaria, shown with medians and 25% and 75% percentiles.

See also Table S5.

interact with CD36 and are distributed across the phylogenetic tree (Figure 3B; Table S4). This suggests that the group A ICAM-1-binding PfEMP1s are a subset of the ICAM-1-binding PfEMP1s, separate from group B and C PfEMP1s, and that they also have the capacity to bind to EPCR.

The link between the presence of both ICAM-1- and EPCR-binding domains within a single PfEMP1 raised the intriguing possibility that these PfEMP1s might simultaneously engage both receptors. A few PfEMP1s have previously been identified that contain both EPCR-binding CIDRα domains and ICAM-1-binding DBLβ domains (Avril et al., 2016; Oleinikov et al., 2009; Turner et al., 2013). However, it was unknown whether they could interact with both receptors simultaneously, and whether such dual binding would have consequences for infected erythrocyte adhesion. We therefore produced a set of three different recombinant PfEMP1s and used surface plasmon resonance to test their potential for binding to the two receptors simultaneously. Indeed, we found that protein constructs containing the ICAM-1- and EPCR-binding domains of these PfEMP1s all bound simultaneously to ICAM-1 and EPCR with nanomolar affinity (Figures 5 and S6; Table S6).

Next, we examined the effect of dual binding to ICAM-1 and EPCR in the context of infected erythrocytes. For this, erythrocytes infected with parasites expressing PfEMP1 (PFD1235w or HB3VAR03) predicted to bind both receptors were selected in vitro (Figure S7) and binding to both receptors was tested under flow conditions using recombinant receptors (Figures 6A–6C) and in an immunofluorescence assay (Figures 6G–6I). These studies were performed at a range of flow rates from 0.5 to 5.0 dyn/cm^2, chosen to induce the most physiologically relevant shear stresses. Infected erythrocytes were capable of attaching between 0.5 and 1.0 dyn/cm^2 in the presence of either recombinant receptor alone. When ICAM-1 and EPCR were simultaneously available at comparable molar concentrations, erythrocytes infected with parasites expressing dual-binding PfEMP1 demonstrated an increased ability to bind at a higher shear stress, i.e., 2 dyn/cm^2, compared to when either receptor was available alone, though this adhesion did not exceed that measured at 1 dyn/cm^2 (Figures 6A and 6B; Table S7A). In contrast, erythrocytes infected with parasites known to bind ICAM-1 and CD36 (IT4VAR13; Figures S7E and S7F) showed increased adhesion in the presence of both ICAM-1 and CD36 compared to CD36 binding alone at lower shear stress, but did not confer the ability to withstand higher shear stresses, and the adhesion decreased in response to increasing shear stress (Figure 6C). The final parasite, known to bind to PECAM-1 (PF11_0008 [PlasmoDB: PF3D7_1100200]; Figures S7G and S7H), failed to bind any of the receptors tested (Figure S7L).

To further test the physiological relevance of our observations, we assessed the binding of infected erythrocytes to HBMEC with and without tumor necrosis factor α (TNFα) treatment (Figures 6D–6F and S7I–S7K). To determine the contribution of individual receptors to binding, antibodies against either ICAM-1 or EPCR alone or a combination of both were used. With HB3VAR03, at 0.5 dyn/cm^2 the greatest inhibition was observed on cells treated with both anti-receptor antibodies simultaneously (80%, $p = 0.0361$; Figure 6D), while HBMEC treated with either anti-ICAM-1 or anti-EPCR antibodies demonstrated a non-significant reduction in adhesion (Table S7B). A similar effect was observed at 0.75 dyn/cm^2. With increasing shear stress,

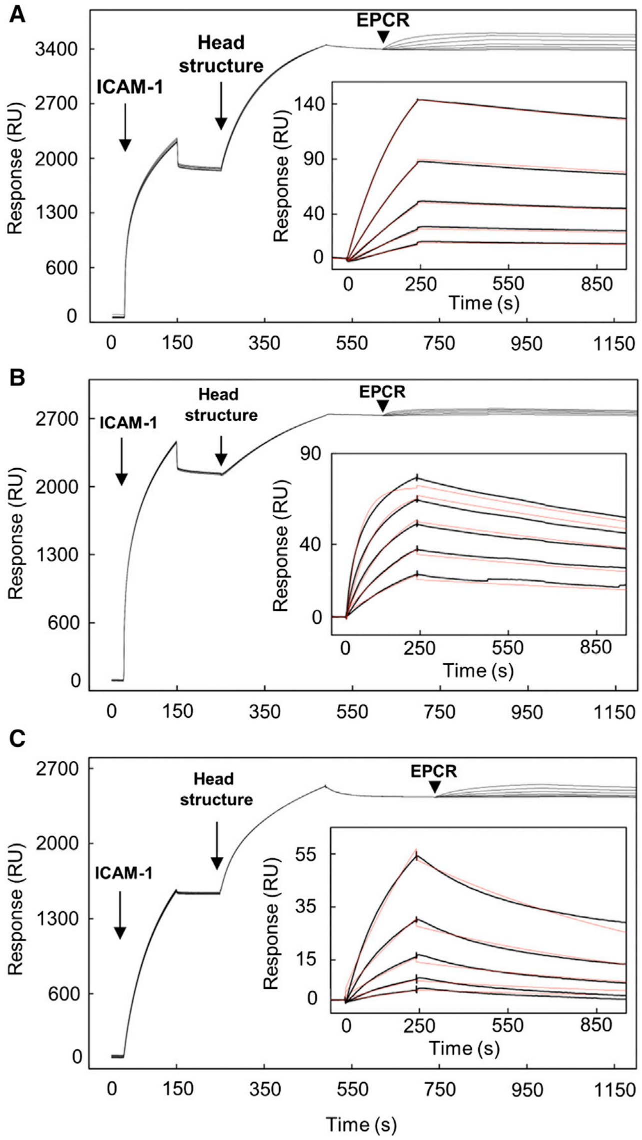

Figure 5. PfEMP1 Can Simultaneously Bind to Both ICAM-1 and EPCR

ICAM-1^{D1D5} was immobilized at fixed concentration, followed by injection of the respective head structure, (A) PF11_0521 DBLα1.7-CIDRα1.4-DBLβ3, (B) Dd2var32 DBLα1.7-CIDRα1.4-DBLβ3, and (C) PFD1235w CIDRα1.6-DBLβ3, also at fixed concentration. A 2-fold dilution series of EPCR was injected over the resulting ICAM-1::head structure complex. Arrows show start points of protein injection. The insets show data fitted to a one-site kinetic model for binding of EPCR to the respective ICAM-1::head structure complex. Data (black lines) are modeled to a one-site model (red lines). See also Figure S6 and Table S6.

the activity of single-antibody treatment was enhanced, and at 1 dyn/cm^2 this adhesion inhibition was significant for each individual anti-receptor antibody (Figure 6D; Table S7B), showing that the ability of HB3VAR03 to bind to both receptors is important at higher shear stresses. In comparison, 3D7PFD1235w infected erythrocytes were significantly inhibited by the presence of either antibody at all shear stresses tested (Figure 6E; Table S7B). As expected, IT4VAR13 only bound ICAM-1 and not EPCR expressed on the surface of HBMEC (Figure 6F). When the same experiments were conducted on activated cells, all three parasite lines showed increased reliance on ICAM-1 for adhesion, with HB3VAR03- and 3D7PFD1235w-infected erythrocytes switching to a single-receptor phenotype due to downregulation of EPCR expression upon cell activation by TNFα (Menschikowski et al., 2009) (Figures S7I–S7K). In summary, the ability to bind to both ICAM-1 and EPCR gives parasites significantly greater versatility, allowing for an increased capacity to adhere at physiological higher shear stresses, and enables them to continue to adhere despite changes in receptor expression upon cell activation.

Association of ICAM-1 and EPCR Dual-Binding PfEMP1 with the Development of CM

Our ability to predict dual-binding PfEMP1 from sequence next allowed us to assess the link between the presence of parasites expressing such PfEMP1 and the development of different severe malaria syndromes in patient isolates, and to compare this with the disease outcomes associated with PfEMP1s that bind EPCR alone. For this, we analyzed a dataset containing the full sequences of the six most abundant PfEMP1 transcripts expressed in 45 children hospitalized with malaria (on average corresponding to 75% of total *var* transcripts; see Supplemental Experimental Procedures), the biggest available dataset of its kind (Jespersen et al., 2016). These children presented with CM or SA, or were admitted to hospital with malaria, but without these signs of severity, and were immediately treated with antimalarial drugs (Table S5). We identified dual-binding PfEMP1 from the sequence data by presence of a DBLβ domain containing the group A ICAM-1-binding motif adjacent to an EPCR-binding CIDRα1 domain. EPCR-binding PfEMP1s were identified by the presence of CIDRα1 domains alone. In this dataset, parasites expressing PfEMP1 predicted to bind EPCR, but not ICAM-1, were more likely to be found in severe malaria cases (CM plus SA) ($p = 0.040$) (Figure 7B). However, there was no association between EPCR binding and either of the specific severe malaria syndromes (with $p = 1.000$ for comparison of CM with all other types of malaria). In contrast, parasites expressing dual ICAM-1- and EPCR-binding PfEMP1 were specifically ($p = 0.016$) associated with CM, when compared with non-cerebral disease (SA plus OM) (Figure 7A). These data support our finding that the expression of ICAM-1-binding DBLβ domains containing the motif, which to date has been 100% associated with dual ICAM-1- and EPCR-binding PfEMP1 (Figures 3 and S5; Table S4), is associated with the development of cerebral symptoms (Figures 4C and 7). Therefore, while expression of PfEMP1s that bind to EPCR alone is not linked to any specific severe malaria syndrome, the presence of dual-binding motif-containing PfEMP1s is associated with the development of CM.

DISCUSSION

A major challenge in associating malaria disease phenotypes to the expression of PfEMP1s with specific binding properties has been the ability to predict ligand-binding capability from sequence alone. In this study, we used structural studies to define a sequence motif that allowed the identification of a large set of

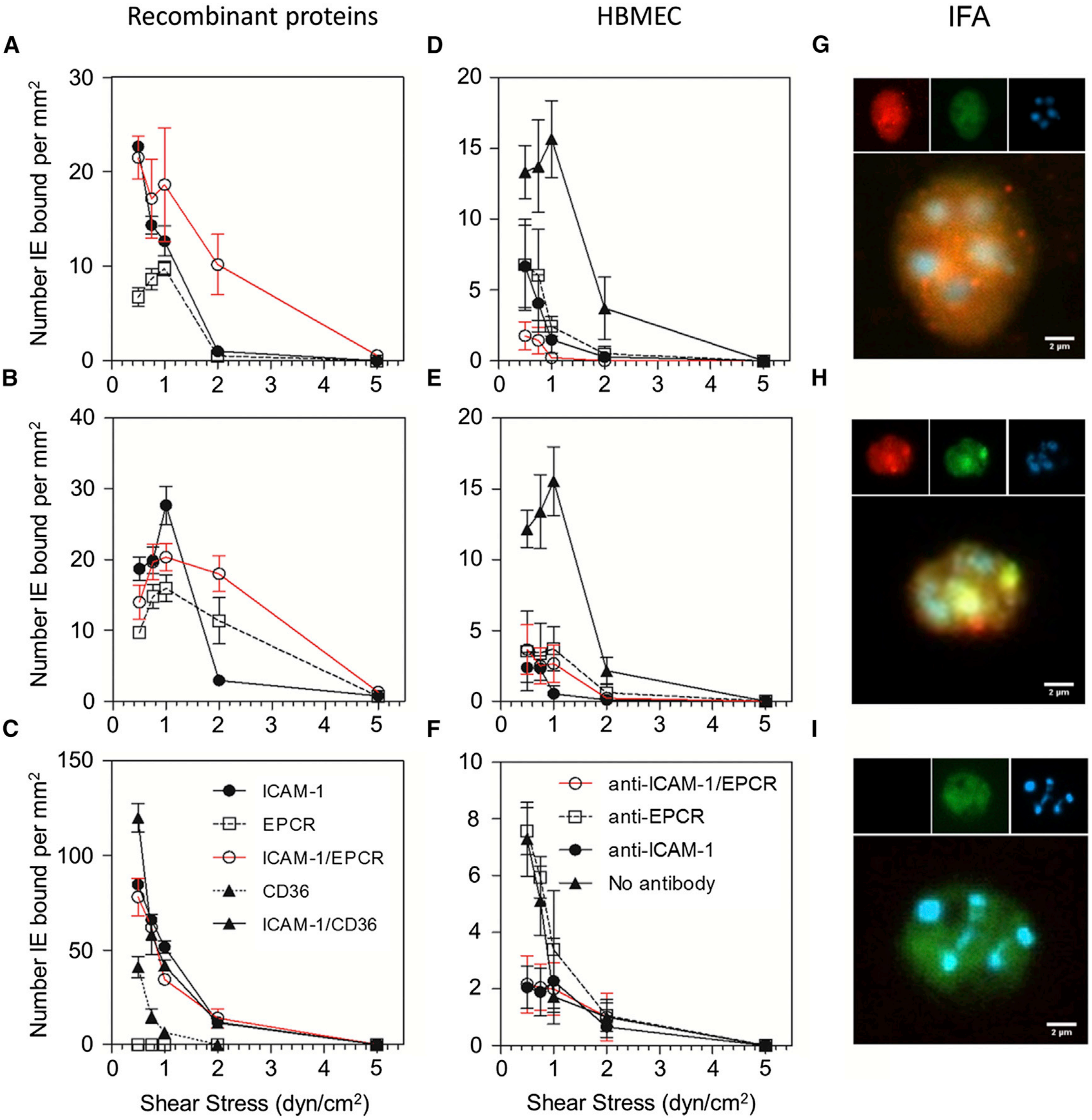

Figure 6. Binding to ICAM-1 and EPCR Increases Adhesion of Infected Erythrocytes

(A–F) Erythrocytes infected with (A and D) HB3VAR03 (predicted ICAM-1 and EPCR binder), (B and E) 3D7PFD1235w (predicted ICAM-1 and EPCR binder), and (C and F) IT4VAR13 (ICAM-1 and CD36 binder) binding to (A–C) receptor-coated microslides under flow conditions using recombinant ICAM-1, rEPCR, rICAM-1, and rEPCR; rCD36; or rICAM-1 and rCD36, and to (D–F) resting human brain microvascular endothelial cells (HBMEC). To demonstrate specific adhesion, channels coated with HBMEC were pre-incubated with anti-ICAM-1 (40 μg/mL), anti-EPCR (10 μg/mL), or anti-ICAM1 and EPCR combined (40 and 10 μg/mL, respectively). Flow experiments were done in parallel with the same conditions for immobilizing proteins or cell-coated chips. Values are bound infected erythrocyte per mm^2 ± SEM. Data were also analyzed by two-way ANOVA with Tukey's multiple comparison (Tables S7A and S7B).

(G–I) Immunofluorescence images of erythrocytes infected with parasite strains (G) HB3VAR03, (H) 3D7PFD1235w, and (I) IT4VAR13. Complexes of ICAM-1 or EPCR were added to measure binding by immunofluorescence. Main panels show overlays of ICAM-1 (green), EPCR (red), and nuclear (blue) staining. Inserts show single channels. Scale bar, 2 μm.

See also Figure S7 and Table S7.

ICAM-1-binding group A PfEMP1s from sequence. We also show that these ICAM-1-binding PfEMP1s all contain an additional EPCR-binding CIDRα1 domain and that this allows binding to both ligands. Finally, we show that the presence of parasites expressing PfEMP1s that bind to both ICAM-1 and EPCR is associated with increased risk of developing cerebral symptoms to *P. falciparum* infections.

This link between dual binding and CM is supported by two independent studies (Figures 4C and 7). However, while significant, it is not absolute. This may partly be because the data show a snapshot of the parasites present in the peripheral blood of the children at the time of hospital admission, and the children involved were treated with antimalarial medication immediately upon diagnosis, preventing further development of the disease.

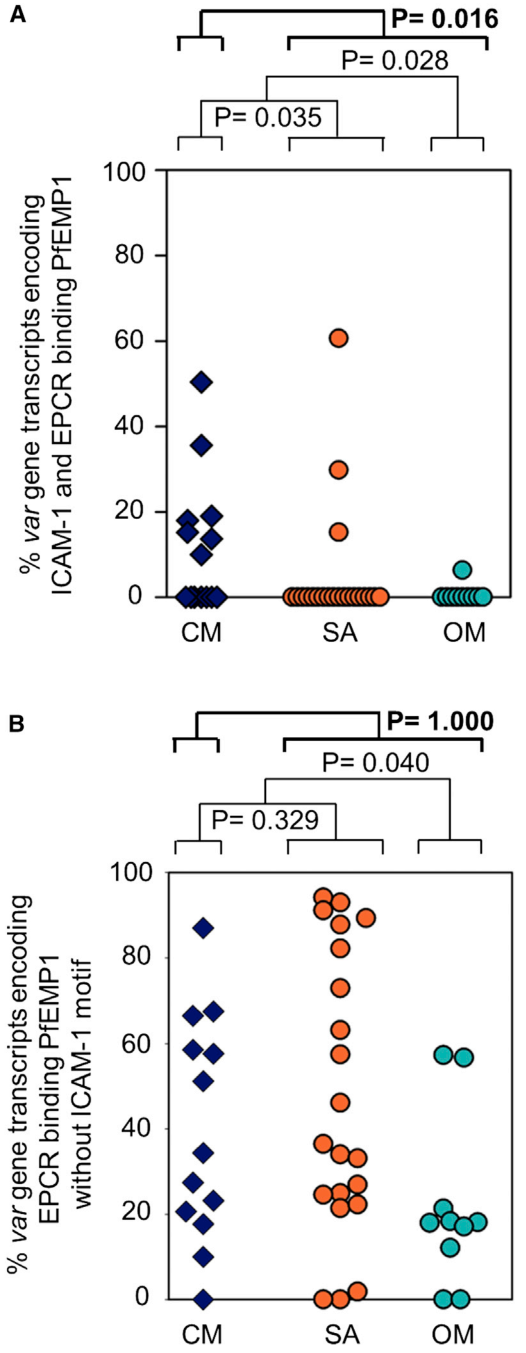

Figure 7. Dual-Binding PfEMP1s Correlate with CM

Percentages of specific *var* gene transcripts in infected erythrocytes from 45 hospitalized Tanzanian children.

(A) Percentage of *var* gene transcripts encoding PfEMP1s predicted to bind both ICAM-1 and EPCR.

(B) Percentage of transcripts that encode PfEMP1 predicted to bind EPCR and not containing the ICAM-1-binding motif. CM, cerebral malaria; SA, severe anemia; OM, children without the signs of severity found in the other groups. See also Table S5.

For example, in the analysis of whole PfEMP1 sequences (Figure 7), we cannot exclude the possibility that the small number of patients (3/21) expressing dual-binding PfEMP1 and presenting with SA, but not CM, may have developed cerebral symptoms if left untreated, causing the association detected here to be an underestimate. There are also risks in overestimating the link between CM and expression of this group of PfEMP1s due to limitations in the sample size available for each of these studies. However, this is mitigated by the fact that we observe the same association between dual-binding PfEMP1s and CM in two independent studies with samples from different patient populations from three African countries (Figures 4C and 7), suggesting that the presence of these PfEMP1s on the infected erythrocyte surface is a risk factor for the development of CM.

There are several possible reasons why dual-binding PfEMP1s might contribute to the development of CM. One consequence of dual binding is increased endothelial adhesion under conditions of high shear stress (Figure 6). CM is associated with increased sequestered parasite biomass throughout the circulatory system, and this appears to have disproportionate effects on the regulation of the homeostasis of the brain, making increased adhesion a risk factor (Cunnington et al., 2013; Moxon et al., 2009; Storm and Craig, 2014). In addition to these general effects, specific consequences for the brain may occur due to modulation of signaling mediated by ICAM-1 and EPCR, as EPCR-mediated signaling and ICAM-1 expression are linked by a complex interplay that might also contribute to cerebral disease. Infected erythrocytes prevent EPCR from interacting with its natural ligands, inhibiting signaling pathways that induce anti-inflammatory responses and protect the integrity of the endothelium (Petersen et al., 2015; Turner et al., 2013). Indeed, inflammation and brain swelling both occur in CM and are important in disease development. In addition, blockage of EPCR signaling increases expression of several PfEMP1 adhesion receptors, including ICAM-1 (Moxon et al., 2013). Paradoxically, parasite sequestration in the brain is also linked to loss of EPCR, removing an important regulator of pro-coagulation responses (Moxon et al., 2013). The capacity to bind to both receptors may give the parasite more potential to resist endothelial changes in response to binding (i.e., downregulation of EPCR) and to remain sequestered in the brain. Together with the increased expression of ICAM-1 on endothelial cells, this could lead to a vicious cycle that further exacerbates disease.

CM is a complex disease, and in addition to dual-binding PfEMP1, various other aspects of both host and parasite biology will influence the development of cerebral symptoms. Other surface protein families, including STEVORs, RIFINs, and the PfEMP1 involved in rosetting (the clustering of infected and uninfected erythrocytes), have been implicated in severe malaria (Doumbo et al., 2009; Goel et al., 2015; Niang et al., 2014; Rowe et al., 1997) and further study is required to assess whether they too have a significant impact on the development of cerebral symptoms. In addition, host genetic factors, such as erythrocyte surface protein polymorphisms, could affect the capacity of the parasite to invade erythrocytes, altering parasitemia and therefore severity (Band et al., 2015). Nevertheless, the identification of dual-binding PfEMP1 as a risk factor for CM highlights these molecules as a promising and tractable candidate for the development of a vaccine to contribute to the prevention of this form of the disease.

A major challenge in targeting PfEMP1s through vaccination is their high sequence variability, driven by a diversifying selection

pressure to evade immune detection. This is demonstrated by structural studies of CIDRα domains bound to EPCR or CD36 that revealed their interaction sites to retain their overall chemical nature, while significantly diversifying in sequence (Hsieh et al., 2016; Lau et al., 2015). This reduces the likelihood of the natural development of cross-inhibitory antibody responses that target these critical surfaces of CIDR domains. In contrast, the ICAM-1-binding site of the group A PfEMP1 is remarkably conserved, with a number of absolutely or predominantly conserved amino acid residues that make critical, direct interactions with ICAM-1 (Figure 2G). Indeed, we find that plasma from *P. falciparum*-exposed individuals contains IgG with the capacity to inhibit ICAM-1 binding by a broad range of motif-containing DBLβ domains (Figure 4). This encourages future efforts to raise broadly reactive antibody responses against these domains. This study therefore highlights stronger endothelial adhesion capacity to both ICAM-1 and EPCR as a risk factor for development of CM and identifies a group of PfEMP1s with a remarkably conserved ICAM-1-binding site as potential vaccine immunogen for use as part of a strategy to prevent death due to CM.

EXPERIMENTAL PROCEDURES

More detailed methods and references are provided in the Supplemental Experimental Procedures.

Crystal Structure Determination

The PF11_0521 DBLβ3_D4 domain was produced as described in the Supplemental Experimental Procedures and was mixed with a 1.5 molar excess of ICAM-1^{D1D2} before size exclusion chromatography. Crystals were grown using sitting-drop vapor diffusion with a well solution of 10% (w/v) PEG 20000, 20% (v/v) PEG 500, and 0.1 M Tris-BICINE (pH 8.5) and cryo-cooled in well solution with an increased PEG 500 concentration of 32%.

Data were collected on beamline I02 (Diamond Light Source), indexed and refined using iMosflm, and scaled using SCALA (Evans and Murshudov, 2013). Molecular replacement was performed using Phaser-MR (McCoy et al., 2007) with a trimmed DBLα domain (PDB: 2XU0) and individual D1 and D2 domains from ICAM-1^{D1D2} (PDB: 1IC1) as search models. This identified one copy of the complex in the asymmetric unit. The structure was built and refined through iterative cycles of refinement using Refmac5 (Murshudov et al., 2011), Buster (Bricogne et al., 2016), and model building in Coot (Emsley et al., 2010).

Surface Plasmon Resonance

Surface plasmon resonance experiments were carried out in a Biacore T200 instrument (GE Healthcare). ICAM-1^{D1D5}-Fc was bound to a CM5 chip (GE Healthcare) pre-coupled to protein A while EPCR was biotinylated by BirA and coupled to a biotin CAPture chip (GE Healthcare). Binding partners were injected for 240 s with a dissociation time of 660 s. To analyze dual binding to ICAM-1 and EPCR, ICAM-1^{D1D5}-Fc was immobilized. Two- and three-domain PfEMP1 constructs were then injected, followed by a concentration series of EPCR. Kinetic sensorgrams were fitted to a 1:1 interaction model to determine kinetic rate constants and dissociation constant.

Small-Angle X-Ray Scattering

Small-angle X-ray scattering data were collected on beamline BM29 at the European Synchrotron Radiation Facility and processed using the ATSAS processing suite. The resulting ab initio model was converted into an envelope before manual model docking.

Patient Samples

Plasmodium falciparum isolates were collected from malaria patients at hospitals in Ghana, Tanzania, and Benin. Clinical manifestations of malaria were classified according to the definitions and associated criteria of the World Health Organization. CM was identified by a positive blood smear of the asexual form of *P. falciparum* and unrousable coma (Blantyre coma score, BCS $\leq$ 2) with exclusion of other causes of coma and severe illness. SA was identified by hemoglobin < 5g/dL and BCS > 2. OM was indicated by BCS > 2 and hemoglobin > 5 g/dL. These studies were approved by the respective ethics committees. Additional plasma samples were collected from *P. falciparum*-exposed children during a cross-sectional village study in Tanzania and from *falciparum*-exposed adult Liberians (Lusingu et al., 2004; Theisen et al., 2000).

Antibody Production and Purification

Rat antibodies were obtained by immunization with DBLβ domains, followed by affinity purification of IgG. Human antibodies against DBLβ domains or the ICAM-1-binding motif were affinity purified from pooled plasma from nine Liberian adults.

ELISA

ICAM-1 binding was assessed by ELISA using DBLβ domain-coated Maxisorp plates, with detection using ICAM-1^{D1D5}-Fc and rabbit anti-human IgG-HRP (Dako). A similar assay was used to assess the reactivity of human plasma samples to the DBLβ domains or to assess their efficacy at blocking ICAM-1 binding.

Assessment of Infected Erythrocyte Adhesion to Receptors

Plasmodium falciparum parasites were maintained in in vitro blood culture and selected for specific PfEMP1 expression using antibodies. Microslides (Ibidi) were coated with recombinant receptors (ICAM-1^{D1D5}-Fc, EPCR, or CD36) or seeded with HBMEC (Sciencell). Infected erythrocytes were exposed to the microslides at a range of physiological shear stresses and bound infected erythrocytes were quantified by microscopy.

Immunofluorescence

Infected erythrocytes were incubated with complexes of ICAM-1^{D1D5}-Fc:anti-human IgG-Dylight 488 (Abcam) and/or EPCR:anti-his IgG (QIAGEN):anti-mouse IgG Alexa 568 (Abcam) before washing and analysis in a Zeiss AxioImager Z1.

Real-Time qPCR

Parasite RNA from clinical samples was reverse transcribed into DNA and subjected to real-time qPCR using primer pairs (Table S7C) designed to amplify ICAM-1 motif-containing *var* genes or *var* gene subclasses encoding EPCR-binding CIDRα1 domains.

Sequence Analysis

DBLβ domain sequences (Rask et al., 2010) were used to blast extract DBLβ sequences from assemblies of Illumina whole-genome sequencing data, including MalariaGEN data (Miotto et al., 2015) and assembled amplicons from eight Ghanaian isolates. Those predicted to bind ICAM-1 were identified using search term ASNGGPGYYNTEVQK. A database containing sequences of the six most expressed *var* genes in 45 Tanzanian children with severe or uncomplicated malaria (Jespersen et al., 2016) was used to correlate relative expression of motif-containing *var* genes when compared with disease outcome. *Var* gene expression and disease severity were analyzed using the Wilcoxon-Mann-Whitney rank-sum test. To determine whether study site affected the outcome, a logistical regression model was used.

ACCESSION NUMBERS

Sequences originating from the present study are deposited in GenBank with accession numbers GenBank: KF984156, KJ866957, KJ866958, KJ866959, KM364031, KM364032, KM364033, and KM364034. Structure files have been deposited in the PDB under ID code PDB: 5MZA.

SUPPLEMENTAL INFORMATION

Supplemental Information includes Supplemental Experimental Procedures, seven figures, and seven tables and can be found with this article online at http://dx.doi.org/10.1016/j.chom.2017.02.009.

AUTHOR CONTRIBUTIONS

F.L., Y.A., A.B., T.L., M.K.H., and A.T.R.J. conceived and planned the study. F.L., Y.A., L.H., M.K.H., and A.T.R.J. wrote the manuscript. F.L., A.B., R.W.O., L.T., B.G., S.N.-S., and A.T.R.J. contributed with recombinant proteins and antibodies. F.L. purified and crystallized proteins and collected and analyzed small-angle X-ray scattering data. F.L. and M.K.H. prepared crystals, collected data, and solved the structure. F.L. and C.K.Y.L. performed and analyzed surface plasmon resonance experiments. Y.A. performed adhesion assays and IFA. A.B. and J.E.V.P. did parasite culturing and flow cytometry analysis. R.W.O. and G. E.-M. performed ELISA studies. R.W.O. and A.T.R.J. did real-time qPCR work. J.S.J., T.L., and A.T.R.J. did the sequencing and bioinformatics analysis. N.T.N., G.E.-M., A.M., M.F.O., J.P.L., C.W.W., and T.G.T. organized clinical work and processed clinical samples. All authors read and commented on the manuscript. F.L., Y.A., A.B., M.K.H., and A.T.R.J. contributed equally to the work.

ACKNOWLEDGMENTS

M.K.H. is a Wellcome Trust Investigator. A.B., A.T.R.J., C.W.W., G.E.-M., J.E.V.P., J.S.J., L.H., M.F.O., R.W.O., T.L., and Y.A. were supported by Novo Nordisk Fonden (NNF13OC0006249), Hørslev-Fonden, Augustinus Fonden (12-0378 and 15-3000), Lundbeckfonden (R180-2014-3098 and R140-2013-13448), Aase and Ejnar Danielsens Fond, Oda og Hans Svenningsens Fond (BOF-10109), Axel Muusfeldts Fond, Grosserer L.F. Foghts Fond, University of Copenhagen UCPH2016 Programme of Excellence, the Consultative Committee for Development Research (DANIDA), the Danish Council for Independent Research (4004-00032, T1333-00220, 1331-00089B, and Sapere Aude program DFF–4004-00624B), and the NIH (R01HL130678). Fieldwork in Benin was supported by PEERS-EPIVAC grant from AIRD/IRD. We thank the Beninese, Ghanaian, and Tanzanian donors; the MalariaGen community project for the use of data (Miotto et al., 2015); Michael Theisen for immune plasma from Liberia; and Mette Ulla Madsen and Maiken Høwning Visti for excellent technical assistance. We acknowledge the Core Facility for Integrated Microscopy, Faculty of Health and Medical Sciences, University of Copenhagen for use of the Zeiss AxioImage Z1 and Zachary Barbati for doing initial experiments not included in this paper. We thank David Staunton for assistance with biophysical experiments and Ed Lowe and the beamline scientists at I02 at Diamond Light Source for assistance with data collection. The accession codes for the sequences originating from Jespersen et al. (2016) are GenBank: KX154876, KX154960, KX154911, KX154945, KX154859, KX154816, KX154936, KX154871, KX154901, KX154818, KX154872, KX154935, and KX154836.

Received: October 4, 2016
Revised: January 20, 2017
Accepted: February 10, 2017
Published: March 8, 2017

REFERENCES

Avril, M., Tripathi, A.K., Brazier, A.J., Andisi, C., Janes, J.H., Soma, V.L., Sullivan, D.J., Jr., Bull, P.C., Stins, M.F., and Smith, J.D. (2012). A restricted subset of var genes mediates adherence of Plasmodium falciparum-infected erythrocytes to brain endothelial cells. Proc. Natl. Acad. Sci. USA *109*, E1782–E1790.

Avril, M., Bernabeu, M., Benjamin, M., Brazier, A.J., and Smith, J.D. (2016). Interaction between endothelial protein C receptor and intercellular adhesion molecule 1 to mediate binding of Plasmodium falciparum-infected erythrocytes to endothelial cells. MBio *7*, e00615-16.

Band, G., Rockett, K.A., Spencer, C.C., and Kwiatkowski, D.P.; Malaria Genomic Epidemiology Network (2015). A novel locus of resistance to severe malaria in a region of ancient balancing selection. Nature *526*, 253–257.

Bengtsson, A., Joergensen, L., Rask, T.S., Olsen, R.W., Andersen, M.A., Turner, L., Theander, T.G., Hviid, L., Higgins, M.K., Craig, A., et al. (2013). A novel domain cassette identifies Plasmodium falciparum PfEMP1 proteins binding ICAM-1 and is a target of cross-reactive, adhesion-inhibitory antibodies. J. Immunol. *190*, 240–249.

Bernabeu, M., Danziger, S.A., Avril, M., Vaz, M., Babar, P.H., Brazier, A.J., Herricks, T., Maki, J.N., Pereira, L., Mascarenhas, A., et al. (2016). Severe adult malaria is associated with specific PfEMP1 adhesion types and high parasite biomass. Proc. Natl. Acad. Sci. USA *113*, E3270–E3279.

Birbeck, G.L., Molyneux, M.E., Kaplan, P.W., Seydel, K.B., Chimalizeni, Y.F., Kawaza, K., and Taylor, T.E. (2010). Blantyre Malaria Project Epilepsy Study (BMPES) of neurological outcomes in retinopathy-positive paediatric cerebral malaria survivors: a prospective cohort study. Lancet Neurol. *9*, 1173–1181.

Bricogne, G., Blanc, E., Brandl, M., Flensburg, C., Keller, P., Paciorek, W., Roversi, P., Sharff, A., Smart, O.S., Vonrhein, C., and Womack, T.O. (2016). BUSTER version 2.10.2 (Global Phasing).

Claessens, A., Adams, Y., Ghumra, A., Lindergard, G., Buchan, C.C., Andisi, C., Bull, P.C., Mok, S., Gupta, A.P., Wang, C.W., et al. (2012). A subset of group A-like var genes encodes the malaria parasite ligands for binding to human brain endothelial cells. Proc. Natl. Acad. Sci. USA *109*, E1772–E1781.

Cunnington, A.J., Riley, E.M., and Walther, M. (2013). Stuck in a rut? Reconsidering the role of parasite sequestration in severe malaria syndromes. Trends Parasitol. *29*, 585–592.

Dondorp, A.M., Fanello, C.I., Hendriksen, I.C., Gomes, E., Seni, A., Chhaganlal, K.D., Bojang, K., Olaosebikan, R., Anunobi, N., Maitland, K., et al.; AQUAMAT group (2010). Artesunate versus quinine in the treatment of severe falciparum malaria in African children (AQUAMAT): an open-label, randomised trial. Lancet *376*, 1647–1657.

Doumbo, O.K., Thera, M.A., Koné, A.K., Raza, A., Tempest, L.J., Lyke, K.E., Plowe, C.V., and Rowe, J.A. (2009). High levels of Plasmodium falciparum rosetting in all clinical forms of severe malaria in African children. Am. J. Trop. Med. Hyg. *81*, 987–993.

Emsley, P., Lohkamp, B., Scott, W.G., and Cowtan, K. (2010). Features and development of Coot. Acta Crystallogr. D Biol. Crystallogr. *66*, 486–501.

Evans, P.R., and Murshudov, G.N. (2013). How good are my data and what is the resolution? Acta Crystallogr. D Biol. Crystallogr. *69*, 1204–1214.

Goel, S., Palmkvist, M., Moll, K., Joannin, N., Lara, P., Akhouri, R.R., Moradi, N., Öjemalm, K., Westman, M., Angeletti, D., et al. (2015). RIFINs are adhesins implicated in severe Plasmodium falciparum malaria. Nat. Med. *21*, 314–317.

Howell, D.P., Levin, E.A., Springer, A.L., Kraemer, S.M., Phippard, D.J., Schief, W.R., and Smith, J.D. (2008). Mapping a common interaction site used by *Plasmodium falciparum* Duffy binding-like domains to bind diverse host receptors. Mol. Microbiol. *67*, 78–87.

Hsieh, F.L., Turner, L., Bolla, J.R., Robinson, C.V., Lavstsen, T., and Higgins, M.K. (2016). The structural basis for CD36 binding by the malaria parasite. Nat. Commun. *7*, 12837.

Hviid, L., and Jensen, A.T. (2015). PfEMP1—a parasite protein family of key importance in *Plasmodium falciparum* malaria immunity and pathogenesis. Adv. Parasitol. *88*, 51–84.

Idro, R., Jenkins, N.E., and Newton, C.R. (2005). Pathogenesis, clinical features, and neurological outcome of cerebral malaria. Lancet Neurol. *4*, 827–840.

Janes, J.H., Wang, C.P., Levin-Edens, E., Vigan-Womas, I., Guillotte, M., Melcher, M., Mercereau-Puijalon, O., and Smith, J.D. (2011). Investigating the host binding signature on the Plasmodium falciparum PfEMP1 protein family. PLoS Pathog. *7*, e1002032.

Jensen, A.T., Magistrado, P., Sharp, S., Joergensen, L., Lavstsen, T., Chiucchiuini, A., Salanti, A., Vestergaard, L.S., Lusingu, J.P., Hermsen, R., et al. (2004). *Plasmodium falciparum* associated with severe childhood malaria preferentially expresses PfEMP1 encoded by group A *var* genes. J. Exp. Med. *199*, 1179–1190.

Jespersen, J.S., Wang, C.W., Mkumbaye, S.I., Minja, D.T., Petersen, B., Turner, L., Petersen, J.E., Lusingu, J.P., Theander, T.G., and Lavstsen, T. (2016). Plasmodium falciparum var genes expressed in children with severe malaria encode CIDRα1 domains. EMBO Mol. Med. *8*, 839–850.

Kim, J.H., Singvall, J., Schwarz-Linek, U., Johnson, B.J., Potts, J.R., and Höök, M. (2004). BBK32, a fibronectin binding MSCRAMM from Borrelia burgdorferi, contains a disordered region that undergoes a conformational change on ligand binding. J. Biol. Chem. *279*, 41706–41714.

Kwong, P.D., Wyatt, R., Robinson, J., Sweet, R.W., Sodroski, J., and Hendrickson, W.A. (1998). Structure of an HIV gp120 envelope glycoprotein in complex with the CD4 receptor and a neutralizing human antibody. Nature *393*, 648–659.

Lau, C.K., Turner, L., Jespersen, J.S., Lowe, E.D., Petersen, B., Wang, C.W., Petersen, J.E., Lusingu, J., Theander, T.G., Lavstsen, T., and Higgins, M.K. (2015). Structural conservation despite huge sequence diversity allows EPCR binding by the PfEMP1 family implicated in severe childhood malaria. Cell Host Microbe *17*, 118–129.

Lavstsen, T., Turner, L., Saguti, F., Magistrado, P., Rask, T.S., Jespersen, J.S., Wang, C.W., Berger, S.S., Baraka, V., Marquard, A.M., et al. (2012). Plasmodium falciparum erythrocyte membrane protein 1 domain cassettes 8 and 13 are associated with severe malaria in children. Proc. Natl. Acad. Sci. USA *109*, E1791–E1800.

Lusingu, J.P., Vestergaard, L.S., Mmbando, B.P., Drakeley, C.J., Jones, C., Akida, J., Savaeli, Z.X., Kitua, A.Y., Lemnge, M.M., and Theander, T.G. (2004). Malaria morbidity and immunity among residents of villages with different Plasmodium falciparum transmission intensity in North-Eastern Tanzania. Malar. J. *3*, 26.

McCoy, A.J., Grosse-Kunstleve, R.W., Adams, P.D., Winn, M.D., Storoni, L.C., and Read, R.J. (2007). Phaser crystallographic software. J. Appl. Cryst. *40*, 658–674.

Menschikowski, M., Hagelgans, A., Eisenhofer, G., and Siegert, G. (2009). Regulation of endothelial protein C receptor shedding by cytokines is mediated through differential activation of MAP kinase signaling pathways. Exp. Cell Res. *315*, 2673–2682.

Miotto, O., Amato, R., Ashley, E.A., MacInnis, B., Almagro-Garcia, J., Amaratunga, C., Lim, P., Mead, D., Oyola, S.O., Dhorda, M., et al. (2015). Genetic architecture of artemisinin-resistant Plasmodium falciparum. Nat. Genet. *47*, 226–234.

Mkumbaye, S.I., Wang, C.W., Lyimo, E., Jespersen, J.S., Manjurano, A., Mosha, J., Kavishe, R.A., Mwakalinga, S.B., Minja, D.T., Lusingu, J.P., et al. (2017). The severity of Plasmodium falciparum infection is associated with transcript levels of var genes encoding EPCR-binding PfEMP1. Infect. Immun. Published online January 30, 2017. http://dx.doi.org/10.1128/IAI.00841-16.

Moxon, C.A., Heyderman, R.S., and Wassmer, S.C. (2009). Dysregulation of coagulation in cerebral malaria. Mol. Biochem. Parasitol. *166*, 99–108.

Moxon, C.A., Wassmer, S.C., Milner, D.A., Jr., Chisala, N.V., Taylor, T.E., Seydel, K.B., Molyneux, M.E., Faragher, B., Esmon, C.T., Downey, C., et al. (2013). Loss of endothelial protein C receptors links coagulation and inflammation to parasite sequestration in cerebral malaria in African children. Blood *122*, 842–851.

Murshudov, G.N., Skubák, P., Lebedev, A.A., Pannu, N.S., Steiner, R.A., Nicholls, R.A., Winn, M.D., Long, F., and Vagin, A.A. (2011). REFMAC5 for the refinement of macromolecular crystal structures. Acta Crystallogr. D Biol. Crystallogr. *67*, 355–367.

Newbold, C., Warn, P., Black, G., Berendt, A., Craig, A., Snow, B., Msobo, M., Peshu, N., and Marsh, K. (1997). Receptor-specific adhesion and clinical disease in Plasmodium falciparum. Am. J. Trop. Med. Hyg. *57*, 389–398.

Niang, M., Bei, A.K., Madnani, K.G., Pelly, S., Dankwa, S., Kanjee, U., Gunalan, K., Amaladoss, A., Yeo, K.P., Bob, N.S., et al. (2014). STEVOR is a Plasmodium falciparum erythrocyte binding protein that mediates merozoite invasion and rosetting. Cell Host Microbe *16*, 81–93.

Ochola, L.B., Siddondo, B.R., Ocholla, H., Nkya, S., Kimani, E.N., Williams, T.N., Makale, J.O., Liljander, A., Urban, B.C., Bull, P.C., et al. (2011). Specific receptor usage in Plasmodium falciparum cytoadherence is associated with disease outcome. PLoS ONE *6*, e14741.

Oleinikov, A.V., Amos, E., Frye, I.T., Rossnagle, E., Mutabingwa, T.K., Fried, M., and Duffy, P.E. (2009). High throughput functional assays of the variant antigen PfEMP1 reveal a single domain in the 3D7 *Plasmodium falciparum* genome that binds ICAM1 with high affinity and is targeted by naturally acquired neutralizing antibodies. PLoS Pathog. *5*, e1000386.

Petersen, J.E., Bouwens, E.A., Tamayo, I., Turner, L., Wang, C.W., Stins, M., Theander, T.G., Hermida, J., Mosnier, L.O., and Lavstsen, T. (2015). Protein C system defects inflicted by the malaria parasite protein PfEMP1 can be overcome by a soluble EPCR variant. Thromb. Haemost. *114*, 1038–1048.

Rask, T.S., Hansen, D.A., Theander, T.G., Gorm Pedersen, A., and Lavstsen, T. (2010). Plasmodium falciparum erythrocyte membrane protein 1 diversity in seven genomes—divide and conquer. PLoS Comput. Biol. *6*, e1000933.

Robinson, B.A., Welch, T.L., and Smith, J.D. (2003). Widespread functional specialization of *Plasmodium falciparum* erythrocyte membrane protein 1 family members to bind CD36 analysed across a parasite genome. Mol. Microbiol. *47*, 1265–1278.

Rogerson, S.J., Tembenu, R., Dobaño, C., Plitt, S., Taylor, T.E., and Molyneux, M.E. (1999). Cytoadherence characteristics of Plasmodium falciparum-infected erythrocytes from Malawian children with severe and uncomplicated malaria. Am. J. Trop. Med. Hyg. *61*, 467–472.

Rowe, J.A., Moulds, J.M., Newbold, C.I., and Miller, L.H. (1997). P. falciparum rosetting mediated by a parasite-variant erythrocyte membrane protein and complement-receptor 1. Nature *388*, 292–295.

Seydel, K.B., Kampondeni, S.D., Valim, C., Potchen, M.J., Milner, D.A., Muwalo, F.W., Birbeck, G.L., Bradley, W.G., Fox, L.L., Glover, S.J., et al. (2015). Brain swelling and death in children with cerebral malaria. N. Engl. J. Med. *372*, 1126–1137.

Silamut, K., Phu, N.H., Whitty, C., Turner, G.D., Louwrier, K., Mai, N.T., Simpson, J.A., Hien, T.T., and White, N.J. (1999). A quantitative analysis of the microvascular sequestration of malaria parasites in the human brain. Am. J. Pathol. *155*, 395–410.

Storm, J., and Craig, A.G. (2014). Pathogenesis of cerebral malaria—inflammation and cytoadherence. Front. Cell. Infect. Microbiol. *4*, 100.

Theisen, M., Soe, S., Jessing, S.G., Okkels, L.M., Danielsen, S., Oeuvray, C., Druilhe, P., and Jepsen, S. (2000). Identification of a major B-cell epitope of the Plasmodium falciparum glutamate-rich protein (GLURP), targeted by human antibodies mediating parasite killing. Vaccine *19*, 204–212.

Turner, G.D., Morrison, H., Jones, M., Davis, T.M., Looareesuwan, S., Buley, I.D., Gatter, K.C., Newbold, C.I., Pukritayakamee, S., Nagachinta, B., et al. (1994). An immunohistochemical study of the pathology of fatal malaria. Evidence for widespread endothelial activation and a potential role for intercellular adhesion molecule-1 in cerebral sequestration. Am. J. Pathol. *145*, 1057–1069.

Turner, L., Lavstsen, T., Berger, S.S., Wang, C.W., Petersen, J.E., Avril, M., Brazier, A.J., Freeth, J., Jespersen, J.S., Nielsen, M.A., et al. (2013). Severe malaria is associated with parasite binding to endothelial protein C receptor. Nature *498*, 502–505.

Cell Host & Microbe, Volume *21*

Supplemental Information

Structure-Guided Identification of a Family of Dual Receptor-Binding PfEMP1 that Is Associated with Cerebral Malaria

Frank Lennartz, Yvonne Adams, Anja Bengtsson, Rebecca W. Olsen, Louise Turner, Nicaise T. Ndam, Gertrude Ecklu-Mensah, Azizath Moussiliou, Michael F. Ofori, Benoit Gamain, John P. Lusingu, Jens E.V. Petersen, Christian W. Wang, Sofia Nunes-Silva, Jakob S. Jespersen, Clinton K.Y. Lau, Thor G. Theander, Thomas Lavstsen, Lars Hviid, Matthew K. Higgins, and Anja T.R. Jensen

Supplemental Figures

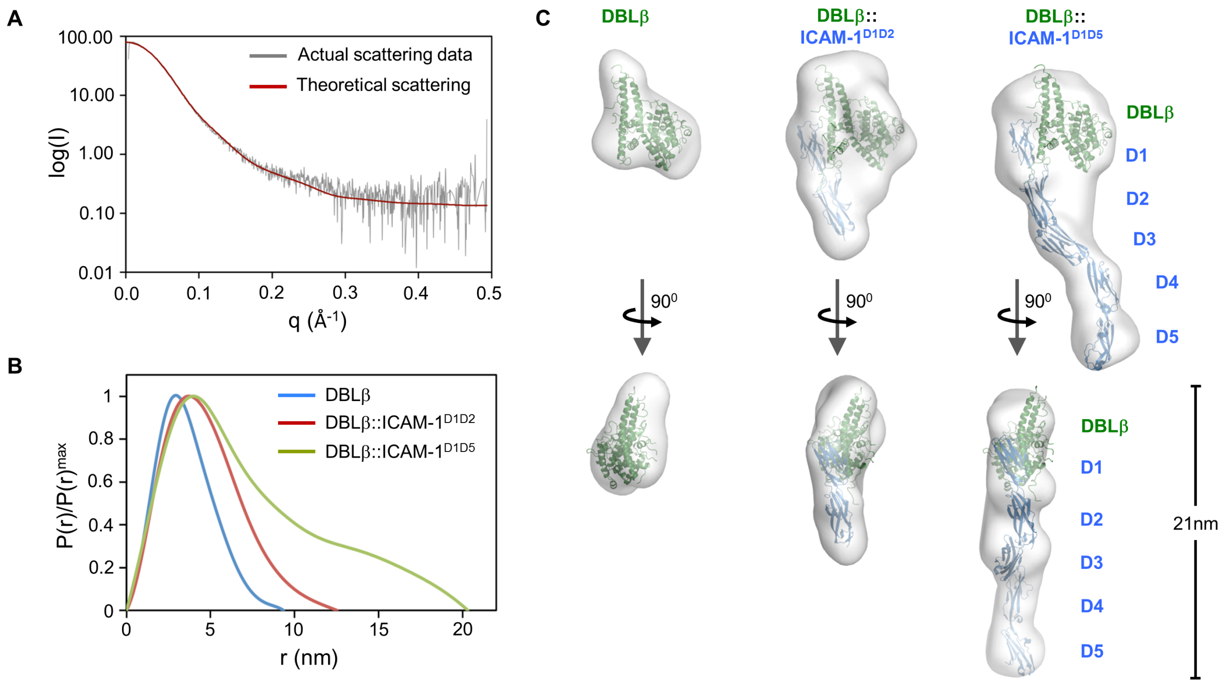

D

	$Rg_{exp.}$ (nm)	D_{max} (nm)	V_{porod} (nm^3)	Mr_{exp} (kDa)	Mr_{app} (kDa)	χ
DBLβ	2.72	9.35	88	55	51	1.006
DBLβ::ICAM-1^{D1D2}	3.58	12.53	140	77	82	1.185
DBLβ::ICAM-1^{D1D5}	5.80	20.31	237	131	139	1.209

Figure S1. SAXS analysis of the PF11_0521 DBLβ3_D4:ICAM-1 complex, ***related to Figure 1***

(A) Comparison between solution scattering data collected for the PF11_0521 DBLβ3_D4:ICAM-1^{D1D2} complex and the theoretical scattering calculated from the crystal structure of the same complex. The fit of experimental and theoretical scattering was calculated with Crysol, giving a χ^2 of 3.58.

(B) Normalized distance distribution function P(r) for PF11_0521 DBLβ3_D4 alone and bound to ICAM-1^{D1D2} or ICAM-1^{D1D5}.

(C) *Ab initio* envelopes calculated from SAXS data for the PF11_0521 DBLβ3_D4 domain alone and in complex with ICAM-1^{D1D2} or ICAM-1^{D1D5}. The structures of the PF11_0521 DBLβ3_D4 domain (from the co-crystal structure of PF11_0521 DBLβ3_D4:ICAM-1^{D1D2} complex), the PF11_0521 DBLβ3_D4: ICAM-1^{D1D2} complex and the structure of ICAM-1^{D3D5} (PDB 2OZ4) were manually docked into the envelopes.

(D) Parameters derived from solution scattering data. The radius of gyration R_g was determined using AutoRg, the maximum particle diameter D_{max} was calculated with GNOM. The Porod volume V_{porod} was calculated with PRIMUS. Mr_{exp} is the expected molecular mass, Mr_{app} is the mass calculated from the excluded volume in the final model derived from DAMMIN, divided by 2. χ is the best fit of the average from 20 low-resolution shape reconstructions to the experimental scattering data.

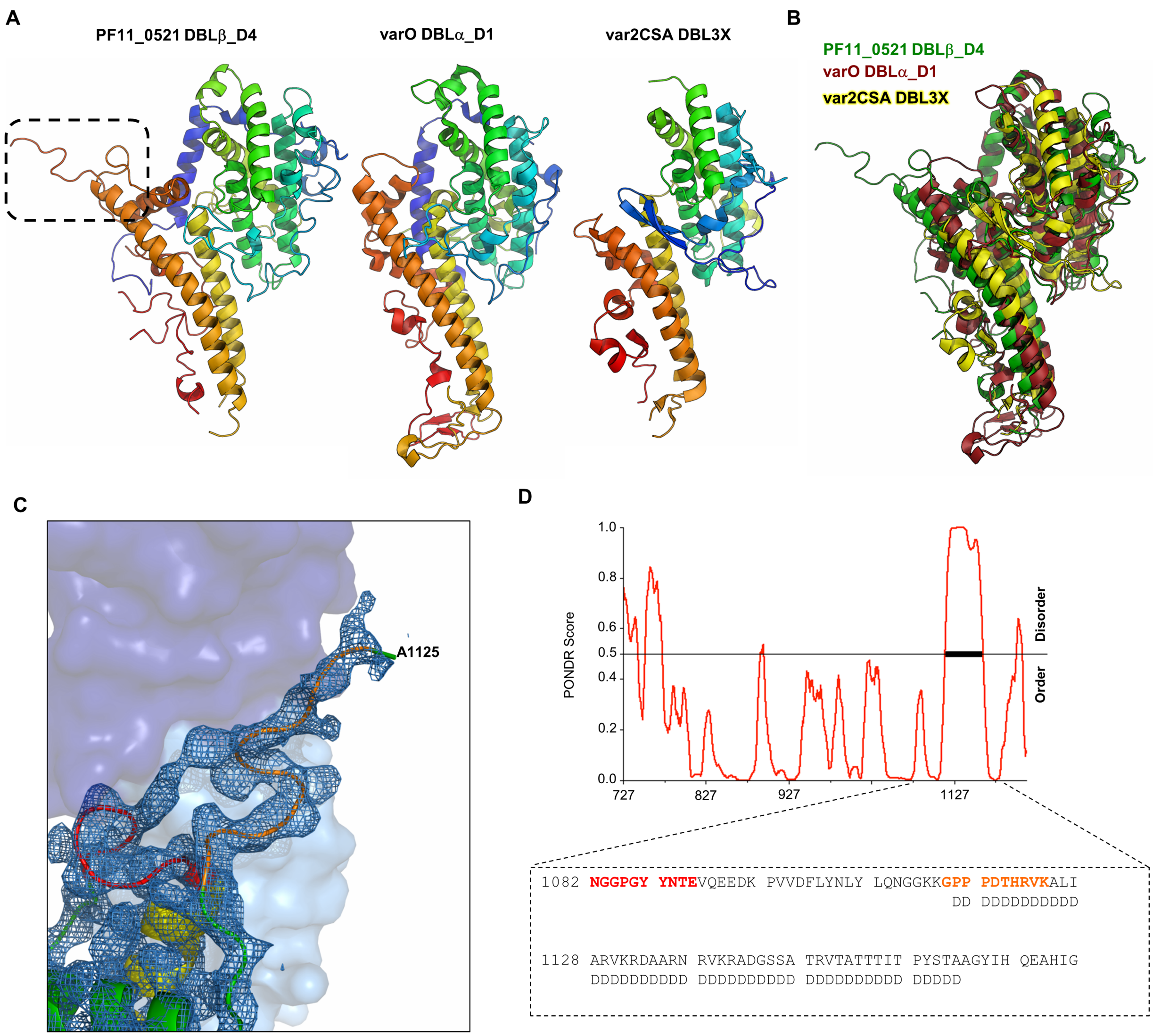

Figure S2. Unusual structural features of the ICAM-1 binding site in group A PfEMP1, *related to Figure 1*

(A) Comparison between known DBL domain structures. DBL domains from the PF11_0521, varO (PDB 2XU0) and vars2CSA (PDB 3BQI) PfEMP1 are shown in similar orientation for comparison and colored-coded from the N-terminus (blue) to C-terminus (red). The dashed box indicates the ICAM-1 binding site in the PF11_0521 DBLβ3_D4 domain.

(B) Superposition of the structures for the DBL domains of PF11_0521 (green), varO (red) and var2CSA (yellow). The superposition was prepared with PyMol (Schroedinger).

(C) Electron density map (2FoFc map) for residues in site 3 of the ICAM-1 binding site. The map is shown at a σ level of 1.0. Parts of the DBL domain and ICAM-1 are color coded according to Figure 1 of the main text. The last visible side chain of the loop that contains site 3 is indicated.

(D) Disordered regions in the PF11_0521 DBLβ3_D4 domain were predicted using PONDR (http://www.pondr.com/). A PONDR score of >0.5 indicates disorder. The inset shows the sequence of a potentially disordered region in the PF11_0521 DBLβ3_D4 domain. Residues in site 2 (red) and 3 (orange) of the ICAM-1 binding site are highlighted.

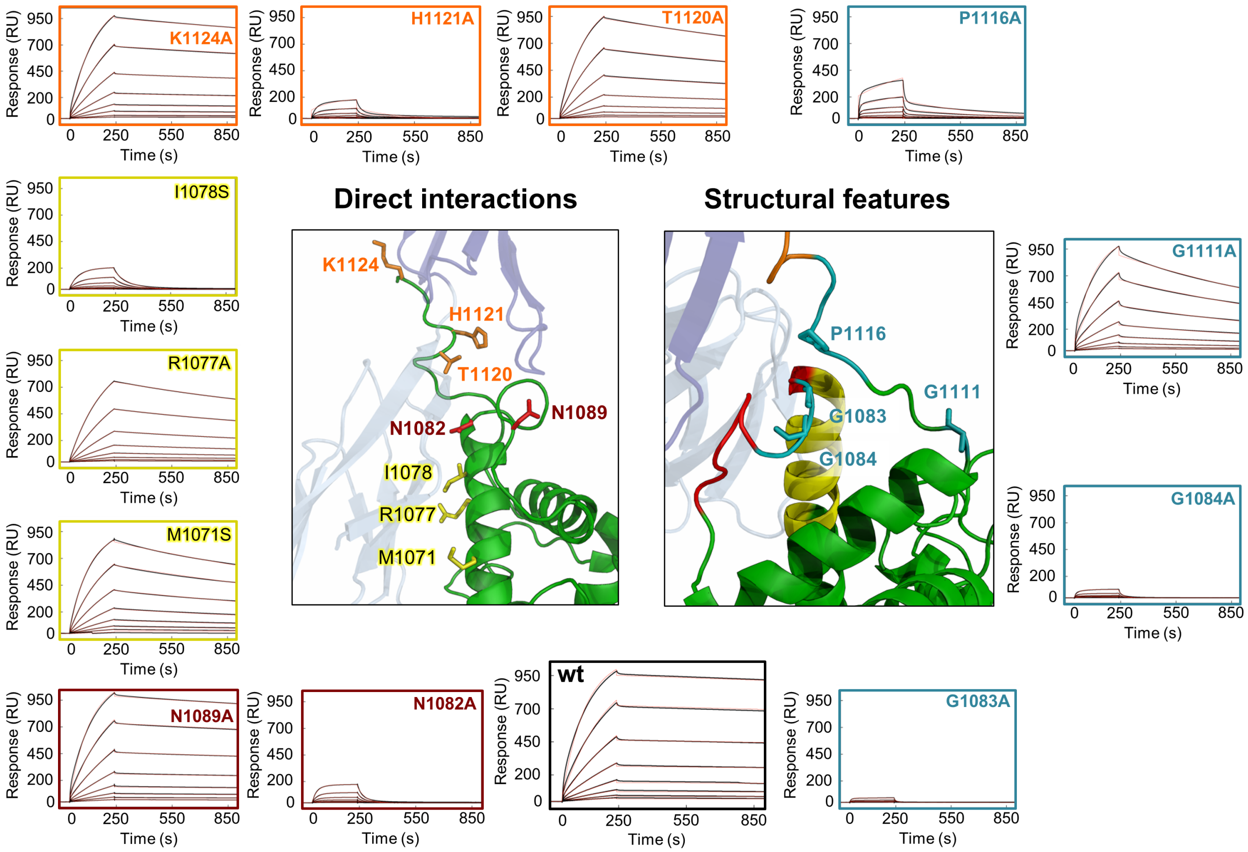

Figure S3. Detailed analysis of the ICAM-1-binding site of the PF11_0521 DBLβ3_D4 domain, ***related to Figure 2***

Surface plasmon resonance analysis of the effect of binding site mutations on the interaction between PF11_0521 DBLβ3_D4 and ICAM-1^{D1D5}. The sites within the PF11_0521 DBLβ3_D4-binding site and residues within these sites that directly contact ICAM-1 are indicated (site 1, yellow; site 2, red; site 3, orange). Structural residues (teal) important for positioning of interacting residues are highlighted. For SPR measurements, ICAM-1^{D1D5}-Fc was immobilized onto the chip and wild type or mutant PF11_0521 DBLβ3_D4 were injected over the chip in two-fold dilution series from 500 nM to 4 nM. Data (black lines) are modelled to a one-site model (red lines).

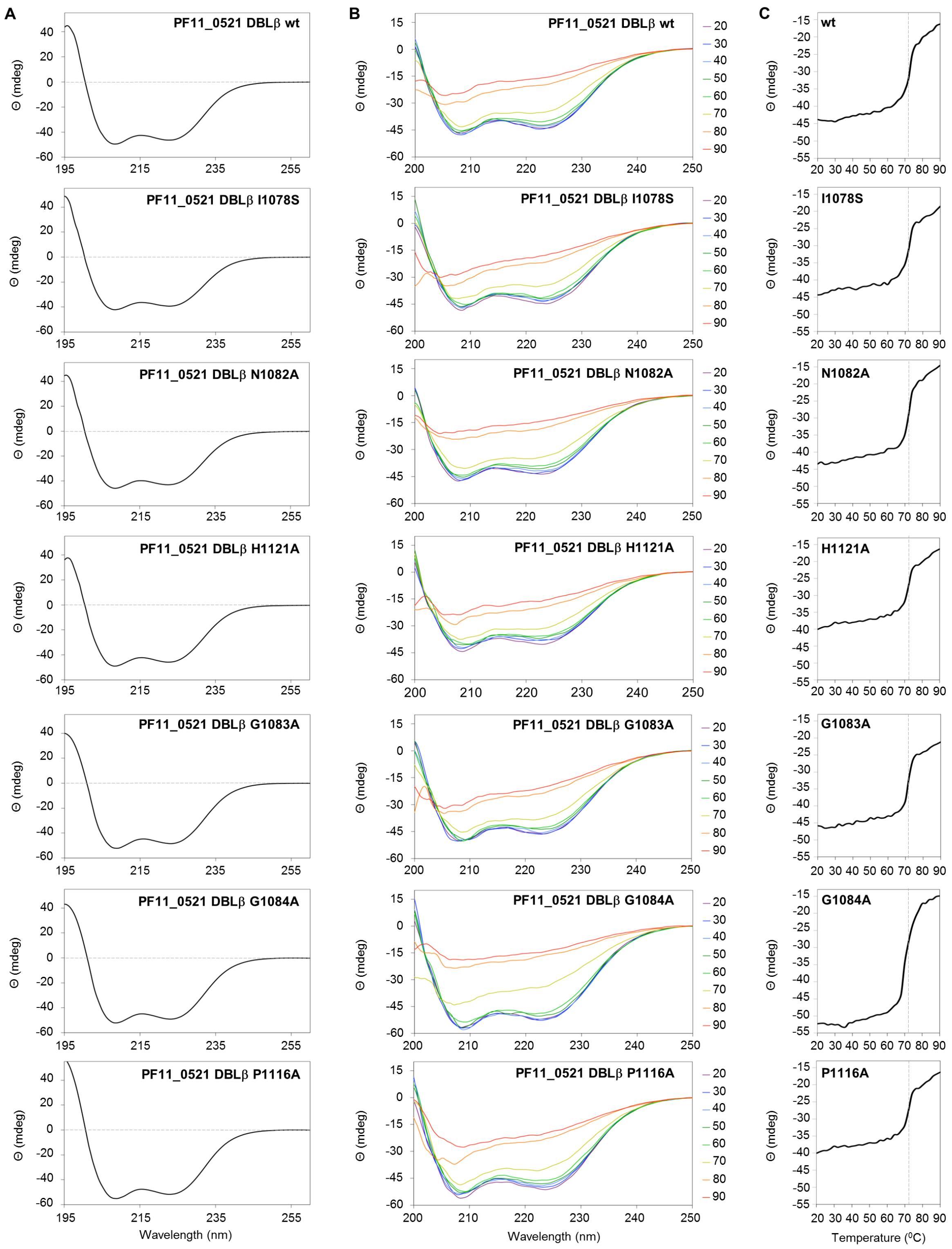

Figure S4. CD spectroscopy analysis of key PF11_0521 DBLβ3_D4 mutants, ***related to Figure 2***

(A) Secondary structure analysis of PF11_0521 DBLβ3_D4 wt, I1078S, N1082A, H1121A, G1083A, G1084A and P1116A by CD spectroscopy to confirm folding of the mutant proteins. CD spectra were recorded at 20°C between wavelengths of 195 nm and 260 nm. For each sample, four measurements were averaged and corrected for buffer absorption.

(B) Thermal denaturation analysis of PF11_0521 DBLβ3_D4 wt and mutants. CD spectra were recorded between 200 nm and 250 nm and after each measurement the temperature was raised by 0.5°C increments. Shown are spectra for 10°C intervals between 20°C and 90°C.

(C) Thermal melting curve for PF11_0521 DBLβ3_D4 wt and mutants. The ellipticity at a fixed wavelength of 222 nm was measured between 20°C and 90°C. After each measurement the temperature was increased by 0.5°C. Dashed lines indicate the respective melting temperature.

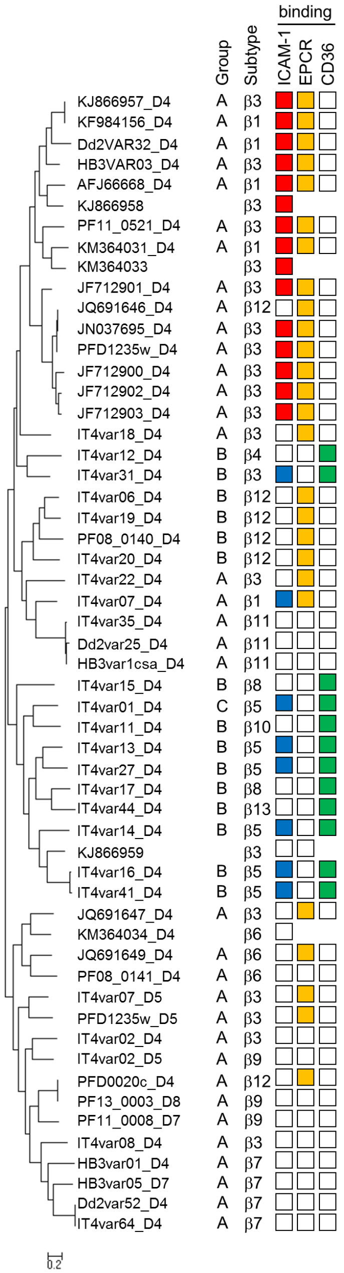

Figure S5. Phylogenetic analysis and binding phenotype of 55 DBLβ domains, ***related to Figures 2 and 3***

Maximum likelihood tree of 55 complete DBLβ domains generated as described in Figure 3. Red: ICAM-1 binding DBLβ with motif; Blue: ICAM-1 binding DBLβ with no motif; Orange: EPCR binding CIDRα1; Green: CD36 binding CIDRα; White: no binding; Blank space: information not available.

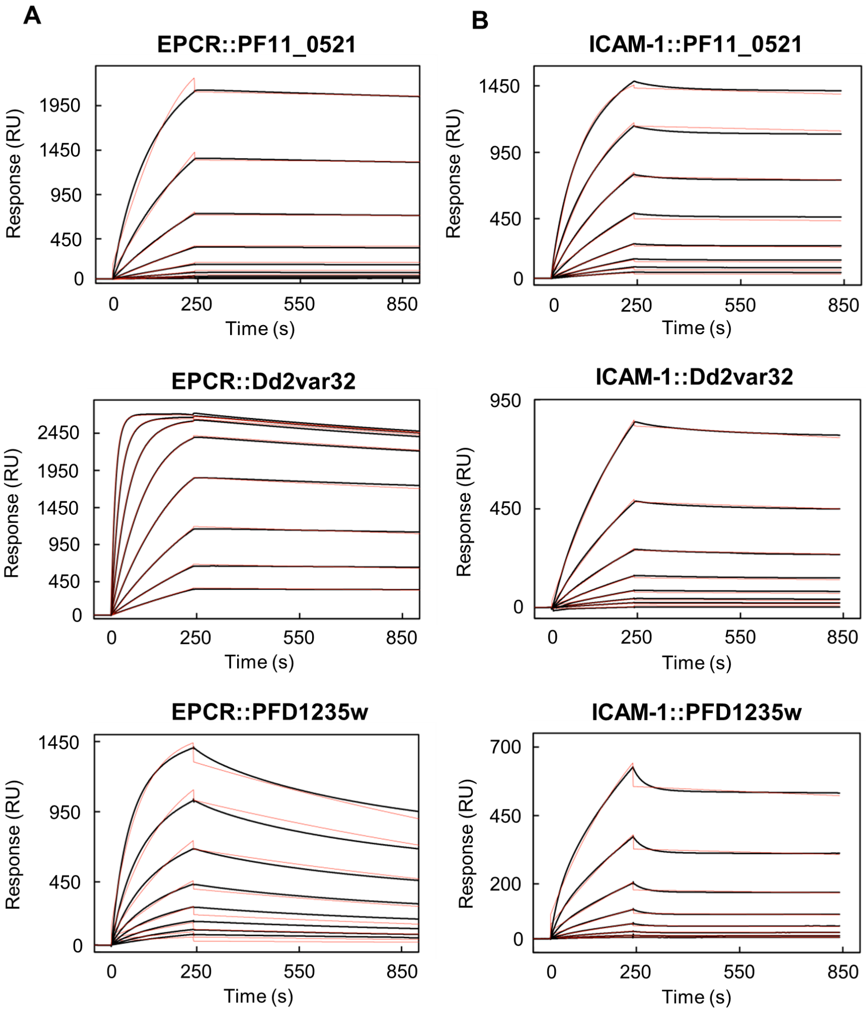

Figure S6. SPR analysis for binding of PfEMP1 head structures to ICAM-1 or EPCR, *related to Figure 5*

(A) PfEMP1 head structure binding to EPCR. EPCR was immobilized onto the chip, and PF11_0521, Dd2var32 or PFD1235w head structures were then injected over the chip surface in two-fold dilutions from 4 to 500 nM.

(B) PfEMP1 head structure binding to ICAM-1^{D1D5}. ICAM-1^{D1D5}-Fc was immobilized onto the chip and PF11_0521, Dd2var32 or PFD1235w head structures were injected over the chip surface in two-fold dilutions from 4 to 500 nM. Data (black lines) are modelled to a one-site model (red lines).

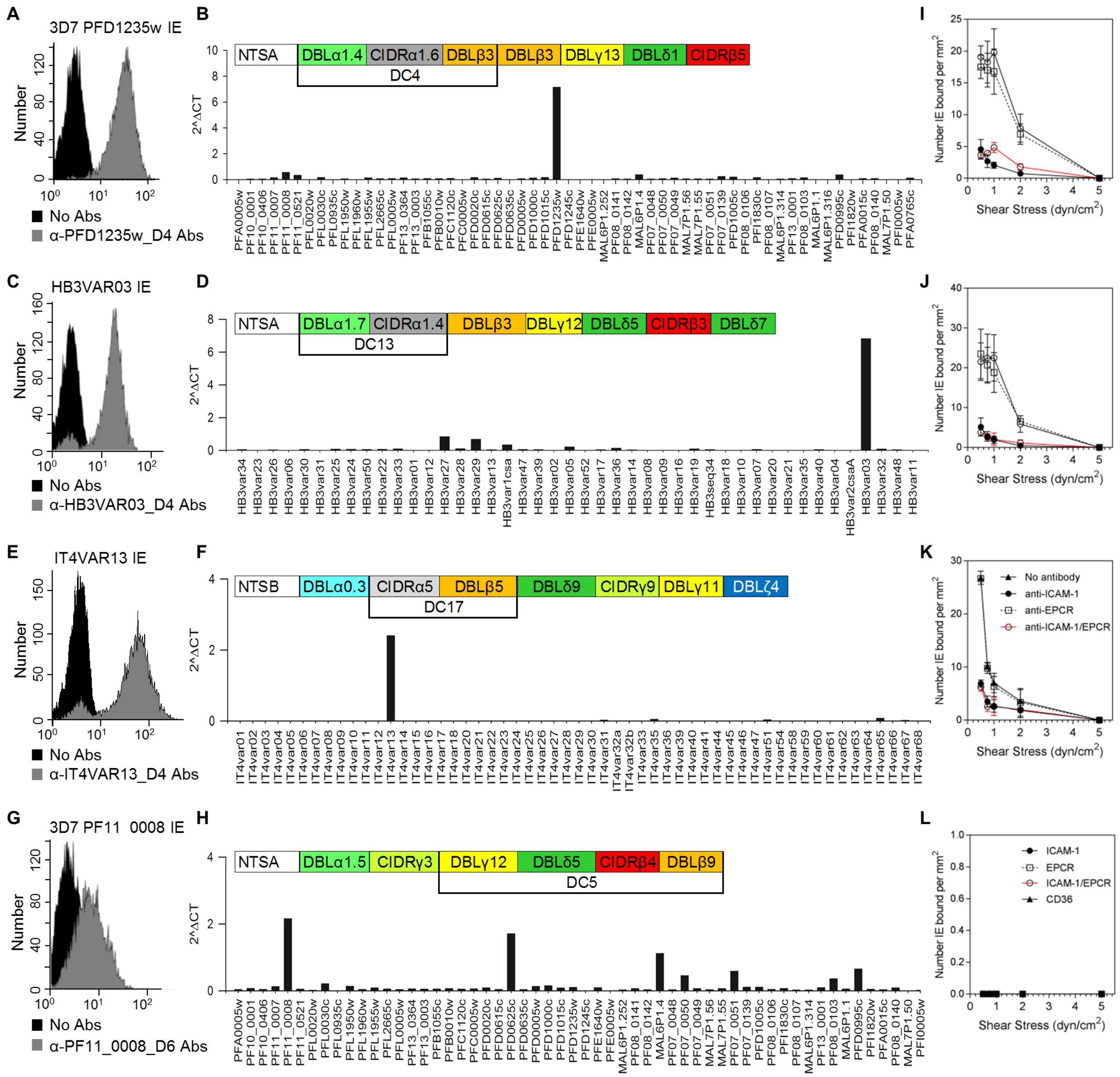

Figure S7. *var*/PfEMP1 expression profiles of HB3, IT4, and 3D7 parasite lines and adhesion to activated HBMEC and recombinant receptors, *related to Figure 6*

(A) Ethidium bromide-stained 3D7 PFD1235w IEs with (grey histogram) and without (black histogram) rat PFD1235w DBLβ3_D4 antiserum.

(B) Transcript level of each *var* gene of 3D7 PFD1235W IE relative to the control gene (*seryl-tRNA synthetase*). The domain architecture of the dominant expressed PfEMP1, PFD1235w is shown.

(C) Ethidium bromide stained HB3VAR03 IE with (grey histogram) and without (black histogram) rat VAR03_D4 antiserum.

(D) Transcript level of each *var* gene of HB3VAR03 IE relative to the control gene (*seryl-tRNA synthetase*). The domain architecture of the dominant expressed PfEMP1, VAR03 is shown.

(E) Ethidium bromide stained IT4VAR13 IE with (grey histogram) and without (black histogram) rat VAR13_D4 antiserum.

(F) Transcript level of each *var* gene of IT4VAR13 IE relative to the control gene (*seryl-tRNA synthetase*). The domain architecture of the dominant expressed PfEMP1, VAR13 is shown.

(G) Ethidium bromide stained 3D7PF11_0008 IE with (grey histogram) and without (black histogram) rat PF11_0008_D6 antiserum.

(H) Transcript level of each *var* gene of 3D7 PF11_0008 IE relative to the control gene (*seryl-tRNA synthetase*). The domain architecture of the dominant expressed PfEMP1, PF11_0008 is shown.

(I- K) Number of IE bound per mm^2 (± SEM) on HBMEC activated overnight with 10 ng/ml TNFα.

(L) Adhesion of 3D7PF11_0008 IE to recombinant CD36, EPCR and ICAM-1.

Supplemental Tables

Table S1. Data collection and refinement statistics, *related to Figure 1*

Values in parentheses are for highest-resolution shell.	PF11_0521 DBLβ3_D4-ICAM-1^{D1D2}
Data collection	
Space group	$P2_12_12_1$
Cell dimensions	
a, b, c (Å)	68.64, 109.83, 112.83
α, β, γ (°)	90.00, 90.00, 90.00
Resolution (Å)	78.70-2.78 (2.93-2.78)
R_{merge}	0.13 (0.42)
R_{pim}	0.07 (0.21)
I / σI	8.2 (3.3)
Completeness (%)	99.8 (100)
Redundancy	4.5 (4.7)
$CC_{1/2}$	0.99 (0.80)
No. unique reflections	22,210 (3,168)
Refinement	
Resolution (Å)	2.78
No. Reflections	22,036
R_{work} / R_{free}	20.48/23.56
No. Atoms	
Protein	4,970
Ligand/ion	111
Water	10
B-factors	
Protein	46.83
Ligand/ion	86.12
Water	29.67
R.m.s. deviations	
Bond lengths (Å)	0.007
Bond angles (°)	0.97

Table S2. List of contacts between PF11_0521 DBLβ3_D4 and ICAM-1^{D1D2}, *related to Figures 1 and 2*

Colors correspond to PF11_0521 DBLβ3_D4 residues as color-coded in Figures 1 and 2 of the main text.

PF11_0521 DBLβ3_D4		ICAM-1^{D1D2}		
**Residue**	**Group**	**Residue**	**Group**	**Type of Interaction**
Tyr1067	Sidechain	Pro45	Sidechain	Hydrophobic
Tyr1067	Sidechain	Leu44	Sidechain	Hydrophobic
**Met1071**	Sidechain	Val51	Sidechain	Hydrophobic
Ala1074	Sidechain	Leu42	Sidechain	Hydrophobic
**Arg1077**	Sidechain NH_2	Glu53	Sidechain O	Hydrogen bond
**Ile1078**	Sidechain	Leu18	Sidechain	Hydrophobic
**Ile1078**	Sidechain	Glu53	Sidechain	Hydrophobic
**Asn1082**	Sidechain O	Ser16	Backbone N	Hydrogen bond
**Asn1082**	Sidechain NH_2	Ser16	Backbone O	Hydrogen bond
Tyr1087	Backbone O	Tyr83	Sidechain OH	Hydrogen bond
**Asn1089**	Sidechain NH_2	Gly169	Backbone O	Hydrogen bond
Pro1117	Sidechain	Gly14	Backbone	Hydrophobic
Pro1117	Sidechain	Gly15	Backbone	Hydrophobic
Asp1119	Backbone O	Arg13	Sidechain N	Hydrogen bond
**Thr1120**	Sidechain OH	Arg13	Backbone O	Hydrogen bond
**His1121**	Sidechain N	Thr85	Sidechain OH	Hydrogen bond
**Lys1024**	Sidechain NH_3	Glu111	Sidechain O	Hydrogen bond

Table S3. Kinetic parameters derived from SPR experiments on binding between ICAM-1^{D1D5} and PF11_0521 DBLβ3_D4 wild type or mutants, *related to Figure 2*

Colors correspond to PF11_0521 DBLβ3_D4 residues as color-coded in Figure 1 of the main text.

Interaction	***k_a (x10^4 M^{-1} s^{-1})***	***k_d (x10^{-5} s^{-1})***	***K_D (nM)***	***R_{max} (RU)***
ICAM-1^{D1D2}::PF11_0521 DBLβ **wt**	1.93	6.2	3.2	1062
ICAM-1^{D1D2}::PF11_0521 DBLβ **M1071S**	1.84	45.1	24.6	995
ICAM-1^{D1D2}::PF11_0521 DBLβ **R1077A**	1.10	38.2	34.7	1062
ICAM-1^{D1D2}::PF11_0521 DBLβ **I1078S**	1.88	2349.0	1250.0	654
ICAM-1^{D1D2}::PF11_0521 DBLβ **N1082A**	1.84	2437.0	1320.0	551
ICAM-1^{D1D2}::PF11_0521 DBLβ **N1089A**	1.79	11.4	6.3	1127
ICAM-1^{D1D2}::PF11_0521 DBLβ **T1120A**	1.41	29.8	21.1	1168
ICAM-1^{D1D2}::PF11_0521 DBLβ **H1121A**	0.61	592.2	962.0	330
ICAM-1^{D1D2}::PF11_0521 DBLβ **K1124A**	1.59	15.4	9.75	1137
ICAM-1^{D1D2}::PF11_0521 DBLβ **G1083A**	1.99	7006.0	3520.0	276
ICAM-1^{D1D2}::PF11_0521 DBLβ **G1084A**	0.97	3938.0	4060.0	583
ICAM-1^{D1D2}::PF11_0521 DBLβ **G1111A**	1.87	63.9	34.1	1048
ICAM-1^{D1D2}::PF11_0521 DBLβ **P1116A**	0.13	236.6	1690.0	1409

Table S4. The ICAM-1 binding motif is commonly found in *P. falciparum* genomes, *related to Figures 2 and 3*

Due to its size, this Table is added as a separate file.

Table S5. Clinical data related to profiling of *P. falciparum var* gene expression in children admitted to hospital with malaria, *related to Figures 4 and 7*

CM: cerebral malaria: Blantyre coma score (BCS) <3 and no other obvious clinical course for the coma; SA: severe anemia: hemoglobin <5g/dL and BCS >2; OM: other malaria, children admitted to hospital with BCS >2 and hemoglobin >5g/L

Clinical characteristics (Figure 4C)		Gr. 1 CM	Gr. 2 SA	Gr. 3 OM
Country of origin	Benin	n= 34	n= 9	n= 16
	Ghana	n= 6	n= 2	n= 9
	Tanzania	n= 13	n= 15	n= 11
Age years		3.2 (2.5; 4.8)[a]	3.7 (2.1; 4.1)	2.5 (1.6; 3.6)
		5.0 (4.7; 5.3)	2.5 (2.0; 3.0)	4.8 (4.5; 5.6)
		2.3 (1.4; 4.0)	1.0 (0.4; 2.0)	2.8 (2.2; 3.8)
Blantyre coma score		2 (2; 2)	5 (5; 5)	5 (5; 5)
		1 (0; 2)	5 (5; 5)	5 (3; 5)
		1 (0; 2)	5 (5; 5)	5 (5; 5)
Haemoglobin (g/dl)		6.7 (4.8; 7.2)	4.3 (2.9; 4.3)	7.0 (5.8; 8.9)
		7.4 (7.0; 7.6)	4.8 (4.7; 4.9)	10.5 (6.6; 11.7)
		6.6 (6.1; 8.6)	4.2 (3.3; 4.6)	11.0 (10.7; 11.8)
Parasites per μl (x1000)		36.9 (9.3; 128.0)	40.7 (9.3; 116.0)	179.4 (23.0; 496.0)
		71.4 (10.7; 96.2)	56.4 (21.5; 91.3)	77.5 (13.7; 182.9)
		31.6 (3.3; 57.4)	50.4 (7.6; 86.5)	68.8 (20.1; 78.5)

Clinical characteristics (Figure 7A and 7B)		Gr. 1 CM	Gr. 2 SA	Gr. 3 OM
Country of origin	Tanzania	n= 14	n= 21	n= 10
Age years		1.8 (1.3; 4.0)	2.2 (1.3; 2.4)	2.7 (1.5;3.2)
Blantyre coma score		2 (1; 2)	5 (5; 5)	5 (5; 5)
Haemoglobin (g/dl)		6.5 (4.6; 8.0)	3.9 (3.7; 4.4)	6.7 (5.1;8.0)
Parasites per μl (x1000)		79.1 (56.6; 144.6)	30.3 (12.6; 113.4)	35.2 (16.1; 75.7)

[a] Median (25%;75% percentiles).

Table S6. Kinetic parameters derived from SPR experiments on binding between PfEMP1 head structures and EPCR and/or ICAM-1^{D1D5}, *related to Figure 5*

Ligand	*Analyte*	k_a ($x10^4\ M^{-1}\ s^{-1}$)	k_d ($x10^{-5}\ s^{-1}$)	K_D (nM)	R_{max} (RU)
EPCR	**PF11_0521 HS**	3.81	4.50	1.18	2344
ICAM^{D1D5}	**PF11_0521 HS**	2.32	5.41	2.33	1533
ICAM^{D1D5}:: **PF11_0521 HS**	EPCR	1.72	19.50	11.40	228
EPCR	**DD2var32 HS**	16.20	1.29	0.79	2685
ICAM^{D1D5}	**DD2var32 HS**	0.55	1.14	20.60	1733
ICAM^{D1D5}:: **Dd2var32 HS**	EPCR	8.88	4.52	5.09	74
EPCR	**PFD1235w HS**	3.40	58.1	17.1	1164
ICAM^{D1D5}	**PFD1235w HS**	0.60	10.8	18.0	1093
ICAM^{D1D5}:: **PFD1235w HS**	EPCR	0.36	102.9	270.9	299

HS = head structure

Table S7. Statistical analysis of the effect of receptor and shear stress on adhesion of HB3VAR03, 3D7*PFD1235w*, and IT4VAR13 infected erythrocytes under physiological flow conditions and primers used for amplification of PfEMP1-specific DNA sequences and recombinant proteins produced, *related to Figures 1,2,3,4,5 and 6.*

Due to its size, this Table is added as a separate file.

Supplemental Experimental Procedures

Production of recombinant proteins and peptides

His-tagged DBLβ domains and a head structure of PFD1235w (CIDRα1.6- DBLβ3) were expressed in *E. coli* Shuffle C3030 cells (New England Biolabs) from DNA constructs generated by PCR from genomic DNA (using the primers in Table S7C) (Bengtsson et al., 2013b). A hybrid of PFD1235w DBLβ3_D4 (aa 739-1,221) and PFD1235w DBLβ3_D5 (aa 1,239-1,689) termed D5_motif was cloned by overlapping PCR amplification and expressed in *E. coli* Shuffle C3030 cells. All proteins were purified by immobilised metal ion affinity chromatography. The wild type PF11_0521 DBLβ3_D4 domain used for crystallization was further purified by ion exchange chromatography on a HiTrap S FF column (GE Healthcare) and by size exclusion chromatography using a HiLoad Superdex 75 16/60 column (GE Healthcare).

Mutants of PF11_0521 DBLβ3_D4 were generated by site-directed mutagenesis using the Quikchange protocol (Agilent Technologies). Structural mutations in the ICAM-1 binding site of PF11_0521 DBLβ3_D4 were generated using sets of 5'phosphorylated primers carrying the respective mutations. These were used to amplify a vector encoding DBLβ3_D4, followed by circularization through blunt-end ligation with T4 Ligase (Life Technologies). Mutations were verified by sequencing and the mutants were expressed and purified as described above. Triple domain head structures of PF11_0521 (NTS-DBLα1.7-CIDRα1.4-DBLβ3) and of Dd2var32 (NTS-DBLα1.7-CIDRα1.4-DBLβ1) were produced in insect cells as described (Lavstsen et al., 2012).

Recombinant ICAM-1-Fc (ICAM-1^{D1D5} and ICAM-1^{D1D2}) were expressed in HEK cells and COS-7 cells, respectively (Bengtsson et al., 2013a; Brown et al., 2013). ICAM-1^{D1D2} used for crystallization was transiently expressed as a C-terminal hexa-histidine-tagged protein in 293F cells (Life Technologies). Kifunensine (Cayman Chemical) was added directly after transfection to a final concentration of 1.5 μM. One day later, the medium was further supplemented with valproic acid (Sigma-Aldrich) at a final concentration of 2.2 mM. Seven days after transfection, the cell supernatant was harvested, sterile-filtered, concentrated, and ICAM-1^{D1D2} was purified by immobilised metal ion affinity chromatography using a NiNTA column (Qiagen), followed by size exclusion chromatography on a HiLoad Superdex 75 16/60 column (GE Healthcare). ICAM-1^{D1D5}-Fc used for SAXS experiments was cleaved with endoproteinase GluC (New England Biolabs) and the Fc tag was removed using a HiTrap Protein A HP column (GE Healthcare). Recombinant His-tagged EPCR for use in SPR experiments was expressed in S2 cells, purified and biotinylated as described (Lau et al., 2015). Recombinant EPCR used for infected erythrocyte (IE) adhesion assays was produced as described in (Nunes-Silva et al., 2015). The motif peptide (derived from PFD1235w DBLβ_D4) LYAKARIVASNGGPGYYNTEVQKKDRSVYDFLYELHLQNGGKKGPPPATHPYKSVN TRDKRDATDDTTP was produced by Schaefer-N, Denmark.

Crystallization

Purified PF11_0521 DBLβ3_D4 domain was mixed with a 1.5 molar excess of purified ICAM-1^{D1D2} and the complex was further purified and buffer exchanged by size exclusion chromatography on a HiLoad Superdex 75 16/60 column (GE Healthcare) into 10 mM HEPES, 150mM NaCl, pH 7.2. Fractions containing pure complex were pooled and concentrated to 19.1

mg/ml. Crystals were grown through vapour diffusion in sitting drops by mixing 100 nL of protein solution with 100 nL of well solution. Initial crystals grew as clusters of needles at 277 K in conditions from the Morpheus screen (Molecular Dimensions) with 10% (w/v) PEG 20000, 20% (v/v) PEG 500 and 0.1M Tris-BICINE (pH 8.5) as well solution. Crystallization conditions were further optimized by mixing 100 nL of protein solution with 50 nL of well solution and 50 nL of additive solution (Silver Bullets, Hampton Research). For cryoprotection and storage, crystals were transferred into a drop of well solution containing 16% (w/v) PEG 20000, 32% (v/v) PEG 500, 0.1 M Tris-BICINE (pH 8.5) and flash-frozen in liquid Nitrogen.

Data collection, phasing and refinement

Data were collected at beamline IO2 (Diamond Light Source, UK) using x-rays of 0.9795 Å wavelength with a Pilatus 6M detector (Dectris, Baden-Daettwil, Switzerland). The data were processed with the CCP4 program suite (Winn et al., 2011) using iMOSFLM (Battye et al., 2011) for indexing and Scala (Evans, 2006) for scaling. For molecular replacement, a search model was prepared with chainsaw using the DBLα domain of the varO PfEMP1 (PDB code 2XU0) (Juillerat et al., 2011) as template, and this model was further trimmed by removing all loops, leaving only the conserved α-helical core of the DBL domain (Higgins and Carrington, 2014). Molecular replacement with Phaser-MR (McCoy et al., 2007) using the trimmed search model as well as the individual D1 and D2 domains from ICAM-1^{D1D2} (PDB code 1IC1) (Casasnovas et al., 1998) identified one copy of the PF11_0521 DBLβ3_D4:ICAM-1^{D1D2} complex in the asymmetric unit. To minimise the effects of model bias, the initial molecular replacement solution was subjected to a round of simulated annealing using the PHENIX program suite (Adams et al., 2010). The remaining parts of the model were built through iterative

cycles of refinement using Refmac5 (Murshudov et al., 2011), Buster (Bricogne G. and Roversi P, 2016) and model building in Coot (Emsley et al., 2010). All structure figures were prepared using Pymol (Schroedinger).

Small angle x-ray scattering (SAXS)

SAXS experiments were conducted at the BM29 beamline at the European Synchrotron Radiation Facility (Grenoble, France). Data were collected at a wavelength of 0.9919 Å using a Pilatus 1M detector (Dectris, Baden-Daettwil, Switzerland) with a sample-detector distance of 2.867 m. All samples for SAXS were purified and buffer-exchanged by size-exclusion chromatography into 20 mM HEPES, 150 mM NaCl, pH 7.4. The sample purity was checked by SDS-PAGE and only samples with a purity of at least 95% were used for SAXS experiments. Prior to measurements, all samples were filtered through a 0.1 μm centrifugal spin-filter (Millipore). Data for each sample were collected at 20°C, and before and after each sample buffer was measured for background subtraction.

For data analysis, scattering curves were manually inspected in PRIMUS (Konarev et al., 2003). Frames showing signs of radiation damage were omitted from further analysis. Remaining scattering curves were averaged, and averaged buffer curves were subtracted. The radius of gyration (R_g) was determined by Guinier analysis using AutoRg in PRIMUS. GNOM (Svergun, 1992) was used to determine the pair-distance distribution function P(r) and the maximum particle diameter (D_{max}).

To generate volumetric representations based on the SAXS data, 20 independent *ab initio* bead models were calculated using DAMMIF (Franke and Svergun, 2009) with default parameters. These models were then averaged using DAMAVER (Volkov and Svergun, 2003), and the averaged model was further refined against the original data using DAMMIN. Envelopes were calculated from the refined bead models using Situs. The crystal structures of PF11_0521 DBLβ3_D4, the PF11_0521 DBLβ3_D4:ICAM-1^{D1D2} complex as well as ICAM-1^{D3D5} (PDB code 2OZ4) (Chen et al., 2007) were docked into these envelopes using Sculptor (Birmanns et al., 2011). CRYSOL (Svergun et al., 1995) was used for fitting the experimental solution scattering data of the PF11_0521 DBLβ3_D4:ICAM-1^{D1D2} complex to the theoretical scattering based on the crystal structure.

Surface plasmon resonance (SPR)

SPR experiments were carried out on a Biacore T-200 instrument (GE Healthcare). For analysis of wildtype and mutant PF11_0521 DBLβ3_D4 binding to ICAM-1, ICAM-1^{D1D5} Fc was immobilized to 2100 response units (RU) on a CM5 chip (GE Healthcare) pre-coupled to Protein A. Wild type and mutant PF11_0521 DBLβ3 constructs were buffer-exchanged into 10 mM HEPES pH 8.0, 300 mM NaCl, 0.05% Tween-20. Concentration series (two-fold dilution from 4 nM to 500 nM) were flowed at 40 μL/min over the chip with 240 s association and 660 s dissociation times. After each run, the chip was regenerated by injecting 10 mM Glycine, pH 2.0 for 120 s at 10 μL/min.

For analysis of binding of PfEMP1 head structures to EPCR, biotinylated EPCR was immobilized to 650 RU on a CAP chip using the Biotin Capture Kit (GE Healthcare). All head

structures were buffer-exchanged into 10 mM HEPES (pH 7.4), 300 mM NaCl, 0.05% Tween-20, 2 mg/ml ssDNA (Panreac AppliChem). Concentration series (two-fold dilution from 500 nM to 4 nM) were flowed over the chip at 30 μL/min with 240 s association and 720 s dissociation times. The chip was regenerated between runs by injecting CAPture Kit regeneration solution (GE Healthcare) for 120 s at 10 μL/min.

To measure binding of PfEMP1 head structures to ICAM-1, ICAM-1^{D1D5} Fc was immobilized to 1,400 RU on a CM5 chip (GE Healthcare) pre-coupled to Protein A. All head structures were buffer-exchanged into 10 mM HEPES (pH 8.0), 300 mM NaCl, 0.05% Tween-20. Concentration series (two-fold dilution from 4 nM to 500 nM) were flowed at 40 μL/min over the chip with 240 s association and 600 s dissociation times. The chip was regenerated between runs by injecting 10 mM Glycine (pH 2.0) for 120 s at 10 μL/min.

For analysis of dual adhesion of PfEMP1 head structures to ICAM-1 and EPCR, ICAM-1^{D1D5} Fc was immobilized to 1,800 RU (for experiments with PF11_0521 head structure), 2,100 RU (for experiments with Dd2var32 head structure) or 1,500 RU (for experiments with PFD1235w head structure) on a CM5 chip (GE Healthcare) pre-coupled to Protein A. All head structures were buffer-exchanged into 10 mM HEPES (pH 8.0), 300 mM NaCl, 0.05% Tween-20, and were then injected over the ICAM-1 immobilized on the chip at a fixed concentration of 250 nM for 240 s at 20 μL/min. Then, a concentration series (two-fold dilution from 250 nM to 16 nM) of EPCR (buffer-exchanged into 10 mM HEPES (pH 8.0), 300 mM NaCl, 0.05% Tween-20) was flowed over the chip at 30 μL/min with 240 s association and 720 s dissociation times. After each run, the chip was regenerated by injecting 10 mM Glycine (pH 2.0) for 120 s at 10 μL/min.

SPR data were analysed using the BIAevaluation software 2.0.3 (GE Healthcare). Values for k_a, k_d and K_D were determined by globally fitting curves corresponding to at least 5 concentrations into a one-site kinetic model. All SPR experiments were performed in duplicates, and curves shown are representatives of these measurements.

Circular Dichroism Spectroscopy

All circular dichroism (CD) spectroscopy experiments were conducted on a J-815 Spectropolarimeter, connected to a Peltier temperature control unit. The samples were dialyzed against 100mM sodium phosphate buffer, 200mM NaF, pH 7.2 and diluted to 0.3 mg/ml. CD spectra and spectra for thermal denaturation assays were recorded as described previously (Lennartz et al., 2015).

Patient samples

P. falciparum isolates were collected from malaria patients (Table S5) for transcription analysis. This was performed at Hohoe Municipal Hospital in the Volta Region of Ghana (Stevenson et al., 2015), at Korogwe District Hospital in Korogwe District in Northeastern Tanzania (Lavstsen et al., 2012), and at Centre National Hospitalier Universitaire Hubert Koutoucou Mega in Cotonou, Benin (Moussiliou et al., 2015). The studies were approved by the Ethical Review Committee of the Ghana Health Services (file GHS-ERC 08/05/14), the Ethical Review Board of the National Institute for Medical Research, Tanzania (National Institute of Medical Research/HQ/R.8a/Vol.IX/559), and by the Ethics Committee of the Research Institute of Applied Biomedical Sciences, Cotonou, Benin (N°21/CER/ISBA/13), respectively.

After informed consent had been obtained from a parent or a guardian, enrolled children were subjected to clinical investigation, collection of venous blood samples, and treatment according to national guidelines. Pelleted erythrocytes were dissolved in 9 volumes of Trizol reagent (Thermo Fisher Scientific) for RNA preservation at minus 80°C.

Clinical manifestations of malaria were classified according to the definitions and associated criteria of the World Health Organization. Patients were categorized as having cerebral malaria (CM) if they had a positive blood smear of the asexual form of *P. falciparum*, unrousable coma (Blantyre coma score, BCS ≤ 2) with exclusion of other causes of coma and severe illness. Patients were classified as having severe malarial anemia (SA) if hemoglobin < 5g/dL and BCS >2. Patients were classified as having other malaria (OM) if BCS >2 and hemoglobin >5 g/dL.

Additional samples used were, plasma samples from 76 *P. falciparum*-exposed Tanzanian children (1-17 years) collected during a cross-sectional study monitoring risk factors for anaemia and febrile malaria and nine plasma samples from *P. falciparum*- exposed adult Liberians. These samples were from previous studies described in (Lusingu et al., 2004; Theisen et al., 2000).

Antibodies

Rat immunizations and IgG preparations were done as described (Bengtsson et al., 2013b). All experimental animal procedures were approved by The Danish Animal Procedures Committee ("Dyreforsøgstilsynet") as described in permit no. 2008/561-1498 and according to the guidelines described in Danish act LBK 1306 (23/11/2007) and BEK 1273 (12/12/2005). DBLβ

and motif-specific rat IgG (anti-PFD1235w_D4; anti-PFD1235w_motif; anti-IT4VAR13) were affinity purified from rat antisera as described (Joergensen et al., 2010). Pre-immunization serum or rat IgG (Sigma Aldrich) were used as negative controls in binding assays. Pooled plasma from nine *P. falciparum*-exposed adult Liberians (Theisen et al., 2000) were used for affinity purification of DBLβ- and motif-specific human IgG (anti-PFD1235w_D4; anti-PFD1235w_motif; anti-IT4VAR13). Human IgG (Sigma Aldrich) was used as negative control in binding assays.

ELISA

ICAM-1 binding was assessed by ELISA using Maxisorp plates coated with 26 different recombinant DBLβ proteins (2-10 μg/ml) (Bengtsson et al., 2013b). Of these, eight DBLβ proteins had known ability to bind ICAM-1 (Bengtsson et al., 2013b), but were identified here as having the motif prior to doing the ELISA. Additionally eight DBLβ proteins were predicted as ICAM-1 binders and ten as non-ICAM-1 binders based on sequence analysis done in this study prior to doing ELISA. An additional eight DBLβ proteins without the motif were previously tested for ICAM-1 binding in the laboratory (Figure 3A). Binding was detected using rabbit anti-human IgG-HRP (Dako; 1:3,000) targeting the Fc-portion of the ICAM-1-Fc.

ELISA was similarly used to analyze motif-specific IgG reactivity and the ICAM-1 anti-adhesive effect of individual human immune plasma (1:100) from *P. falciparum*-exposed individuals (1 - 17 years) living in an area of high parasite transmission in northeastern Tanzania (Lusingu et al., 2004). Bound human IgG was detected using rabbit anti-human IgG as described above. For adhesion inhibition assays using affinity purified rat or human IgG (0.25-32 μg/ml) dilutions

were added to ICAM-1-Fc-coated plates simultaneously with DBLβ protein. A HRP-conjugated penta-His antibody (Qiagen; 1:3,000) was used for detection of bound DBLβ.

Plasmodium falciparum parasite culture

The *P. falciparum* clones 3D7, HB3, and IT4 were maintained *in vitro* and selected with antibodies to express specific PfEMP1 proteins as described (Bengtsson et al., 2013b; Joergensen et al., 2010). *P. falciparum* 3D7 infected erythrocytes (IEs) were selected with the human monoclonal antibody AB01, which targets PFD1235w DBLγ13_D6 (Barfod et al., 2011) or with a polyclonal rat anti-serum against PF11_0008 CIDRβ4_D6. *P. falciparum* IT4 IEs were similarly selected using a rat anti-serum against IT4VAR13 DBLβ5_D4, whereas *P. falciparum* HB3 IEs were antibody-selected using a rat anti-serum against HB3VAR03 DBLβ3_D4. In all cases, PfEMP1 expression was regularly monitored by flow cytometry using PfEMP1-specific antisera, and only cultures with more than 60% antibody-labeled IEs were used in the study. The identity and clonality of all parasites used were routinely verified by genotyping as described (Snounou et al., 1999). Mycoplasma infection was excluded regularly using the MycoAlert Mycoplasma Detection Kit (Lonza) according to the manufacturer's instructions.

Flow Cytometry

P. falciparum IEs were DNA-labeled with ethidium bromide and surface-labeled with rat antibodies as described (Joergensen et al., 2010). Rat antisera (anti-PFD1235w_D4; anti-HB3VAR03_D4; anti-PF11_0008_D6; anti-IT4VAR13) were used at a 1:40 dilution. IE-bound antibodies were labeled using FITC-conjugated secondary rabbit anti-rat IgG (1:150, Vector Labs). FITC fluorescence data from ethidium bromide-positive cells were collected on a

Cytometic FC500 MPL flow cytometer (Beckman Coulter) and data analysed using WinList version 6.0 (Verity Software House).

IE adhesion to recombinant receptors

Microslides ($VI^{0.1}$; Ibidi) were coated overnight with recombinant ICAM-1-Fc (50 µg/ml) (Bengtsson et al., 2013a), EPCR-his (10 µg/ml) (Nunes-Silva et al., 2015) or with recombinant CD36 (20 µg/ml, R&D Systems). For endothelial adhesion assays, human brain microvascular endothelial cells (HBMEC at passage 2-5; Sciencell) were seeded onto fibronectin (2 $\mu g/cm^{2;}$ Millipore) pre-coated venaEC ($5x10^5$ cells/ml) or microslides ($VI^{0.4}$; ibidi. $3x10^5$ cells/ml) for 72 – 96 hours. Activation of HBMEC was done by adding TNFα (10 ng/ml; Sinobiological) for 18 hours. Mature IEs were adjusted to 3% parasitaemia in RPMI-1640 supplemented with 2% normal human serum. The IEs were then subjected to physiological shear stress ranging from 0.5-5 $dyne/cm^2$ as described (Lennartz et al., 2015). After 5 minutes of flow, the number of bound IE/mm^2 in five separate fields was counted at 20× magnification using a Leica inverted phase contrast microscope. Bound IE were dislodged after each run, and the shear stress increased to measure the ability of IE to initiate adhesion at each shear stress. A minimum of three independent experiments in triplicate were performed. To assess the capacity of antibodies to inhibit IE adhesion, the IEs were pre-incubated with affinity-purified IgG or plasma as described above. Affinity-purified non-immune rat or human IgG were included as negative controls. The specificity of IE adhesion to ICAM-1-Fc, EPCR-his, rCD36 and HBMEC was verified by pre-incubating flow channels with 40 µg/ml anti-ICAM-1 antibody (clone 15.2, AbD Serotec), 10 µg/ml anti-EPCR (polyclonal, R&D Systems) or with 20 µg/ml anti-CD36 (monoclonal, Beckman Coulter). All assays were blinded.

Immunofluorescence assay

Dual binding of IE to ICAM-1 and EPCR was assessed by incubating the IEs expressing PFD1235w, HB3VAR03, or IT4VAR13 with preformed antibody:protein complexes. Briefly, equal molar amounts of ICAM-1-Fc and goat-F(ab)$_2$-anti-human IgG-Fc Dylight 488 (Abcam) or EPCR-his with mouse-anti-PENTA-His (Qiagen) and anti-mouse IgG-Alexa 568 (Abcam) were incubated at 4°C for 4 hours. After incubation, complexes were aliquoted and stored at -20°C until use. IEs were washed in PBS, incubated with 2% NHS for 10 minutes at 37°C, and washed twice with 1× PBS. Aliquots of the antibody:protein complex were thawed and diluted 10x in 2% Ig-free BSA/PBS before adding to IEs and incubating at 37°C for 10 minutes with gentle shaking. IEs were then washed 3× with 2% Ig-free BSA/PBS. Thin smears were air-dried and mounted with ProLong Gold Antifade DAPI mounting medium (Life Technologies) and examined using a Zeiss AxioImager Z1 and a 63× oil inversion lens. Composite images were compiled with Zen Blue (Zeiss) and Fiji-ImageJ open source biological imaging software (Schindelin et al., 2012).

Bioinformatic analysis

A total of 151 DBLβ domains from seven previously annotated whole parasite genomes (Rask et al., 2010) were used to blastp extract DBLβ sequences from assemblies of Illumina whole-genome sequencing data, as described previously (Lau et al., 2015). This analysis included sequence data from 226 *P. falciparum* genomes from Africa and Asia (study number ERP000190), made available through the MalariaGen community (Miotto et al., 2015), and additional sequence data available through PlasmoDB, NCBI, and the Broad Institute. DBLβ

domains predicted to bind ICAM-1 were identified using the search term I[V/L]x_3N[E]GG[P/A]xYx_{27}GPPx_3H. A WebLogo 3 sequence logo (Crooks et al., 2004) (http://weblogo.three plusone.com/) was generated based on alignment of 145 protein sequences with the ICAM-1 binding motif (Table S4). The average amino acid identity of DBLβ domains was calculated using the Praline multiple sequence alignment tool (http://www.ibi.vu.nl/programs/pralinewww/) using default settings.

Frequencies and distribution of motif-containing DBLβ domains in relation to PfEMP1 group and adjacent CIDR domain types was estimated from counting domain co-occurences in the *var* sequence contigs assembled from the 233 *P. falciparum* genomes. Multiple alignments were made using MUSCLE v. 3.7 software (Edgar, 2004). Relatedness of DBLβ domains and subdomain 3 sequence was inferred by using the Maximum Likelihood method based on the Whelan And Goldman plus Freq. model (Whelan and Goldman, 2001) using MEGA6 (Tamura et al., 2007).The tree with the highest log likelihood (Figure 3A (-35460.7853) and Figure 3B (-179728.3832)) is shown. Initial tree(s) for the heuristic search were obtained automatically by applying Neighbor-Join and BioNJ algorithms to a matrix of pairwise distances estimated using a JTT model, and then selecting the topology with superior log likelihood value. The trees were drawn to scale, with branch lengths measured in the number of substitutions per site. The analysis in Figure 3A involved 55 amino acid sequences with a total of 664 positions in the final dataset. In Figure 3B, a discrete Gamma distribution was used to model evolutionary rate differences among sites (3 categories (+G, parameter = 0.8099)). The rate variation model allowed for some sites to be evolutionarily invariable ([+I], 5.9495% sites). All positions with less than 95% site coverage were eliminated. That is, fewer than 5% alignment gaps, missing

data, and ambiguous bases were allowed at any position. There were a total of 145 positions in the final dataset.

Amplicons representing *var* genes from eight different Ghanaian *P. falciparum* isolates (Nielsen et al., 2002) were amplified by PCR using primer combination M3637 (5′-TGCTNKTAATGGTGGTCC-3′)/Exon2 LNA (Lavstsen et al., 2012) or varF_dg2/Exon2 LNA (both (Lavstsen et al., 2012)), fragmented by sonication, built into sequencing libraries and sequenced with a 250 bp paired-end protocol on an Illumina® MiSeq. The domains of new PfEMP1 sequences were classified using the Hidden Markov Model approach, and named as described previously (Rask et al., 2010).

Additional *var* gene sequence data from 45 Tanzanian children with severe or uncomplicated *P. falciparum* malaria (Jespersen et al., 2016) were analyzed using the ICAM-1 binding motif presented here. Briefly, profiles of expressed *var* genes were obtained using the DBLα-tag PCR method described elsewhere (Lavstsen et al., 2012). The top six most abundant *var* tags (accounting for an average of 74% [range 38% to 100%] of all expressed *var* genes) in each patient were targeted for further analysis. Specific primers were designed for the relevant DBLα-tag sequences and used for long-range PCR in combination with a universal primer annealing in Exon 2 (Lavstsen et al., 2012). The resulting PCR products were Illumina-sequenced, assembled, and annotated. RT-qPCR measurements using specific primers were subsequently done to control for any potential amplification bias. For each patient, the relative abundance of each of the top-six *var* genes was expressed as a percentage of all the top-six *var* genes in that patient. Following this, normalized relative abundances were adjusted according to the relative

abundance measured by RT-qPCR. This method may in a few patients miss highly expressed transcript variants due to primer amplification bias, and is less suited for quantification of the total level of a given transcript trait within a patient, or patient group, than qPCR. However, in contrast to qPCR, using primers targeting pre-defined loci, the method allows subsequent sequence analysis of expressed genes. From this identification and based on the relative transcript abundance of these highly transcribed *var* genes, the percentage of transcripts coding for motif-containing ICAM-1-binding DBLβ and EPCR-binding CIDRα1 was determined in each of the 45 children.

Real-time quantitative PCR (RT-qPCR)

Parasite RNA from clinical *P. falciparum* isolates was reverse-transcribed into cDNA and subjected to RT-qPCR using primer pairs (Table S7C) targeting *var* gene sub-classes encoding group A DBLβ domains predicted to bind to ICAM-1 (motif: primers Q183/Q186) and/or *var* gene sub-classes encoding EPCR-binding CIDRα1 domains (EPCR: primers "DBLa2/1.1/2/4/7", Makumbaye et al. submitted). Quanti-Tect SYBR Green PCR Master Mix (Qiagen) and a Rotorgene thermal cycle system (Corbett Research) were used as described (Jensen et al., 2004). The abundance of *var* gene transcripts was calculated relative to the transcript level of the endogenous control gene Seryl-tRNA synthetase ($\Delta Ct_{var\_gene} = Ct_{var\_gene} - Ct_{endogenous\ control\ gene}$). Delta Ct (*var* gene) values were translated into transcription units (Tu) as described (Lavstsen et al., 2012). Values of $\Delta Ct_{var\_gene} \geq 5$ were assigned a value of 5 and Tu calculated as $Tu = 2^{(5-\Delta Ct_{var\_gene})}$. Thus, ΔCt_{var_gene} values ≥5 were assigned a Tu-value of 1 with ΔCt_{var_gene} values of 4 corresponding to Tu = 2; $\Delta Ct_{var\_gene} = 3$:⇔ Tu =4; $\Delta Ct_{var\_gene} = 2$:⇔ Tu =8; $\Delta Ct_{var\_gene} = 1$:⇔

Tu = 16; $\Delta Ct_{var\_gene} = 0$:⇔ Tu = 32 corresponding to the mean level of the transcription of the endogenous control gene.

Statistical analysis

Patients were assigned to three clinical groups: i) children with a Blantyre score of <3 (CM); ii) children with a Blantyre score >2 and hemoglobin <5 g/dL (SA); iii) *P. falciparum* positive children with a Blantyre score >2 and hemoglobin > 5 g/dL (other malaria, OM). *Var* gene transcript levels (Figure 4C) and percentages of transcripts (Figure 7) were compared using the Wilcoxon-Mann-Whitney rank sum test. ICAM-1 transcript levels (Figure 4C) showed no statistical significant difference between SA and OM children.

Transcription data from these two groups were therefore pooled and compared to the data from group i. To analyze if study site (Figure 4C) influenced the association between ICAM-1 transcript levels and clinical outcome of infection a comparison was done using a logistic regression model with cerebral malaria as outcome variable and study site (Benin, Ghana, and Tanzania) as well as ICAM-1 transcript level as explanatory variables. Inhibition of ICAM-1 binding by antibodies (Figure 4B) was compared using the Wilcoxon-Mann-Whitney rank sum test using SigmaPlot 13.0 (Systat Software Inc, United Kingdom), all other analysis presented were done using STATA14 software (StataCorp, USA).

Supplemental References

Adams, P.D., Afonine, P.V., Bunkoczi, G., Chen, V.B., Davis, I.W., Echols, N., Headd, J.J., Hung, L.W., Kapral, G.J., Grosse-Kunstleve, R.W., *et al.* (2010). PHENIX: a comprehensive Python-based system for macromolecular structure solution. Acta Crystallogr D Biol Crystallogr *66*, 213-221.

Avril, M., Bernabeu, M., Benjamin, M., Brazier, A.J., and Smith, J.D. (2016). Interaction between Endothelial Protein C Receptor and Intercellular Adhesion Molecule 1 to Mediate Binding of Plasmodium falciparum-Infected Erythrocytes to Endothelial Cells. MBio *7*.

Avril, M., Tripathi, A.K., Brazier, A.J., Andisi, C., Janes, J.H., Soma, V.L., Sullivan, D.J., Jr., Bull, P.C., Stins, M.F., and Smith, J.D. (2012). A restricted subset of var genes mediates adherence of Plasmodium falciparum-infected erythrocytes to brain endothelial cells. ProcNatlAcadSciUSA *109*, E1782-E1790.

Barfod, L., Dalgaard, M.B., Pleman, S.T., Ofori, M.F., Pleass, R.J., and Hviid, L. (2011). Evasion of immunity to Plasmodium falciparum malaria by IgM masking of protective IgG epitopes in infected erythrocyte surface-exposed PfEMP1. ProcNatlAcadSciUSA *108*, 12485-12490.

Battye, T.G., Kontogiannis, L., Johnson, O., Powell, H.R., and Leslie, A.G. (2011). iMOSFLM: a new graphical interface for diffraction-image processing with MOSFLM. Acta Crystallogr D Biol Crystallogr *67*, 271-281.

Bengtsson, A., Joergensen, L., Barbati, Z.R., Craig, A., Hviid, L., and Jensen, A.T. (2013a). Transfected HEK293 cells expressing functional recombinant intercellular adhesion molecule 1 (ICAM-1)--a receptor associated with severe Plasmodium falciparum malaria. PLoSOne *8*, e69999.

Bengtsson, A., Joergensen, L., Rask, T.S., Olsen, R.W., Andersen, M.A., Turner, L., Theander, T.G., Hviid, L., Higgins, M.K., Craig, A., *et al.* (2013b). A novel domain cassette identifies Plasmodium falciparum PfEMP1 proteins binding ICAM-1 and is a target of cross-reactive, adhesion-inhibitory antibodies. JImmunol *190*, 240-249.

Bernabeu, M., Danziger, S.A., Avril, M., Vaz, M., Babar, P.H., Brazier, A.J., Herricks, T., Maki, J.N., Pereira, L., Mascarenhas, A., *et al.* (2016). Severe adult malaria is associated with specific PfEMP1 adhesion types and high parasite biomass. Proc Natl Acad Sci U S A *113*, E3270-3279.

Birbeck, G.L., Molyneux, M.E., Kaplan, P.W., Seydel, K.B., Chimalizeni, Y.F., Kawaza, K., and Taylor, T.E. (2010). Blantyre Malaria Project Epilepsy Study (BMPES) of neurological outcomes in retinopathy-positive paediatric cerebral malaria survivors: a prospective cohort study. Lancet Neurol *9*, 1173-1181.

Birmanns, S., Rusu, M., and Wriggers, W. (2011). Using Sculptor and Situs for simultaneous assembly of atomic components into low-resolution shapes. J Struct Biol *173*, 428-435.

Bricogne G., B.E., Brandl M., Flensburg C., Keller P., Paciorek W.,, and Roversi P, S.A., Smart O.S., Vonrhein C., Womack T.O. (2016). BUSTER version 2.10.2. Global Phasing Ltd Cambridge, UK.

Brown, A., Turner, L., Christoffersen, S., Andrews, K.A., Szestak, T., Zhao, Y., Larsen, S., Craig, A.G., and Higgins, M.K. (2013). Molecular architecture of a complex between an adhesion protein from the malaria parasite and intracellular adhesion molecule 1. JBiolChem *288*, 5992-6003.

Casasnovas, J.M., Stehle, T., Liu, J.H., Wang, J.H., and Springer, T.A. (1998). A dimeric crystal structure for the N-terminal two domains of intercellular adhesion molecule-1. Proc Natl Acad Sci U S A *95*, 4134-4139.

Chen, X., Kim, T.D., Carman, C.V., Mi, L.Z., Song, G., and Springer, T.A. (2007). Structural plasticity in Ig superfamily domain 4 of ICAM-1 mediates cell surface dimerization. Proc Natl Acad Sci U S A *104*, 15358-15363.

Claessens, A., Adams, Y., Ghumra, A., Lindergard, G., Buchan, C.C., Andisi, C., Bull, P.C., Mok, S., Gupta, A.P., Wang, C.W., *et al.* (2012). A subset of group A-like var genes encodes the malaria parasite ligands for binding to human brain endothelial cells. Proc Natl Acad Sci U S A *109*, E1772-1781.

Crooks, G.E., Hon, G., Chandonia, J.M., and Brenner, S.E. (2004). WebLogo: a sequence logo generator. Genome Res *14*, 1188-1190.

Cunnington, A.J., Riley, E.M., and Walther, M. (2013). Stuck in a rut? Reconsidering the role of parasite sequestration in severe malaria syndromes. Trends Parasitol *29*, 585-592.

Dondorp, A.M., Fanello, C.I., Hendriksen, I.C., Gomes, E., Seni, A., Chhaganlal, K.D., Bojang, K., Olaosebikan, R., Anunobi, N., Maitland, K., *et al.* (2010). Artesunate versus quinine in the treatment of severe falciparum malaria in African children (AQUAMAT): an open-label, randomised trial. Lancet *376*, 1647-1657.

Doumbo, O.K., Thera, M.A., Kone, A.K., Raza, A., Tempest, L.J., Lyke, K.E., Plowe, C.V., and Rowe, J.A. (2009). High levels of Plasmodium falciparum rosetting in all clinical forms of severe malaria in African children. Am J Trop Med Hyg *81*, 987-993.

Edgar, R.C. (2004). MUSCLE: multiple sequence alignment with high accuracy and high throughput. Nucleic Acids Res *32*, 1792-1797.

Emsley, P., Lohkamp, B., Scott, W.G., and Cowtan, K. (2010). Features and development of Coot. Acta Crystallogr D Biol Crystallogr *66*, 486-501.

Evans, P. (2006). Scaling and assessment of data quality. Acta Crystallogr D Biol Crystallogr *62*, 72-82.

Franke, D., and Svergun, D.I. (2009). DAMMIF, a program for rapid ab-initio shape determination in small-angle scattering. Journal of Applied Crystallography *42*, 342-346.

Goel, S., Palmkvist, M., Moll, K., Joannin, N., Lara, P., Akhouri, R.R., Moradi, N., Ojemalm, K., Westman, M., Angeletti, D., *et al.* (2015). RIFINs are adhesins implicated in severe Plasmodium falciparum malaria. Nat Med *21*, 314-317.

Higgins, M.K., and Carrington, M. (2014). Sequence variation and structural conservation allows development of novel function and immune evasion in parasite surface protein families. Protein Sci *23*, 354-365.

Howell, D.P., Levin, E.A., Springer, A.L., Kraemer, S.M., Phippard, D.J., Schief, W.R., and Smith, J.D. (2008). Mapping a common interaction site used by *Plasmodium falciparum* Duffy binding-like domains to bind diverse host receptors. MolMicrobiol *67*, 78-87.

Hsieh, F.L., Turner, L., Bolla, J.R., Robinson, C.V., Lavstsen, T., and Higgins, M.K. (2016). The structural basis for CD36 binding by the malaria parasite. Nat Commun *7*, 12837.

Hviid, L., and Jensen, A.T. (2015). PfEMP1 - a parasite protein family of key importance in *Plasmodium falciparum* malaria immunity and pathogenesis. AdvParasitol *88*, 51-84.

Idro, R., Jenkins, N.E., and Newton, C.R. (2005). Pathogenesis, clinical features, and neurological outcome of cerebral malaria. Lancet Neurol *4*, 827-840.

Janes, J.H., Wang, C.P., Levin-Edens, E., Vigan-Womas, I., Guillotte, M., Melcher, M., Mercereau-Puijalon, O., and Smith, J.D. (2011). Investigating the host binding signature on the Plasmodium falciparum PfEMP1 protein family. PLoSPathog *7*, e1002032.

Jensen, A.T., Magistrado, P., Sharp, S., Joergensen, L., Lavstsen, T., Chiucchiuini, A., Salanti, A., Vestergaard, L.S., Lusingu, J.P., Hermsen, R., *et al.* (2004). *Plasmodium falciparum* associated with severe childhood malaria preferentially expresses PfEMP1 encoded by group A *var* genes. JExpMed *199*, 1179-1190.

Jespersen, J.S., Wang, C.W., Mkumbaye, S.I., Minja, D.T., Petersen, B., Turner, L., Petersen, J.E., Lusingu, J.P., Theander, T.G., and Lavstsen, T. (2016). Plasmodium falciparum var genes expressed in children with severe malaria encode CIDRalpha1 domains. EMBO Mol Med.

Joergensen, L., Bengtsson, D.C., Bengtsson, A., Ronander, E., Berger, S.S., Turner, L., Dalgaard, M.B., Cham, G.K., Victor, M.E., Lavstsen, T., *et al.* (2010). Surface co-expression of two different PfEMP1 antigens on single Plasmodium falciparum-infected erythrocytes facilitates binding to ICAM1 and PECAM1. PLoSPathog *6*, e1001083.

Juillerat, A., Lewit-Bentley, A., Guillotte, M., Gangnard, S., Hessel, A., Baron, B., Vigan-Womas, I., England, P., Mercereau-Puijalon, O., and Bentley, G.A. (2011). Structure of a Plasmodium falciparum PfEMP1 rosetting domain reveals a role for the N-terminal segment in heparin-mediated rosette inhibition. Proc Natl Acad Sci U S A *108*, 5243-5248.

Kim, J.H., Singvall, J., Schwarz-Linek, U., Johnson, B.J., Potts, J.R., and Hook, M. (2004). BBK32, a fibronectin binding MSCRAMM from Borrelia burgdorferi, contains a disordered region that undergoes a conformational change on ligand binding. J Biol Chem *279*, 41706-41714.

Konarev, P.V., Volkov, V.V., Sokolova, A.V., Koch, M.H.J., and Svergun, D.I. (2003). PRIMUS: a Windows PC-based system for small-angle scattering data analysis. Journal of Applied Crystallography *36*, 1277-1282.

Kwong, P.D., Wyatt, R., Robinson, J., Sweet, R.W., Sodroski, J., and Hendrickson, W.A. (1998). Structure of an HIV gp120 envelope glycoprotein in complex with the CD4 receptor and a neutralizing human antibody. Nature *393*, 648-659.

Lau, C.K., Turner, L., Jespersen, J.S., Lowe, E.D., Petersen, B., Wang, C.W., Petersen, J.E., Lusingu, J., Theander, T.G., Lavstsen, T*., et al.* (2015). Structural conservation despite huge sequence diversity allows EPCR binding by the PfEMP1 family implicated in severe childhood malaria. Cell HostMicrobe *17*, 118-129.

Lavstsen, T., Turner, L., Saguti, F., Magistrado, P., Rask, T.S., Jespersen, J.S., Wang, C.W., Berger, S.S., Baraka, V., Marquard, A.M*., et al.* (2012). Plasmodium falciparum erythrocyte membrane protein 1 domain cassettes 8 and 13 are associated with severe malaria in children. ProcNatlAcadSciUSA *109*, E1791-E1800.

Lennartz, F., Bengtsson, A., Olsen, R.W., Joergensen, L., Brown, A., Remy, L., Man, P., Forest, E., Barfod, L.K., Adams, Y*., et al.* (2015). Mapping the Binding Site of a Cross-Reactive Plasmodium falciparum PfEMP1 Monoclonal Antibody Inhibitory of ICAM-1 Binding. JImmunol *195*, 3273-3283.

Lusingu, J.P., Vestergaard, L.S., Mmbando, B.P., Drakeley, C.J., Jones, C., Akida, J., Savaeli, Z.X., Kitua, A.Y., Lemnge, M.M., and Theander, T.G. (2004). Malaria morbidity and immunity among residents of villages with different Plasmodium falciparum transmission intensity in North-Eastern Tanzania. MalarJ *3*, 26.

Malaria Genomic Epidemiology, N., Band, G., Rockett, K.A., Spencer, C.C., and Kwiatkowski, D.P. (2015). A novel locus of resistance to severe malaria in a region of ancient balancing selection. Nature *526*, 253-257.

McCoy, A.J., Grosse-Kunstleve, R.W., Adams, P.D., Winn, M.D., Storoni, L.C., and Read, R.J. (2007). Phaser crystallographic software. J Appl Crystallogr *40*, 658-674.

Menschikowski, M., Hagelgans, A., Eisenhofer, G., and Siegert, G. (2009). Regulation of endothelial protein C receptor shedding by cytokines is mediated through differential activation of MAP kinase signaling pathways. Exp Cell Res *315*, 2673-2682.

Miotto, O., Amato, R., Ashley, E.A., MacInnis, B., Almagro-Garcia, J., Amaratunga, C., Lim, P., Mead, D., Oyola, S.O., Dhorda, M*., et al.* (2015). Genetic architecture of artemisinin-resistant Plasmodium falciparum. Nat Genet *47*, 226-234.

Moussiliou, A., Alao, M.J., Denoeud-Ndam, L., Tahar, R., Ezimegnon, S., Sagbo, G., Amoussou, A., Luty, A.J.F., Deloron, P., and Ndam, N.T. (2015). High plasma levels of soluble endothelial protein C receptor are associated with increased mortality among children with cerebral malaria in Benin. JInfectDis *211*, 1484-1488.

Moxon, C.A., Heyderman, R.S., and Wassmer, S.C. (2009). Dysregulation of coagulation in cerebral malaria. Mol Biochem Parasitol *166*, 99-108.

Moxon, C.A., Wassmer, S.C., Milner, D.A., Jr., Chisala, N.V., Taylor, T.E., Seydel, K.B., Molyneux, M.E., Faragher, B., Esmon, C.T., Downey, C*., et al.* (2013). Loss of endothelial protein C receptors links coagulation and inflammation to parasite sequestration in cerebral malaria in African children. Blood *122*, 842-851.

Murshudov, G.N., Skubak, P., Lebedev, A.A., Pannu, N.S., Steiner, R.A., Nicholls, R.A., Winn, M.D., Long, F., and Vagin, A.A. (2011). REFMAC5 for the refinement of macromolecular crystal structures. Acta Crystallogr D Biol Crystallogr *67*, 355-367.

Newbold, C., Warn, P., Black, G., Berendt, A., Craig, A., Snow, B., Msobo, M., Peshu, N., and Marsh, K. (1997). Receptor-specific adhesion and clinical disease in Plasmodium falciparum. AmJTropMedHyg *57*, 389-398.

Niang, M., Bei, A.K., Madnani, K.G., Pelly, S., Dankwa, S., Kanjee, U., Gunalan, K., Amaladoss, A., Yeo, K.P., Bob, N.S*., et al.* (2014). STEVOR is a Plasmodium falciparum erythrocyte binding protein that mediates merozoite invasion and rosetting. Cell Host Microbe *16*, 81-93.

Nielsen, M.A., Staalsoe, T., Kurtzhals, J.A., Goka, B.Q., Dodoo, D., Alifrangis, M., Theander, T.G., Akanmori, B.D., and Hviid, L. (2002). *Plasmodium falciparum* variant surface antigen expression varies between isolates causing severe and nonsevere malaria and is modified by acquired immunity. JImmunol *168*, 3444-3450.

Nunes-Silva, S., Dechavanne, S., Moussiliou, A., Pstrag, N., Semblat, J.P., Gangnard, S., Tuikue-Ndam, N., Deleron, P., Chene, A., and Gamain, B. (2015). Beninese children with cerebral malaria do not develop humoral immunity against the IT4-VAR19-DC8 PfEMP1 variant linked to EPCR and brain endothelial binding. MalarJ *14*, 493.

Ochola, L.B., Siddondo, B.R., Ocholla, H., Nkya, S., Kimani, E.N., Williams, T.N., Makale, J.O., Liljander, A., Urban, B.C., Bull, P.C.*, et al.* (2011). Specific receptor usage in Plasmodium falciparum cytoadherence is associated with disease outcome. PLoSOne *6*, e14741.

Oleinikov, A.V., Amos, E., Frye, I.T., Rossnagle, E., Mutabingwa, T.K., Fried, M., and Duffy, P.E. (2009). High throughput functional assays of the variant antigen PfEMP1 reveal a single domain in the 3D7 *Plasmodium falciparum* genome that binds ICAM1 with high affinity and is targeted by naturally acquired neutralizing antibodies. PLoSPathog *5*, e1000386.

Pan, Y., Ma, B., and Nussinov, R. (2005). CD4 binding partially locks the bridging sheet in gp120 but leaves the beta2/3 strands flexible. J Mol Biol *350*, 514-527.

Petersen, J.E., Bouwens, E.A., Tamayo, I., Turner, L., Wang, C.W., Stins, M., Theander, T.G., Hermida, J., Mosnier, L.O., and Lavstsen, T. (2015). Protein C system defects inflicted by the malaria parasite protein PfEMP1 can be overcome by a soluble EPCR variant. Thromb Haemost *114*, 1038-1048.

Rask, T.S., Hansen, D.A., Theander, T.G., Gorm, P.A., and Lavstsen, T. (2010). Plasmodium falciparum erythrocyte membrane protein 1 diversity in seven genomes--divide and conquer. PLoSComputBiol *6*.

Robinson, B.A., Welch, T.L., and Smith, J.D. (2003). Widespread functional specialization of *Plasmodium falciparum* erythrocyte membrane protein 1 family members to bind CD36 analysed across a parasite genome. MolMicrobiol *47*, 1265-1278.

Rogerson, S.J., Tembenu, R., Dobano, C., Plitt, S., Taylor, T.E., and Molyneux, M.E. (1999). Cytoadherence characteristics of Plasmodium falciparum-infected erythrocytes from Malawian children with severe and uncomplicated malaria. AmJTropMedHyg *61*, 467-472.

Rowe, J.A., Moulds, J.M., Newbold, C.I., and Miller, L.H. (1997). P. falciparum rosetting mediated by a parasite-variant erythrocyte membrane protein and complement-receptor 1. Nature *388*, 292-295.

Schindelin, J., Arganda-Carreras, I., Frise, E., Kaynig, V., Longair, M., Pietzsch, T., Preibisch, S., Rueden, C., Saalfeld, S., Schmid, B.*, et al.* (2012). Fiji: an open-source platform for biological-image analysis. NatMethods *9*, 676-682.

Seydel, K.B., Kampondeni, S.D., Valim, C., Potchen, M.J., Milner, D.A., Muwalo, F.W., Birbeck, G.L., Bradley, W.G., Fox, L.L., Glover, S.J.*, et al.* (2015). Brain swelling and death in children with cerebral malaria. N Engl J Med *372*, 1126-1137.

Silamut, K., Phu, N.H., Whitty, C., Turner, G.D., Louwrier, K., Mai, N.T., Simpson, J.A., Hien, T.T., and White, N.J. (1999). A quantitative analysis of the microvascular sequestration of malaria parasites in the human brain. AmJPathol *155*, 395-410.

Snounou, G., Zhu, X., Siripoon, N., Jarra, W., Thaithong, S., Brown, K.N., and Viriyakosol, S. (1999). Biased distribution of *msp1* and *msp2* allelic variants in *Plasmodium falciparum* populations in Thailand. TransRSocTropMedHyg *93*, 369-374.

Stevenson, L., Laursen, E., Cowan, G.J., Bandoh, B., Barfod, L., Cavanagh, D.R., Andersen, G.R., and Hviid, L. (2015). alpha2-Macroglobulin Can Crosslink Multiple Plasmodium falciparum Erythrocyte Membrane Protein 1 (PfEMP1) Molecules and May Facilitate Adhesion of Parasitized Erythrocytes. PLoS Pathog *11*, e1005022.

Storm, J., and Craig, A.G. (2014). Pathogenesis of cerebral malaria--inflammation and cytoadherence. Front Cell Infect Microbiol *4*, 100.

Svergun, D. (1992). Determination of the regularization parameter in indirect-transform methods using perceptual criteria. Journal of Applied Crystallography *25*, 495-503.

Svergun, D., Barberato, C., and Koch, M.H.J. (1995). CRYSOL - a Program to Evaluate X-ray Solution Scattering of Biological Macromolecules from Atomic Coordinates. Journal of Applied Crystallography *28*, 768-773.

Tamura, K., Dudley, J., Nei, M., and Kumar, S. (2007). MEGA4: Molecular Evolutionary Genetics Analysis (MEGA) software version 4.0. Mol Biol Evol *24*, 1596-1599.

Theisen, M., Soe, S., Jessing, S.G., Okkels, L.M., Danielsen, S., Oeuvray, C., Druilhe, P., and Jepsen, S. (2000). Identification of a major B-cell epitope of the Plasmodium falciparum glutamate-rich protein (GLURP), targeted by human antibodies mediating parasite killing. Vaccine *19*, 204-212.

Turner, G.D., Morrison, H., Jones, M., Davis, T.M., Looareesuwan, S., Buley, I.D., Gatter, K.C., Newbold, C.I., Pukritayakamee, S., Nagachinta, B.*, et al.* (1994). An immunohistochemical study of the pathology of fatal malaria. Evidence for widespread endothelial activation and a potential role for intercellular adhesion molecule-1 in cerebral sequestration. AmJPathol *145*, 1057-1069.

Turner, L., Lavstsen, T., Berger, S.S., Wang, C.W., Petersen, J.E., Avril, M., Brazier, A.J., Freeth, J., Jespersen, J.S., Nielsen, M.A.*, et al.* (2013). Severe malaria is associated with parasite binding to endothelial protein C receptor. Nature *498*, 502-505.

Volkov, V.V., and Svergun, D.I. (2003). Uniqueness of ab initio shape determination in small-angle scattering. Journal of Applied Crystallography *36*, 860-864.

Whelan, S., and Goldman, N. (2001). A general empirical model of protein evolution derived from multiple protein families using a maximum-likelihood approach. Mol Biol Evol *18*, 691-699.

Winn, M.D., Ballard, C.C., Cowtan, K.D., Dodson, E.J., Emsley, P., Evans, P.R., Keegan, R.M., Krissinel, E.B., Leslie, A.G., McCoy, A.*, et al.* (2011). Overview of the CCP4 suite and current developments. Acta Crystallogr D Biol Crystallogr *67*, 235-242.
